# Mintaimycins, a Group of Novel Peptide Metabolites from *Micromonospora* sp. C-3509

**DOI:** 10.3390/molecules27041150

**Published:** 2022-02-09

**Authors:** Xiaomin Hu, Ying Wang, Chunyan Zhao, Shufen Li, Xinxin Hu, Xuefu You, Jiajia Shen, Zhen Wang, Bin Hong, Bingya Jiang, Yu Du, Linzhuan Wu

**Affiliations:** 1 NHC Key Laboratory of Biotechnology of Antibiotics, Institute of Medicinal Biotechnology, Chinese Academy of Medical Sciences and Peking Union Medical College, Beijing 100050, China; xiaomin_0903@163.com (X.H.); wangying@imb.cams.cn (Y.W.); chunyanzhao333@163.com (C.Z.); lisf0229@163.com (S.L.); huxinxin1985@163.com (X.H.); 13311123098@163.com (X.Y.); shenjiajia@imb.pumc.edu.cn (J.S.); wangzhen@imb.pumc.edu.cn (Z.W.); hongbin@imb.pumc.edu.cn (B.H.); 2 CAMS Key Laboratory of Synthetic Biology for Drug Innovation, Institute of Medicinal Biotechnology, Chinese Academy of Medical Sciences and Peking Union Medical College, Beijing 100050, China; 3 Beijing Key Laboratory of Antimicrobial Agents, Institute of Medicinal Biotechnology, Chinese Academy of Medical Sciences and Peking Union Medical College, Beijing 100050, China

**Keywords:** *Micromonospora*, mintaimycins, pre-adipocyte differentiation

## Abstract

A group of peptide metabolites (**1**–**4**), designated as mintaimycins, were isolated from *Micromonospora* sp. C-3509. The planar structures of mintaimycins were determined by combination of mass spectrometry, 1D and 2D NMR spectroscopy, and the stereochemistry of mintaimycins were partially resolved by Marfey’s or Mosher’s method. Mintaimycins featured a central *β*-methylphenylalanine or phenylalanine linked at its amino group with 5-methyl-2-hexenoic acid, and at its carboxyl group with 5-hydroxy-norleucine or leucine that combined a derivative of hexanoic acid or 4-methylpentanoic acid. Mintaimycin A_1_ (**1**), the principal component, was found to exhibit the biological activity of inducing pre-adipocyte differentiation of 3T3-L1 fibroblast cells at 10.0 μmol/L.

## 1. Introduction

*Micromonospora*, a genus of actinomycetes, is most famous for producing secondary metabolites of aminocyclitols with strong antibacterial activity and enediynes with severe antitumor activity. *Micromonospora echinospora* subsp. *calichensis*, for example, is the producer of the clinical antibiotic gentamicin and payload in antibody-drug conjugate calicheamicin [1]. *Micromonospora* is also known for producing secondary metabolites with diverse chemical structures and biological activities. Many bioactive polyene macrolactams, aromatic polyketides and peptides have been identified from the genus [2,3,4,5,6,7,8].

We are interested in new secondary metabolites from actinomycetes [9,10,11]. *Micromonospora* sp. C-3509 as a soil strain isolated from Sheshan in Wuhan of China was previously identified as a calicheamicin producer [12]. To explore whether the strain produced any other secondary metabolites, we performed a microbial chemistry investigation for it. Herein, the discovery of a group of novel peptide metabolites (mintaimycins) from the strain was described.

## 2. Results and Discussion

### 2.1. Discovery of New Secondary Metaboltes (Mintaimycins) from Micromonospora sp. C-3509

Two HPLC peaks with identical UV absorption profile (*λ*_max_ at 208 nm) appeared in the ethyl acetate (EtOAc) extract of *Micromonospora* sp. C-3509 cultured on solid state fermentation medium. They displayed molecular mass of 500 and 486 amu, respectively, by mass spectrometry (Figures S1–S3). As calicheamicin, with a molecular mass of 1367 amu, was the only reported secondary metabolite of *Micromonospora* sp. C-3509, compounds in these peaks aroused our interest.

An enlarged culture of *Micromonospora* sp. C-3509 was prepared and extracted with EtOAc for isolation and purification of the interested compound(s) (Figure S4). The EtOAc extract was loaded on an ODS column for fractionation. Eluents containing the interested compound(s) were applied on reverse-phase HPLC column for the final isolation and refinement of the interested compound(s) (Figures S5–S8). In the end, four pure compounds **1**–**4** were obtained (Figures S9–S16).

### 2.2. Structural Elucidation of Mintaimycins *(**1**–**4**)*

Compound **1** was a white amorphous powder. Its molecular formula was deduced as C_29_H_44_N_2_O_5_ by HRESIMS, implying 9 degrees of unsaturation. The ^1^H NMR spectrum of **1** displayed resonances attributable to: (a) a di-substituted trans-double bond attached to an aliphatic methylene unit (*δ*_H_ 5.76 (dt, *J* = 15.6, 1.2 Hz, H-2′), 6.62 (dt, *J* = 15.0, 7.8 Hz, H-3′)), (b) a monosubstituted benzene ring (*δ*_H_ 7.22 (1H, m, H-7), *δ*_H_ 7.27 (2H, m, H-6 and H-8), and *δ*_H_ 7.29 (2H, m, H-5 and H-9)), and (c) six secondary methyls (*δ*_H_ 0.86–1.29, doublets, H-10/H-6′/H-7′/H-1″/H-10″/H-11″). The ^1^H NMR spectrum (Figure S17) also displayed four *sp*^3^-hybridized methylenes, eight *sp*^3^-hybridized methines and three exchangeable resonances assignable to two amide protons at *δ*_H_ 7.09 (d, *J* = 8.4 Hz, N*H*-2) and 7.22 (d, *J* = 10.2 Hz, N*H*-1) and a secondary hydroxy proton at *δ*_H_ 4.16 (ddd, *J* = 8.4, 6.0, 1.8 Hz, H-6″). The ^13^C NMR and DEPT spectra (Figures S18 and S19) of **1** showed 29 carbon resonances, which corresponded to the above groups and three additional carbonyl carbons (*δ*_C_ 166.4 (C-1′), 172.5 (C-1) and 173.8 (C-12″)).

**Figure 1:**
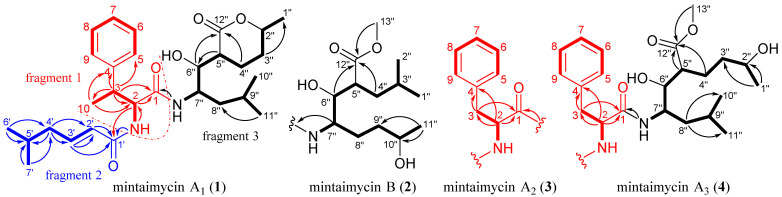
The ^1^H-^1^H COSY (thick lines) and main HMBC (arrows) correlations of mintaimycins A_1–3_ and B (**1**–**4**).

The planar structure of **1** was resolved by a comprehensive analysis of 2D NMR spectra (^1^H-^1^H COSY, HSQC, and HMBC, Figures S20–S22). In the ^1^H-^1^H COSY spectrum (Figure S20) of **1**, the homonuclear coupling correlations of N*H*-2/H-2/H-3/H_3_-10 revealed the presence of structural units containing the vicinal coupled protons (Figure 1, thick lines). In the HMBC spectrum of **1**, two- and three-bond correlations of H_3_-10/C-4 (*δ*_C_ 145.2) and C-2, H-3/C-5 (*δ*_C_ 129.2) and C-1 (*δ*_C_ 172.5) were observed. These correlations, in combination with the shifts of these proton and carbon resonances, demonstrated the presence of a *β*-methylphenylalanine (*β*-MePhe) unit in **1** (fragment 1 in Figure 1). The ^1^H-^1^H COSY correlations of H_3_-7′/H-5′, H_3_-6′/H-5′/H-4′/H-3′/H-2′, and HMBC correlations of H_3_-6′/C-5′ and H_2_-4′, H_3_-7′/C-5′ and C-4′, H_2_-4′/C-3′ and C-2′, and H-3′/C-1′, together with the chemical shift of these carbons, indicated the presence of a 5-methyl-2-hexenoic acid unit in **1** (fragment 2 in Figure 1). Meanwhile, the ^1^H-^1^H COSY correlations of N*H*-7″/H-7″, H_3_-1″/H-2″/H_2_-3″/H_2_-4″/H-5″/H-6″/H-7″/H_2_-8″/H-9″/H_3_-10″, H_3_-11″/H-9″, the HMBC correlations of H-6″ and H_2_-4″/C-12″, H_3_-11″ and H_3_-10″/C-8″ and together with molecular formula revealed the presence of 3(2-amino-1-hydroxy-4-methylpentyl)-6-methyltetrahydropyranone unit in **1** (fragment 3 in Figure 1, containing a *δ*-lactone ring).

The connectivity of fragments 1 and 2 via a nitrogen atom (*N*-2) was clearly demonstrated by the HMBC correlation from N*H*-2 to C-1′ (Figure 1). The association of fragments 1 and 3 via C-1-N (*N*H-7″) was suggested by the HMBC correlations from N*H*-7″ to C-1. Therefore, the planar structure of **1** was determined as in Figure 1, which was further supported by the molecular formula of **1**. A SciFinder search confirmed that **1** was a new compound. Compound **1** as the principal component of **1**–**4** was designated as mintaimycin A_1_. Its NMR data were assigned in Table 1.

Compound **2** was a white amorphous powder. Its molecular formula was deduced by HRESIMS as C_30_H_48_N_2_O_6_, which is CH_3_OH more than **1**. The ^1^H and ^13^C NMR data (Figures S24 and S25) indicated that **2** was a close homologue of **1**. Analysis of the 2D-NMR data (Figures S26–S30) suggested that **2** should be the *δ*-lactone ring-open (hydrolyzed) and methyl esterified derivative of **1**, and the isobutyl attached at C-7″ and the butyl attached at C-5″ in **1** were exchanged in **2**, which was confirmed by ^1^H-^1^H COSY correlations of H_3_-1″/H-3″, and H_3_-2″/H-3″/H_2_-4″/H-5″/H-6″/H-7″/H_2_-8″/H_2_-9″/H-10″/H_3_-11″, HMBC correlations from H-7″ to C-1, H_2_-4″ and H-6″ to C-12″ and OCH_3_-13″ to C-12″. Thus, the planar structure of **2** was depicted as in Figure 1. A SciFinder search indicated that it was the isomer of antibiotic M 9026 factor 3 (Table S1) [13], and they differed only at the *E*/*Z* configuration of carbon-carbon double bond of fragment 1. Compound **2** was designated as mintaimycin B. Its NMR data were assigned in Table 1.

Compound **3** was a white amorphous powder. Its molecular formula C_28_H_42_N_2_O_5_ was determined from the HRESIMS data, which is CH_2_ less than **1**. Compound **3** showed nearly identical NMR data to **1** except for the absence of a doublet methyl at *δ*_C_ 20.7 (*δ*_H_ 1.29, d, *J* = 7.2 Hz, C-10), and presence of an additional methene (*δ*_C_ (37.5, C-3), *δ*_H_ (2.80, dd, *J* = 15.8, 9.0 Hz; 2.88, dd, *J* = 15.8, 6.6 Hz, H-3)) to replace a methine (*δ*_C_ (42.4, C-3), *δ*_H_ (3.19, dq, *J* = 10.2, 6.6 Hz, H-3)) in **1**. Detailed analysis of 1D and 2D NMR data (Figures S31–S37) revealed that **3** was the 3-demethyl derivative of **1**, which was supported by the ^1^H-^1^H COSY correlations (Figure 1) of N*H*-2/H-2/H_2_-3 and the HMBC correlations from N*H*-1 and H_2_-3 to C-1, and from H-2 and H_2_-3 to C-4. Thus, the planar structure of **3** was depicted as in Figure 1. Compound **3** was designated as mintaimycin A_2_. Its NMR data were assigned in Table 1.

Compound **4** was obtained as a white amorphous powder. Its molecular formula was deduced as C_29_H_46_N_2_O_6_ by HRESIMS, which is CH_3_OH more than **3**. Spectroscopic data showed that **4** was a close homologue of **3** except for the signals of a methoxy (*δ*_C_ 52.2, *δ*_H_ 3.63 s, OCH_3_-13″) in **4**. Analysis of 2D-NMR data (Figures S38–S44) suggested that **4** should be the *δ*-lactone ring-open (hydrolyzed) and then methyl esterified derivative of **3**, which was supported by the ^1^H-^1^H COSY correlations (Figure 1) of H_2_-4″/H-5″/H-6″ and the HMBC correlations from OCH_3_-13″ to C-12″. Thus, the planar structure of **4** was depicted as in Figure 1. Compound **4** was designated as mintaimycin A_3_. Its NMR data were assigned in Table 1.

### 2.3. Stereochemistry of Mintaimycins

**Figure 2:**
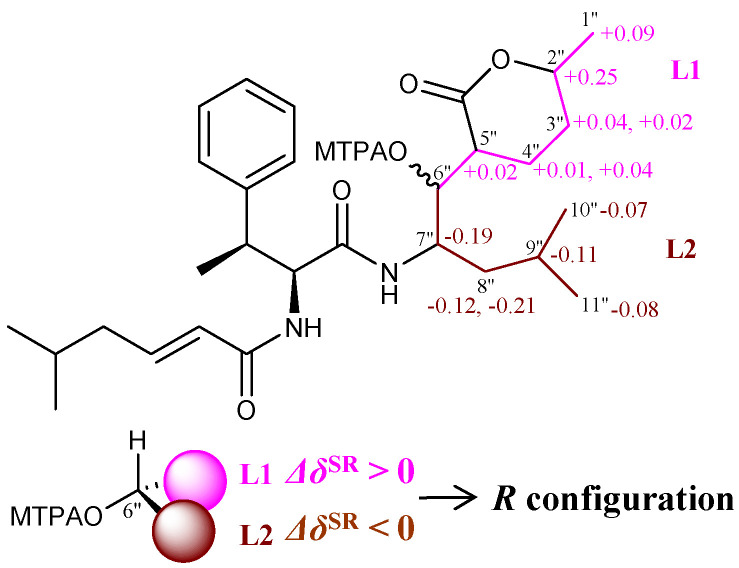
Δ*δ*^SR^ values measured for the MTPA esters of mintaimycin A_1_ (**1**).

Mintaimycins have five or six chiral carbons for determination of their configurations. The chiral carbon(s) in *β*-MePhe/Phe of mintaimycins was determined of configuration by Marfey’s method [14,15]. Specifically, the *β*-MePhe in **1**–**2** was determined as (2*S*, 3*S*)-*β*-MePhe (Figure S45), and the Phe in **3**–**4** was determined as (2*S*)-Phe (L-Phe, Figures S46 and S47). The chiral carbon C-6″ (with a secondary hydroxy group in **1** and **3**) and C-10″ (with a secondary hydroxy group in **2**) were deduced of their configurations by Mosher’s method [16]. Specifically, comprehensive analysis of ^1^H NMR resonances of *R*- and *S*-MTPA ester derivatives (Figures S48–S95) revealed systematic distribution of Δ*δ*^SR^ values (*δ*^S^–*δ*^R^ in ppm), thus establishing *R* configuration for chiral carbon C-6″ in **1** and **3** (Figure 2 and Table S2; Table S3 and Figure S96), and *S* configuration for chiral carbon C-10″ in **2** (Table S4 and Figure S97). According to the plausible pathway proposed for mintaimycins biosynthesis described below, chiral carbon C-6″ in **2** and **4** should take the same configuration as chiral carbon C-6″ in **1** and **3**.

**Figure 3:**
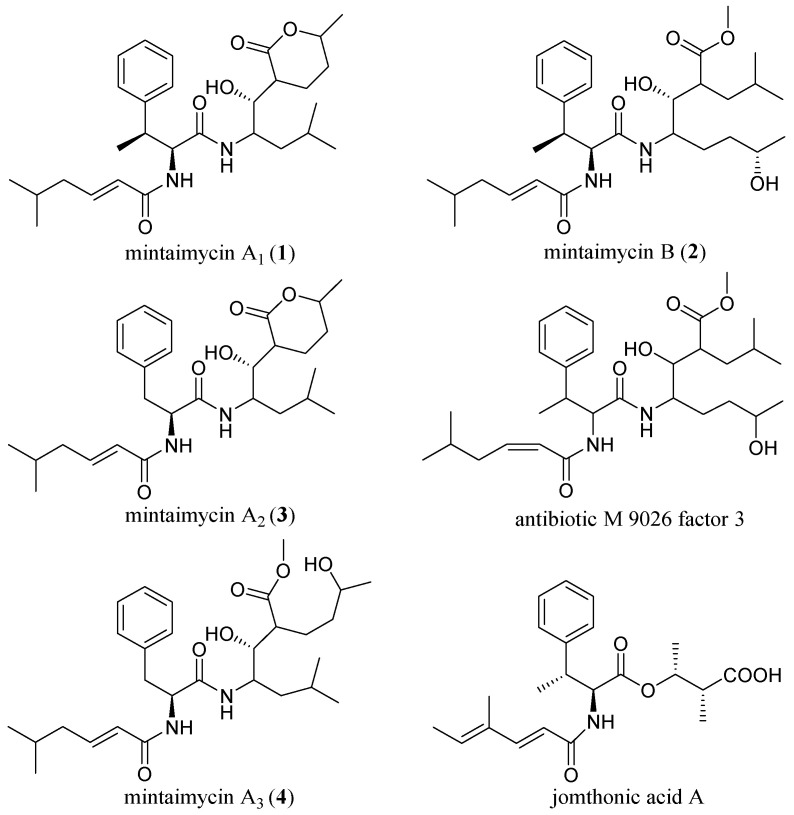
Structures of mintaimycins (**1**–**4**), antibiotic M 9026 factor 3 and jomthonic acid A.

There are still chiral carbons C-2″, C-5″ and C-7″ in **1**, **3** and **4**, C-6″ in **4**, and chiral carbons C-5″, C-6″, C-7″ in **2**, undetermined of their configuration(s). So, mintaimycins with partially elucidated stereochemistry were depicted as in Figure 3.

### 2.4. Speculation of Pathway for Mintaimycins *(**1**–**4**)* Biosynthesis

**Figure 4:**
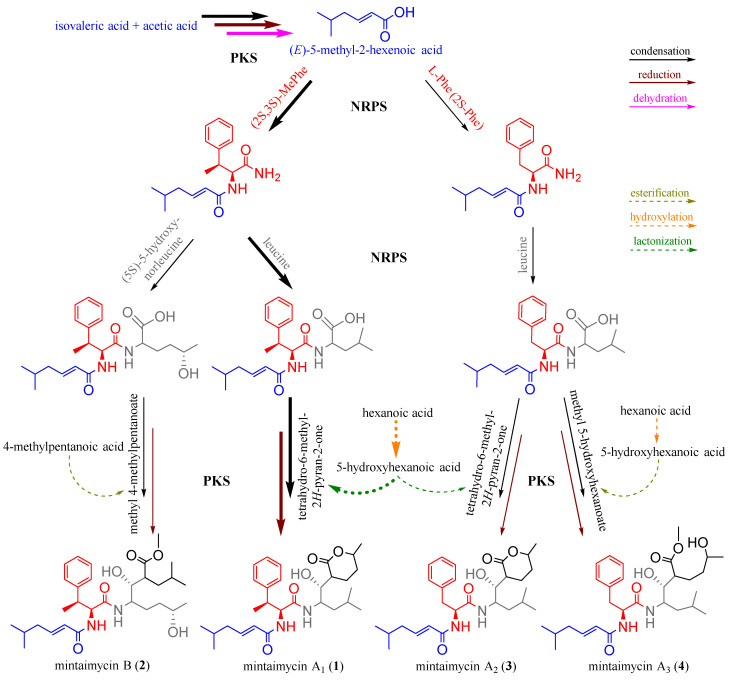
The plausible pathway for mintaimycin biosynthesis.

As a group of peptide metabolites with similar or same building/assembling blocks, mintaimycins must have shared the same biosynthetic mechanism. Based on the biosynthetic pathway of jomthonic acids [17], a group of secondary metabolites from *Streptomyces* with similar structure to mintaimycins, a plausible pathway for mintaimycins biosynthesis was proposed as in Figure 4. In the pathway, fragment 1 as the common start building block may come from condensation, keto-reduction, and dehydration of isovaleric acid and acetic acid (catalyzed by polyketide synthase, PKS). Catalyzed by another PKS, fragment 3 may come from condensation and keto-reduction of 5-hydroxy-norleucine or leucine with a derivative of hexanoic acid or 4-methylpentanoic acid (tetrahydro-6-methyl-2*H*-pyran-2-one, methyl 5-hydroxyhexanoate or methyl 4-methylpentanoate). Fragments 1 and 3 are joined with fragment 2 by two amide bonds catalyzed by non-ribosomal peptide synthase (NRPS). Therefore, mintaimycins belong to the biosynthetic products of NRPS-PKS.

### 2.5. Biological Activities of Mintaimycins

An antibiotic M 9026 complex from *Micromonospora* sp. NRRL 15118 with three antitumor and antimicrobial components was disclosed in the US Patent 4, 692, 333, and planar structure of antibiotic M 9026 factor 3 was provided in SciFinder. As mintaimycins displayed similar structure to antibiotic M 9026 factor 3, we assayed the antibacterial activity of mintaimycins A_1_ (**1**), B (**2**) and A_2_ (**3**), and cytotoxic activity of mintaimycin A_1_ (**1**). Mintaimycin A_3_ (**4**) was not assayed of its biological activity due to lack of material. Unfortunately, mintaimycins A_1_ (**1**), B (**2**) and A_2_ (**3**) showed no activity against Gram-positive and -negative bacteria (MIC ≥ 32 μg/mL, Table S5), and mintaimycin A_1_ (**1**) displayed almost no cytotoxicity against human cell lines HeLa, HGC-27 and PANC-1 (IC_50_ ≥ 172.98 μmol/L, Table S6).

**Figure 5:**
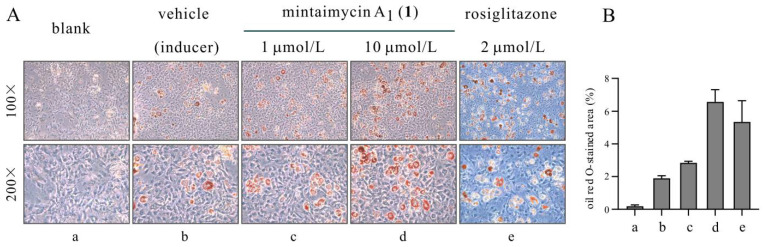
Mintaimycin A_1_ (**1**) induced differentiation of 3T3-L1 fibroblast cells to adipocytes. Compared to vehicle-treated 3T3-L1 fibroblast cells, cells treated with mintaimycin A_1_ (**1**) at 10.0 μmol/L produced prominent oil red O-stained lipid droplets after an incubation period of 14 days, suggesting that it promoted 3T3-L1 fibroblast cells to differentiate into lipid-accumulating adipocytes. Rosiglitazone (an anti-diabetic drug) at 2.0 μmol/L was used as positive control in the assay. A mixture of 10.0 μg/mL of insulin, 0.5 mmol/L of 3-isobutyl-1-methylxanthine and 1.0 μmol/L of dexamethasone was used as an inducer for cell adipogenesis. (**A**) Representative images of oil red O staining of 3T3-L1 cells at day 14, a: blank; b: vehicle (inducer); c: 1.0 μmol/L (**1**); d: 10.0 μmol/L (**1**); e: 2.0 μmol/L (rosiglitazone); (**B**) differentiation-inducing activity quantified by oil red O-stained images (100× magnification).

Mintaimycins are also similar to jomthonic acids (a group of secondary metabolites from *Streptomyces*) that possess the biological activity of inducing pre-adipocyte differentiation [14,18]. We assayed mintaimycin A_1_ (**1**) of this activity. At a concentration of 10.0 μmol/L, mintaimycin A_1_ (**1**) exhibited a prominent activity of inducing pre-adipocytes to mature adipocytes for 3T3-L1 cells (Figure 5, Figure S98).

## 3. Materials and Methods

### 3.1. General Procedures

UV spectra were acquired with a Cary 300 spectrometer. IR spectra were obtained using a Nicolet 5700FTIR microscope spectrometer. Analytical HPLC was conducted on an Agilent system with a 1260 Quat-Pump and DAD detector. For semi-preparative HPLC, a reverse-phase C_18_ column (Spursil 5μm C_18_ column: 250 × 10.0 mm) was used with MeCN-H_2_O as a solvent system. LC-MS was performed on a 1100–6410 Triple Quad from Agilent or an Agilent 1100 LC/MSD with a G1946D single quadrupole mass spectrometer. High-resolution mass spectrometry was carried out on a XEVO G2-XS QTof from Waters. NMR data were collected using a Bruker-600 or an ADVANCE HD 800 MHz and a Bruker Avance Ⅲ HD 700 MHz spectrometer, where chemical shifts (*δ*) were reported in ppm and referenced to DMSO-*d*_6_ solvent signal (*δ*_H_ 2.49 and *δ*_C_ 39.5), CDCl_3_ solvent signal (*δ*_H_ 7.26 and *δ*_C_ 77.0) and acetone-*d*_6_ solvent signal (*δ*_H_ 2.04 and *δ*_C_ 206.0). 3T3-L1 fibroblast cell (pre-adipocyte) line was purchased from the Cell Center of the Institute of Basic Medicine, Chinese Academy of Medical Sciences.

### 3.2. Solid State Fermentation of Micromonospora sp. C-3509

Frozen stock spores of *Micromonospora* sp. C-3509 were thawed, inoculated on slant medium (tryptone 0.5%, yeast extract 0.3%, malt extract 1.0%, K_2_HPO_4_ 0.1%, KH_2_PO_4_ 0.1%, and agar 1.5%, pH 7.0–7.2) and incubated at 28 °C for 10–12 days for spore development. Fresh spores were collected and spread on solid state fermentation medium (sucrose 1.6%, dextrin 2.4%, peptone 0.2%, yeast extract 1.2%, trace element solution 2.0 mL (FeSO_4_·7H_2_O, MnCl_2_·4H_2_O and ZnSO_4_·7H_2_O, each at a concentration of 1.0 mg/mL), KI 0.02% and agar 1.5%, pH 7.0) plates, and incubated at 28 °C for 12 days.

### 3.3. Extraction and Isolation of Mintaimycins *(**1**–**4**)*

Solid state fermentation culture (45 L) of *Micromonospora* sp. C-3509 was extracted with an equal volume of EtOAc three times. The EtOAc extract was concentrated under reduced pressure at room temperature, which yielded a dark brown residue (25.7 g). The residue was loaded onto a preparative ODS column (Spherical C_18_, 40–60 μm, 61 × 219 mm) and fractionated with a stepwise gradient of MeOH-H_2_O at a constant flow rate of 18 mL/min, which yielded fractions F1-F55.

HPLC analysis indicated that a mixture of mintaimycins A_1_ (**1**), B (**2**) and A_2_ (**3**) appeared in F39-F42. These fractions were combined and dried (78.0 mg), then applied on a C18 column for repeated semi-preparative HPLC, which yielded pure preparation of mintaimycins A_1_ (**1**, 22.5 mg), B (**2**, 8.5 mg) and A_2_ (**3**, 7.5 mg).

HPLC analysis indicated that mintaimycins A_3_ (**4**) appeared in F37-F38. These fractions were combined and dried (48.0 mg), then applied on a C_18_ column for repeated semi-preparative HPLC, which yielded pure preparation of mintaimycins A_3_ (**4**, 0.4 mg).

Mintaimycin A_1_ (**1**): white amorphous powder; [*α*]^20^_D_ −21.88 (c 0.32, MeOH); UV-visible (MeOH) *λ*_max_ (log *ε*) 206 (4.51) nm; FTIR *ν*_max_ 3297, 3064, 2958, 2872, 1708, 1652, 1544, 1455, 1384, 1332, 1268, 1215, 1136, 1090, 1036, 981, 942, 885, 761, 701 cm^−1^; ^1^H and ^13^C NMR data, see Table 1; HRESIMS *m*/*z* 501.3318 [M + H]^+^ (calcd for C_29_H_45_N_2_O_5_, 501.3328).

Mintaimycin B (**2**): white amorphous powder; [*α*]^20^_D_ −5.26 (c 0.076, MeOH); UV-visible (MeOH) *λ*_max_ (log *ε*) 207 (4.49) nm; FTIR *ν*_max_ 3286, 3064, 2958, 2872, 1736, 1656, 1547, 1454, 1369, 1263, 1212, 1134, 1091, 1058, 981, 886, 761, 701 cm^−1^; ^1^H and ^13^C NMR data, see Table 1; HRESIMS *m*/*z* 533.3591 [M + H]^+^ (calcd for C_30_H_49_N_2_O_6_, 533.3590).

Mintaimycin A_2_ (**3**): white amorphous powder; [*α*]^20^_D_ −23.75 (c 0.08, MeOH); UV-visible (MeOH) *λ*_max_ (log *ε*) 208 (4.47) nm; FTIR *ν*_max_ 3452, 3311, 3063, 2956, 2871, 1713, 1659, 1631, 1541, 1385, 1264, 1089, 981, 945, 887, 743, 698 cm^−1^; ^1^H and ^13^C NMR data, see Table 1; HRESIMS *m*/*z* 487.3175 [M + H]^+^ (calcd for C_28_H_43_N_2_O_5_, 487.3172).

Mintaimycin A_3_ (**4**): white amorphous powder; [*α*]^20^_D_ −40 (c 0.01, MeOH); UV-visible (MeOH) *λ*_max_ (log *ε*) 209 (4.27) nm; FTIR *ν*_max_ 3282, 3065, 2958, 2871, 1734, 1656, 1553, 1498, 1455, 1367, 1263, 1210, 1124, 1069, 1030, 984, 917, 827, 746, 700 cm^−1^; ^1^H and ^13^C NMR data, see Table 1; HRESIMS *m*/*z* 519.3447 [M + H]^+^ (calcd for C_29_H_47_N_2_O_6_, 519.3434).

### 3.4. Stereochemistry of β-Methylphenylalanine (β-MePhe) and Phenylalanine (Phe) Residue in Mintaimycins by Marfey’s Method

Mintaimycins A_1_ (**1**, 50 μg), B (**2**, 50 μg), A_2_ (**3**, 120 μg) and A_3_ (**4**, 120 μg) were hydrolyzed in 6 N HCl (200 μL) at 100 °C for 12 h. Each hydrolysate was dried and dissolved in distilled water (60 μL), then 1 mol/L of NaHCO_3_ (20 μL) and 1% 1-fluoro-2, 4-dinitrophenyl-5-L-leucinamide (L-FDLA, 25 μL, in acetone) was added, followed by an incubation at 40 °C for 1.5 h. Each L-FDLA derivative solution was neutralized with 1 N of HCl (20 μL), then diluted with MeCN (600 μL) for HPLC analysis.

L-FDLA derivatives of commercial (2*R*,3*R*)-*β*-MePhe, (2*R*,3*S*)-*β*-MePhe, (2*S*,3*S*)-*β*-MePhe, (2*S*,3*R*)-*β*-MePhe, D-Phe and L-Phe were also prepared as described above. An HPLC comparison of L-FDLA derivatives from the hydrolysates of **1**–**2** with L-FDLA derivatives of commercial (2*R*,3*R*)-*β*-MePhe, (2*R*,3*S*)-*β*-MePhe, (2*S*,3*S*)-*β*-MePhe, (2*S*,3*R*)-*β*-MePhe was conducted to determine the stereochemistry of *β*-MePhe in mintaimycins A_1_ (**1**) and B (**2**). An HPLC comparison of L-FDLA derivatives from the hydrolysates of mintaimycins A_2_ (**3**) and A_3_ (**4**) with L-FDLA derivatives of commercial D-Phe and L-Phe was performed to determine the stereochemistry of Phe in mintaimycins A_2_ (**3**) and A_3_ (**4**).

### 3.5. Preparation of (R)- and (S)-MTPA Ester Derivatives of Mintaimycins A_1_ *(**1**)*, B *(**2**)* and A_2_ *(**3**)* by Mosher’s Method

To the solutions of **1** (2.0 mg, 0.004 mmol), **2** (1.0 mg, 0.002 mmol) or **3** (1.0 mg, 0.002 mmol) in 200 μL of dry CH_2_Cl_2_ were added (*S*)- or (*R*)-MTPA-Cl (10 equiv) and DMAP (10 equiv). After stirring at 30 °C for 16 h, the solutions were analyzed by LC-MS for ester derivatives (**1R** and **1S**, **2R** and **2S**, **3R** and **3S**). Then, the reaction solutions were evaporated to dryness, and the residues were applied on analytical HPLC column for purification of these ester derivatives. MTPA derivatives (**1R** and **1S**, **2R** and **2S**, **3R** and **3S**) were dissolved in acetone-*d*_6_, CDCl_3_ and DMSO-*d*_6_, respectively, for NMR analysis.

### 3.6. Pre-Adipocyte Differentiation Assay

3T3-L1 fibroblast cells were maintained in high-glucose DMEM medium containing 10% FBS. The cells were cultured in the 12-well plate to reach a confluence state. Two days post-confluency, the assay was initiated by incubating cells with 10% FBS DMEM containing the inducer mixture (0.5 mmol/L of 3-isobutyl-1-methylxanthine, 10.0 μg/mL of insulin, and 1.0 μmol/L of dexamethasone). At the same time, 1.0 and 10.0 μmol/L of mintaimycin A_1_ (**1**) were added, and 2.0 μmol/L of rosiglitazone was used as a positive control. After 3 days, the medium with inducer mixture was replaced by DMEM containing insulin (10.0 μg/mL) and incubated for another 2 days, then the medium was changed with high-glucose DMEM in the presence of 10% FBS every 2 days. After 14 days, the cells were processed for oil red O staining according to standardized protocol [19].

## Figures and Tables

**Table 1:** NMR data of mintaimycins A_1_ (**1**), B (**2**), A_2_ (**3**) and A_3_ (**4**).

	Mintaimycin A_1_ (1)		Mintaimycin B (2)		Mintaimycin A_2_ (3)		Mintaimycin A_3_ (4)	
Position	*δ*_C_, Type	*δ*_H_ (*J* in Hz)	*δ*_C_, Type	*δ*_H_ (*J* in Hz)	*δ*_C_, Type	*δ*_H_ (*J* in Hz)	*δ*_C_, Type	*δ*_H_ (*J* in Hz)
1	172.5, C		170.7, C		171.0, C		172.5, C	
1-NH		7.22, d (10.2)		6.15, d (8.0)		7.88, d (9.6)		7.12, d (8.8)
2	60.8, CH	4.50, dd (10.2, 8.4)	59.1, CH	4.50, t (7.2)	55.0, CH	4.44, m	56.6, CH	4.67, m
3	42.4, CH	3.19, dq (10.2, 6.6)	40.5, CH	3.43, m	37.5, CH_2_	2.88, dd (15.8, 6.6); 2.80, dd (15.8, 9.0)	39.6, CH_2_	2.93, dd (11.9, 7.2); 3.10, dd (11.9, 7.2)
4	145.2, C		141.7, C		137.9, C		139.3, C	
5	129.2, CH	7.29, overlap	127.6, CH	7.24, overlap	128.2, CH	7.25, overlap	130.8, CH	7.26, overlap
6	129.9, CH	7.27, overlap	128.9, CH	7.31, t (7.2)	129.3, CH	7.25, overlap	129.7, CH	7.26, overlap
7	128.0, CH	7.22, m	127.2, CH	7.23, d (7.2)	126.4, CH	7.18, m	127.9, CH	7.19, overlap
8	129.9, CH	7.27, overlap	128.9, CH	7.31, t (7.2)	129.3, CH	7.25, overlap	129.7, CH	7.26, overlap
9	129.2, CH	7.29, overlap	127.6, CH	7.24, overlap	128.2, CH	7.25, overlap	130.8, CH	7.26, overlap
10	20.7, CH_3_	1.29, d (7.2)	18.2, CH_3_	1.30, d (7.2)				
1′	166.4, C		165.8, C		164.9, C		166.5, C	
1′-NH		7.09, d (8.4)		5.77, d (7.2)		8.21, d (8.4)		7.34, d (8.0)
2′	126.5, CH	5.76, dt (15.6, 1.2)	123.7, CH	5.62, d (15.2);	125.4, CH	5.93, d (15.0)	126.7, CH	5.98, d (14.4)
3′	143.9, CH	6.62, td (15.0, 7.8)	145.1, CH	6.70, td (15.2, 7.2)	141.6, CH	6.53, td (15.0, 7.2)	144.0, CH	6.71, dt (14.4, 6.4)
4′	42.4, CH_2_	1.97, m	41.3, CH_2_	2.00, m	40.6, CH_2_	2.00, m	42.5, CH_2_	2.05, overlap
5′	29.3, CH	1.66, m	27.8, CH	1.88, m	27.6, CH	1.67, m	29.3, CH	1.72, m
6′	23.3, CH_3_	0.86, d (6.6)	22.3, CH_3_	0.87, d (6.4)	21.6, CH_3_	0.87, d (6.6)	23.3, CH_3_	0.90, d (6.4)
7′	23.3, CH_3_	0.86, d (6.6)	22.4, CH_3_	0.87, d (6.4)	22.3, CH_3_	0.87, d (6.6)	23.3, CH_3_	0.90, d (6.4)
1″	22.5, CH_3_	1.22, d (6.6)	21.4, CH_3_	0.94, d (6.4)	22.0, CH_3_	1.89, d (6.0)	24.7, CH_3_	1.06, d (6.4)
2″	78.4, CH	4.28, m	23.9, CH_3_	0.93, d (6.4)	76.9, CH	4.27, m	67.3, CH	3.64, overlap
3″	31.7, CH_2_	1.90, m; 1.38, m	24.8, CH	1.64, m	29.8, CH_2_	1.76, m; 1.26, m	38.5, CH_2_	1.37, m; 1.28, m
4″	20.3, CH_2_	1.98, m; 1.89, m	19.5, CH_2_	1.99, m; 1.87, m	18.5, CH_2_	1.83, m; 1.68, m	24.8, CH_2_	1.86, m; 1.72, m
5″	45.3, CH	2.77, ddd (9.0, 7.2, 2.4)	43.8, CH	2.58, m	43.5, CH	2.65, m	49.4, CH	2.51, m
6″	75.3, CH	4.16, ddd (8.4, 6.0, 1.8)	73.8, CH	4.11, overlap	73.0, CH	3.94, ddd (9.0, 6.6, 1.8)	76.2, CH	3.69, m
7″	50.3, CH	4.03, m	49.3, CH	4.10, overlap	48.2, CH	3.64, m	51.3, CH	3.90, m
8″	43.3, CH_2_	1.79, m; 1.38, m	40.8, CH_2_	1.55, m; 1.26, m	41.0, CH_2_	1.46, m; 1.13, m	39.8, CH_2_	1.38, m
9″	26.2, CH	1.79, m	30.3, CH_2_	1.89, m; 1.42, m	24.1, CH	1.14, m	25.5, CH	1.28, m
10″	23.1, CH_3_	0.95, d (6.6)	78.0, CH	4.39, m	22.4, CH_3_	0.74, d (6.6)	22.3, CH_3_	0.78, d (6.4)
11″	25.2, CH_3_	0.93, d (6.6)	22.0, CH_3_	1.31, d (7.2)	24.2, CH_3_	0.74, d (6.6)	25.1, CH_3_	0.80, d (6.4)
12″	173.8, C		173.8, C		172.9, C		176.2, C	
13″-OCH_3_			50.9, CH_3_	3.49, s			52.2, CH_3_	3.63, s

Note: ^1^H and ^13^C NMR spectra were measured in acetone-*d*_6_ for mintaimycin A_1_ (**1**), CDCl_3_ for mintaimycin B (**2**), DMSO-*d*_6_ for mintaimycin A_2_ (**3**), and acetone-*d_6_* for mintaimycin A_3_ (**4**).

## Data Availability

Not applicable.

## Supplementary Materials

The Supplementary Materials are available online. Figure S1: HPLC and MS analysis of EtOAc extract of *Micromonospora* sp. C-3509. Figure S2: LC-MS analysis of mintaimycin A_1_ (**1**). Figure S3: A MS^2^ spectrum of mintaimycin A_1_ (**1**). Figure S4: Flow chart for isolation and purification of mintaimycins (**1**–**4**). Figures S5–S8: UV-visible spectra of mintaimycins (**1**–**4**). Figures S9–S12: IR spectra of mintaimycins (**1**–**4**). Figures S13–S16: HRESIMS spectra of mintaimycins (**1**–**4**). Figure S17: ^1^H NMR spectrum (600 MHz) of mintaimycin A_1_ (**1**) in acetone-*d*_6_. Figures S18–S19: ^13^C NMR spectrum (150 MHz), DEPT spectrum (150 MHz) of mintaimycin A_1_ (**1**) in acetone-*d*_6_. Figures S20–S22: ^1^H-^1^H COSY spectrum (600 MHz), HSQC spectrum (150 MHz), HMBC spectrum (150 MHz) of mintaimycin A_1_ (**1**) in acetone-*d*_6_. Figures S24–S25: ^1^H NMR spectrum (800 MHz), ^13^C NMR spectrum (200 MHz) of mintaimycin B (**2**) in CDCl_3_. Figures S26–S30: DEPT spectrum (150 MHz), ^1^H-^1^H COSY spectrum (800 MHz), HSQC spectrum (200 MHz), HMBC spectrum (200 MHz), ROESY spectrum (800 MHz) of mintaimycin B (**2**) in CDCl_3_. Figures S31–S37: ^1^H NMR spectrum (600 MHz), ^13^C NMR spectrum (150 MHz), DEPT spectrum (150 MHz), ^1^H-^1^H COSY spectrum (600 MHz), HSQC spectrum (150 MHz), HMBC spectrum (150 MHz), ROESY spectrum (600 MHz) of mintaimycin A_2_ (**3**) in DMSO-*d*_6_. Figures S38–S44: ^1^H NMR spectrum (800 MHz), ^13^C NMR spectrum (200 MHz), DEPT spectrum (175 MHz), ^1^H-^1^H COSY spectrum (800 MHz), HSQC spectrum (200 MHz), HMBC spectrum (200 MHz), ROESY spectrum (800 MHz) of mintaimycin A_3_ (**4**) in acetone-*d*_6_. Figure S45: SIM mode (*m/z* 474.1) of mintaimycin A_1_ (**1**) and mintaimycin B (**2**) by Marfey’s method. Figure S46: HPLC analysis of mintaimycin A_3-4_ (**3**-**4**) by Marfey’s method. Figure S47: LC-MS analysis of mintaimycin A_3-4_ (**3**-**4**) by Marfey’s method. Figures S48–S53: LC-MS spectra of (*R*)-MTPA and (*S*)-MTPA esters of mintaimycins (**1**–**3**). Figures S54–S95: NMR data of (*R*)-MTPA and (*S*)-MTPA esters of mintaimycins (**1**-**3**). Figure S96: Δ*δ*^SR^ values measured for the MTPA esters of mintaimycin A_2_ (**3**). Figure S97: Δ*δ*^SR^ values measured for the MTPA esters of mintaimycin B (**2**). Figure S98: Viability of 3T3-L1 fibroblast cells treated with various concentrations of mintaimycin A_1_ (**1**). Table S1: ^1^H NMR data of mintaimycin B (**2**) and the known antibiotic M 9026 factor 3. Table S2: ^1^H NMR data in Acetone-*d*_6_ for the key protons of mintaimycin A_1_ (**1**) and its (*R*)-MTPA and (*S*)-MTPA esters. Table S3: ^1^H NMR data in DMSO-*d*_6_ for the key protons of mintaimycin A_2_ (**3**) and its (*R*)-MTPA and (*S*)-MTPA esters. Table S4: ^1^H NMR data in CDCl_3_ for the key protons of mintaimycin B (**2**) and its (*R*)-MTPA and (*S*)-MTPA esters. Table S5: MIC of mintaimycin A_1_ (**1**), mintaimycin B (**2**) and mintaimycin A_2_ (**3**) against Gram- positive and negative bacterial strains. Table S6: IC_50_ of mintaimycin A_1_ (**1**) against human cell lines.

## Sample Availability

Not available.
