# Supplementary material for: Mintaimycins, a Group of Novel Peptide Metabolites from Micromonospora sp. C-3509
Source: Molecules. 2022 Feb 9;27(4):1150. doi: 10.3390/molecules27041150 (PMC8877661; doi:10.3390/molecules27041150)
Supplement: Supplementary file 1 [file molecules-27-01150-s001.zip › molecules-1550869-supplementary.pdf]

Supplementary material for

Mintaimycins, a Group of Novel Peptide Metabolites from *Micromonospora* sp. C-3509

Xiaomin Hu [1, 2], Ying Wang [1, 2], Chunyan Zhao [1, 2], Shufen Li [1, 2], Xinxin Hu [1, 3], Xuefu You [1, 3], Jiajia Shen [1, 2], Zhen Wang [1, 2], Bin Hong [1, 2], Bingya Jiang [1, 2, *], Yu Du [1, 2, *] and Linzhuan Wu [1, 2, *]

[1] NHC Key Laboratory of Biotechnology of Antibiotics, Institute of Medicinal Biotechnology, Chinese Academy of Medical Sciences and Peking Union Medical College, Beijing 100050, China; xiaomin_0903@163.com (X.H.); wangying@imb.cams.cn (Y.W.); chunyanzhao333@163.com (C.Z.); lisf0229@163.com (S.L.); huxinxin1985@163.com (X.H.); 13311123098@163.com (X.Y.); shenjiajia@imb.pumc.edu.cn (J.S.); wangzhen@imb.pumc.edu.cn (Z.W.); hongbin@imb.pumc.edu.cn (B.H.); jiangbingya@163.com (B.J.); duyu@imb.pumc.edu.cn (Y.D.); wulinzhuan@imb.pumc.edu.cn (L.W.)

[2] CAMS Key Laboratory of Synthetic Biology for Drug Innovation, Institute of Medicinal Biotechnology, Chinese Academy of Medical Sciences and Peking Union Medical College, Beijing 100050, China

[3] Beijing Key Laboratory of Antimicrobial Agents, Institute of Medicinal Biotechnology, Chinese Academy of Medical Sciences and Peking Union Medical College, Beijing 100050, China

* Correspondence: jiangbingya@163.com (B.J.); duyu@imb.pumc.edu.cn (Y.D.); wulinzhuan@imb.pumc.edu.cn (L.W.); Tel.: +86-10-63165283 (L.W.); Fax: +86-10-63017302 (L.W.)

Contents

**Figure S1.**	HPLC and MS analysis of EtOAc extract of *Micromonospora* sp. C-3509.	S4
**Figure S2.**	LC-MS analysis of mintaimycin A_1 (**1**).	S4
**Figure S3.**	A MS^2 spectrum of mintaimycin A_1 (**1**).	S5
**Figure S4.**	Flow chart for isolation and purification of mintaimycins (**1-4**).	S5
**Figure S5.**	UV-visible spectrum of mintaimycin A_1 (**1**).	S6
**Figure S6.**	UV-visible spectrum of mintaimycin B (**2**).	S6
**Figure S7.**	UV-visible spectrum of mintaimycin A_2 (**3**).	S6
**Figure S8.**	UV-visible spectrum of mintaimycin A_3 (**4**).	S7
**Figure S9.**	IR spectrum of mintaimycin A_1 (**1**).	S7
**Figure S10.**	IR spectrum of mintaimycin B (**2**).	S8
**Figure S11.**	IR spectrum of mintaimycin A_2 (**3**).	S8
**Figure S12.**	IR spectrum of mintaimycin A_3 (**4**).	S9
**Figure S13.**	HRESIMS spectrum of mintaimycin A_1 (**1**).	S9
**Figure S14.**	HRESIMS spectrum of mintaimycin B (**2**).	S10
**Figure S15.**	HRESIMS spectrum of mintaimycin A_2 (**3**).	S10
**Figure S16.**	HRESIMS spectrum of mintaimycin A_3 (**4**).	S11
**Figure S17.**	^1H NMR spectrum (600 MHz) of mintaimycin A_1 (**1**) in acetone-d_6.	S11
**Figure S18.**	^{13}C NMR spectrum (150 MHz) of mintaimycin A_1 (**1**) in acetone-d_6.	S12
**Figure S19.**	DEPT spectrum (150 MHz) of mintaimycin A_1 (**1**) in acetone-d_6.	S12
**Figure S20.**	^1H-^1H COSY spectrum (600 MHz) of mintaimycin A_1 (**1**) in acetone-d_6.	S13
**Figure S21.**	HSQC spectrum (150 MHz) of mintaimycin A_1 (**1**) in acetone-d_6.	S13
**Figure S22.**	HMBC spectrum (150 MHz) of mintaimycin A_1 (**1**) in acetone-d_6.	S14

Figure S23. NOESY spectrum (600 MHz) of mintaimycin A_1 (**1**) in acetone-d_6. S14
Figure S24. ^{1}H NMR spectrum (800 MHz) of mintaimycin B (**2**) in $CDCl_3$. S15
Figure S25. ^{13}C NMR spectrum (200 MHz) of mintaimycin B (**2**) in $CDCl_3$. S15
Figure S26. DEPT spectrum (150 MHz) of mintaimycin B (**2**) in $CDCl_3$. S16
Figure S27. ^{1}H-^{1}H COSY spectrum (800 MHz) of mintaimycin B (**2**) in $CDCl_3$. S16
Figure S28. HSQC spectrum (200 MHz) of mintaimycin B (**2**) in $CDCl_3$. S17
Figure S29. HMBC spectrum (200 MHz) of mintaimycin B (**2**) in $CDCl_3$. S17
Figure S30. ROESY spectrum (800 MHz) of mintaimycin B (**2**) in $CDCl_3$. S18
Table S1. ^{1}H NMR data of mintaimycin B (**2**) and the known antibiotic M 9026 factor 3. S19
Figure S31. ^{1}H NMR spectrum (600 MHz) of mintaimycin A_2 (**3**) in DMSO-d_6. S20
Figure S32. ^{13}C NMR spectrum (150 MHz) of mintaimycin A_2 (**3**) in DMSO-d_6. S20
Figure S33. DEPT spectrum (150 MHz) of mintaimycin A_2 (**3**) in DMSO-d_6. S21
Figure S34. ^{1}H-^{1}H COSY spectrum (600 MHz) of mintaimycin A_2 (**3**) in DMSO-d_6. S21
Figure S35. HSQC spectrum (150 MHz) of mintaimycin A_2 (**3**) in DMSO-d_6. S22
Figure S36. HMBC spectrum (150 MHz) of mintaimycin A_2 (**3**) in DMSO-d_6. S22
Figure S37. ROESY spectrum (600 MHz) of mintaimycin A_2 (**3**) in DMSO-d_6. S23
Figure S38. ^{1}H NMR spectrum (800 MHz) of mintaimycin A_3 (**4**) in acetone-d_6. S23
Figure S39. ^{13}C NMR spectrum (200 MHz) of mintaimycin A_3 (**4**) in acetone-d_6. S24
Figure S40. DEPT spectrum (175 MHz) of mintaimycin A_3 (**4**) in acetone-d_6. S24
Figure S41. ^{1}H-^{1}H COSY spectrum (800 MHz) of mintaimycin A_3 (**4**) in acetone-d_6. S25
Figure S42. HSQC spectrum (200 MHz) of mintaimycin A_3 (**4**) in acetone-d_6. S25
Figure S43. HMBC spectrum (200 MHz) of mintaimycin A_3 (**4**) in acetone-d_6. S26
Figure S44. ROESY spectrum (800 MHz) of mintaimycin A_3 (**4**) in acetone-d_6. S26
Figure S45. SIM mode (*m/z* 474.1) of mintaimycin A_1 (**1**) and mintaimycin B (**2**) by Marfey's method. S27
Figure S46. HPLC analysis of mintaimycin A_{3-4} (**3-4**) by Marfey's method. S28
Figure S47. LC-MS analysis of mintaimycin A_{3-4} (**3-4**) by Marfey's method. S29
Figure S48. LC-MS spectrum of (*R*)-MTPA Ester of mintaimycin A_1 (**1**). S29
Figure S49. LC-MS spectrum of (*S*)-MTPA Ester of mintaimycin A_1 (**1**). S30
Figure S50. LC-MS spectrum of (*R*)-MTPA Ester of mintaimycin B (**2**). S30
Figure S51. LC-MS spectrum of (*S*)-MTPA Ester of mintaimycin B (**2**). S30
Figure S52. LC-MS spectrum of (*R*)-MTPA Ester of mintaimycin A_2 (**3**). S31
Figure S53. LC-MS spectrum of (*S*)-MTPA Ester of mintaimycin A_2 (**3**). S31
Figure S54. ^{1}H NMR spectrum (600 MHz) of (*R*)-MTPA ester of mintaimycin A_1 (**1**) in acetone-d_6. S32
Figure S55. ^{13}C NMR spectrum (150 MHz) of (*R*)-MTPA ester of mintaimycin A_1 (**1**) in acetone-d_6. S32
Figure S56. DEPT spectrum (150 MHz) of (*R*)-MTPA ester of mintaimycin A_1 (**1**) in acetone-d_6. S33
Figure S57. ^{1}H-^{1}H COSY spectrum (600 MHz) of (*R*)-MTPA ester of mintaimycin A_1 (**1**) in acetone-d_6. S33
Figure S58. HSQC spectrum (150 MHz) of (*R*)-MTPA ester of mintaimycin A_1 (**1**) in acetone-d_6. S34
Figure S59. HMBC spectrum (150 MHz) of (*R*)-MTPA ester of mintaimycin A_1 (**1**) in acetone-d_6. S34
Figure S60. NOESY spectrum (600 MHz) of (*R*)-MTPA ester of mintaimycin A_1 (**1**) in acetone-d_6. S35
Figure S61. ^{1}H NMR spectrum (800 MHz) of (*S*)-MTPA ester of mintaimycin A_1 (**1**) in acetone-d_6. S35
Figure S62. ^{13}C NMR spectrum (200 MHz) of (*S*)-MTPA ester of mintaimycin A_1 (**1**) in acetone-d_6. S36
Figure S63. DEPT spectrum (150 MHz) of (*S*)-MTPA ester of mintaimycin A_1 (**1**) in acetone-d_6. S36
Figure S64. ^{1}H-^{1}H COSY spectrum (800 MHz) of (*S*)-MTPA ester of mintaimycin A_1 (**1**) in acetone-d_6. S37
Figure S65. HSQC spectrum (200 MHz) of (*S*)-MTPA ester of mintaimycin A_1 (**1**) in acetone-d_6. S37

**Figure S66.**	HMBC spectrum (200 MHz) of (*S*)-MTPA ester of mintaimycin A_1 (**1**) in acetone-d_6.	S38
**Figure S67.**	ROESY spectrum (800 MHz) of (*S*)-MTPA ester of mintaimycin A_1 (**1**) in acetone-d_6.	S38
**Figure S68.**	^{1}H NMR spectrum (800 MHz) of (*R*)-MTPA ester of mintaimycin B (**2**) in $CDCl_3$.	S39
**Figure S69.**	^{13}C NMR spectrum (200 MHz) of (*R*)-MTPA ester of mintaimycin B (**2**) in $CDCl_3$.	S39
**Figure S70.**	DEPT spectrum (150 MHz) of (*R*)-MTPA ester of mintaimycin B (**2**) in $CDCl_3$.	S40
**Figure S71.**	^{1}H-^{1}H COSY spectrum (800 MHz) of (*R*)-MTPA ester of mintaimycin B (**2**) in $CDCl_3$.	S40
**Figure S72.**	HSQC spectrum (200 MHz) of (*R*)-MTPA ester of mintaimycin B (**2**) in $CDCl_3$.	S41
**Figure S73.**	HMBC spectrum (200 MHz) of (*R*)-MTPA ester of mintaimycin B (**2**) in $CDCl_3$.	S41
**Figure S74.**	ROESY spectrum (800 MHz) of (*R*)-MTPA ester of mintaimycin B (**2**) in $CDCl_3$.	S42
**Figure S75.**	^{1}H NMR spectrum (800 MHz) of (*S*)-MTPA ester of mintaimycin B (**2**) in $CDCl_3$.	S42
**Figure S76.**	^{13}C NMR spectrum (200 MHz) of (*S*)-MTPA ester of mintaimycin B (**2**) in $CDCl_3$.	S43
**Figure S77.**	DEPT spectrum (150 MHz) of (*S*)-MTPA ester of mintaimycin B (**2**) in $CDCl_3$.	S43
**Figure S78.**	^{1}H-^{1}H COSY spectrum (800 MHz) of (*S*)-MTPA ester of mintaimycin B (**2**) in $CDCl_3$.	S44
**Figure S79.**	HSQC spectrum (200 MHz) of (*S*)-MTPA ester of mintaimycin B (**2**) in $CDCl_3$.	S44
**Figure S80.**	HMBC spectrum (200 MHz) of (*S*)-MTPA ester of mintaimycin B (**2**) in $CDCl_3$.	S45
**Figure S81.**	ROESY spectrum (800 MHz) of (*S*)-MTPA ester of mintaimycin B (**2**) in $CDCl_3$.	S45
**Figure S82.**	^{1}H NMR spectrum (600 MHz) of (*R*)-MTPA ester of mintaimycin A_2 (**3**) in DMSO-d_6.	S46
**Figure S83.**	^{13}C NMR spectrum (150 MHz) of (*R*)-MTPA ester of mintaimycin A_2 (**3**) in DMSO-d_6.	S46
**Figure S84.**	DEPT spectrum (150 MHz) of (*R*)-MTPA ester of mintaimycin A_2 (**3**) in DMSO-d_6.	S47
**Figure S85.**	^{1}H-^{1}H COSY spectrum (600 MHz) of (*R*)-MTPA ester of mintaimycin A_2 (**3**) in DMSO-d_6.	S47
**Figure S86.**	HSQC spectrum (150 MHz) of (*R*)-MTPA ester of mintaimycin A_2 (**3**) in DMSO-d_6.	S48
**Figure S87.**	HMBC spectrum (150 MHz) of (*R*)-MTPA ester of mintaimycin A_2 (**3**) in DMSO-d_6.	S48
**Figure S88.**	ROESY spectrum (600 MHz) of (*R*)-MTPA ester of mintaimycin A_2 (**3**) in DMSO-d_6.	S49
**Figure S89.**	^{1}H NMR spectrum (600 MHz) of (*S*)-MTPA ester of mintaimycin A_2 (**3**) in DMSO-d_6.	S49
**Figure S90.**	^{13}C NMR spectrum (150 MHz) of (*S*)-MTPA ester of mintaimycin A_2 (**3**) in DMSO-d_6.	S50
**Figure S91.**	DEPT spectrum (150 MHz) of (*S*)-MTPA ester of mintaimycin A_2 (**3**) in DMSO-d_6.	S50
**Figure S92.**	^{1}H-^{1}H COSY spectrum (600 MHz) of (*S*)-MTPA ester of mintaimycin A_2 (**3**) in DMSO-d_6.	S51
**Figure S93.**	HSQC spectrum (150 MHz) of (*S*)-MTPA ester of mintaimycin A_2 (**3**) in DMSO-d_6.	S51
**Figure S94.**	HMBC spectrum (150 MHz) of (*S*)-MTPA ester of mintaimycin A_2 (**3**) in DMSO-d_6.	S52
**Figure S95.**	NOESY spectrum (600 MHz) of (*S*)-MTPA ester of mintaimycin A_2 (**3**) in DMSO-d_6.	S52
**Table S2.**	^{1}H NMR data in Acetone-d_6 for the key protons of mintaimycin A_1 (**1**) and its (*R*)-MTPA and (*S*)-MTPA esters.	S53
**Table S3.**	^{1}H NMR data in DMSO-d_6 for the key protons of mintaimycin A_2 (**3**) and its (*R*)-MTPA and (*S*)-MTPA esters.	S53
**Figure S96.**	$\Delta\delta^{SR}$ values measured for the MTPA esters of mintaimycin A_2 (**3**).	S53
**Table S4.**	^{1}H NMR data in $CDCl_3$ for the key protons of mintaimycin B (**2**) and its (*R*)-MTPA and (*S*)-MTPA esters.	S54
**Figure S97.**	$\Delta\delta^{SR}$ values measured for the MTPA esters of mintaimycin B (**2**).	S54
**Table S5.**	MIC of mintaimycin A_1 (**1**), mintaimycin B (**2**) and mintaimycin A_2 (**3**) against Gram-positive and negative bacterial strains.	S55
**Figure S98.**	Viability of 3T3-L1 fibroblast cells treated with various concentrations of mintaimycin A_1 (**1**).	S56
**Table S6.**	IC_{50} of mintaimycin A_1 (**1**) against human cell lines.	S56

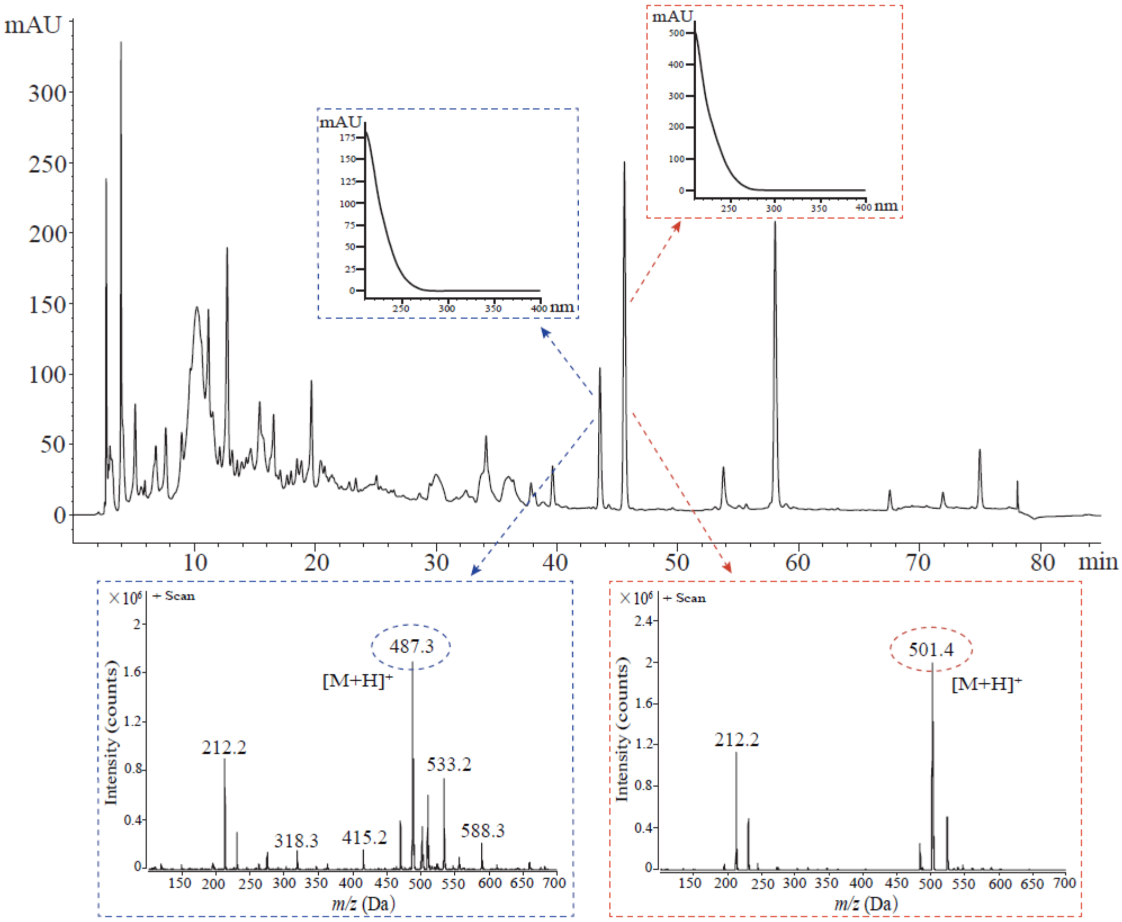

Figure S1. HPLC and MS analysis of EtOAc extract of *Micromonospora* sp. C-3509. HPLC parameters: Diamonsil C_{18} (250 × 4.6 mm, 5μm); MeCN-H_2O, 1.0 mL/min, 5-90% in 65 min; wavelength 230 nm; 28 ℃. Two major HPLC peaks with the same UV absorption profile (λ_{max} at 208 nm) aroused our interests. They displayed molecular mass at 486 and 500 amu, respectively, by mass spectrometry.

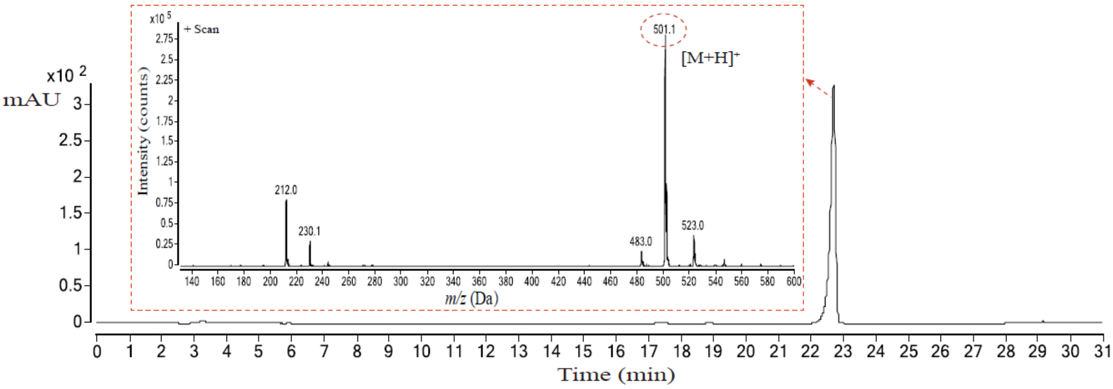

Figure S2. LC-MS analysis of mintaimycin A_1 (**1**).

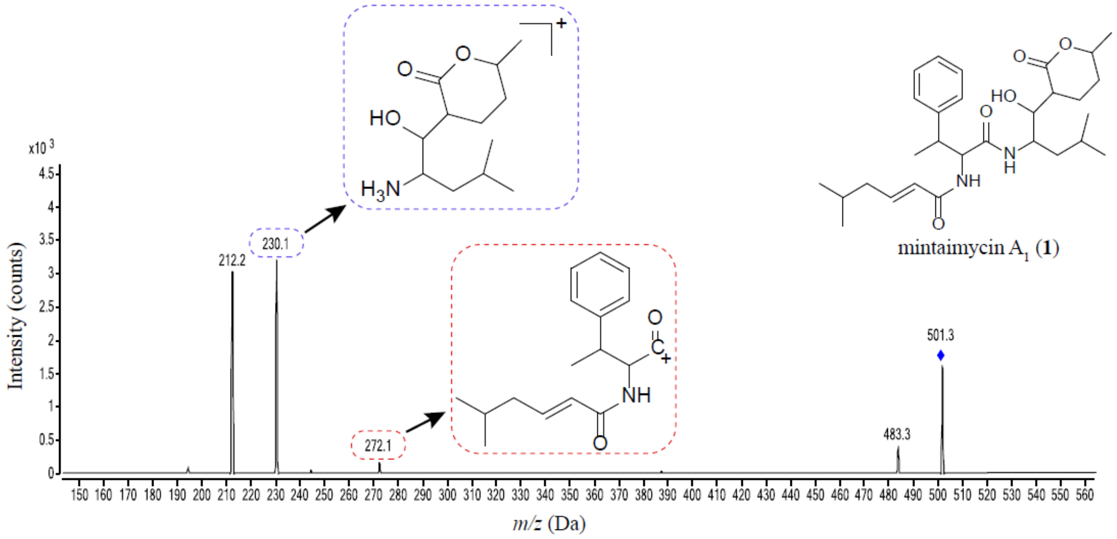

Figure S3. A MS^2 spectrum of mintaimycin A_1 (**1**).

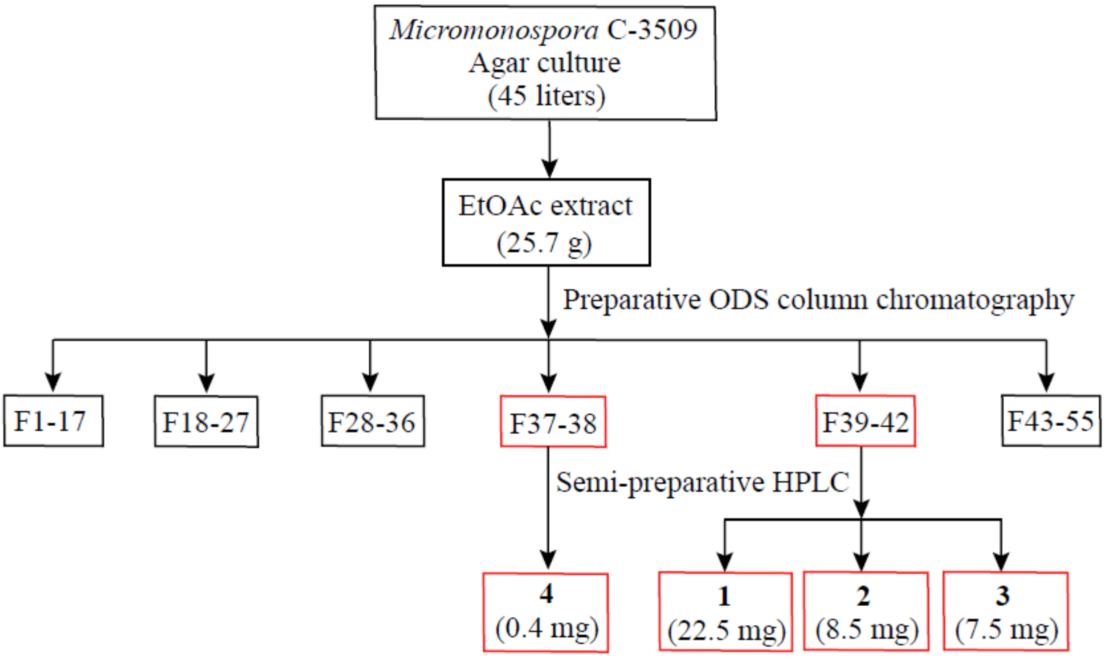

Figure S4. Flow chart for isolation and purification of mintaimycins (**1-4**). Pure preparations of **1-4** were obtained by repeated semi-preparative HPLC (Spursil 5μm C_{18} column: 250 × 10.0 mm, 45% MeCN−H_2O for **1**; 43% MeCN−H_2O for **2**; 44% MeCN−H_2O for **3**; 44% MeCN−H_2O for **4**).

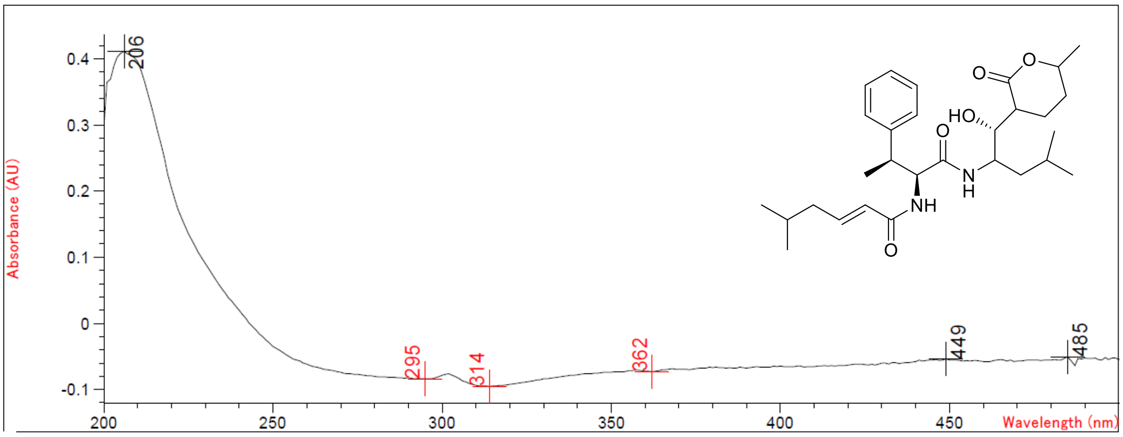

Figure S5. UV-visible spectrum of mintaimycin A_1 (**1**).

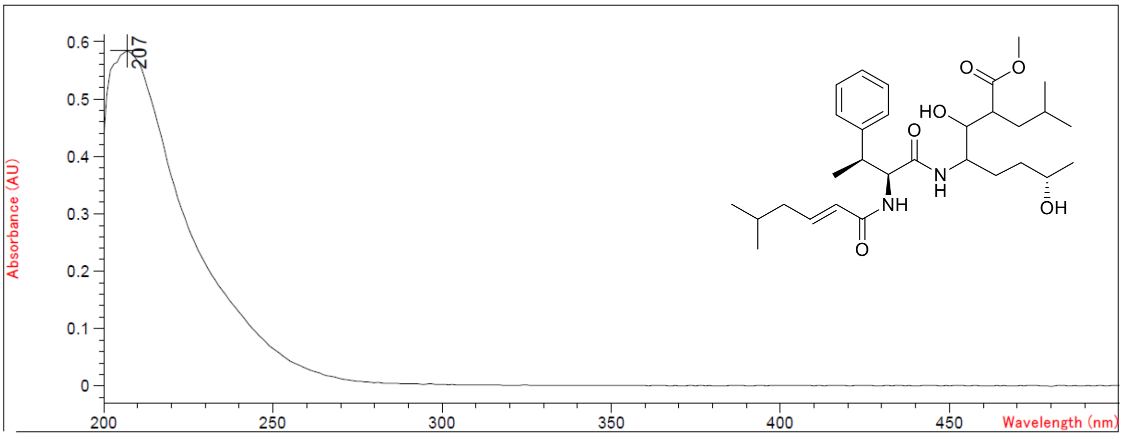

Figure S6. UV-visible spectrum of mintaimycin B (**2**).

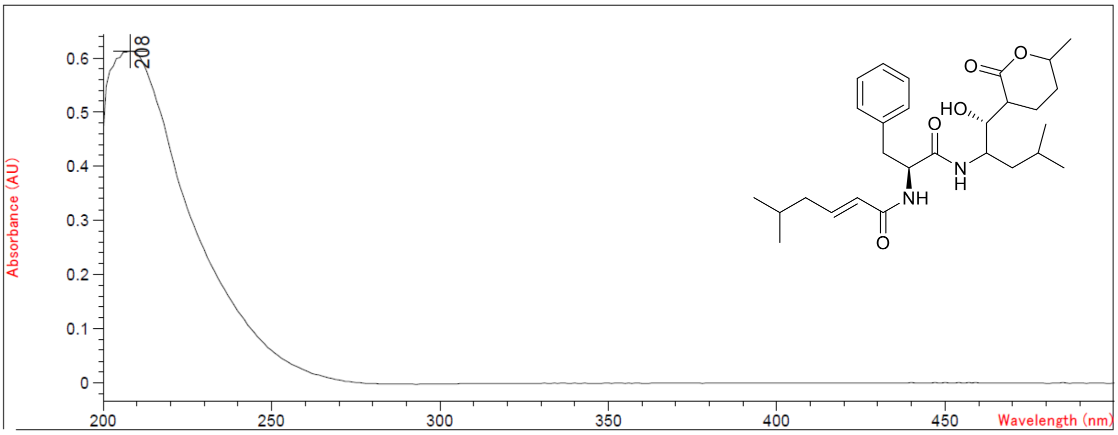

Figure S7. UV-visible spectrum of mintaimycin A_2 (**3**).

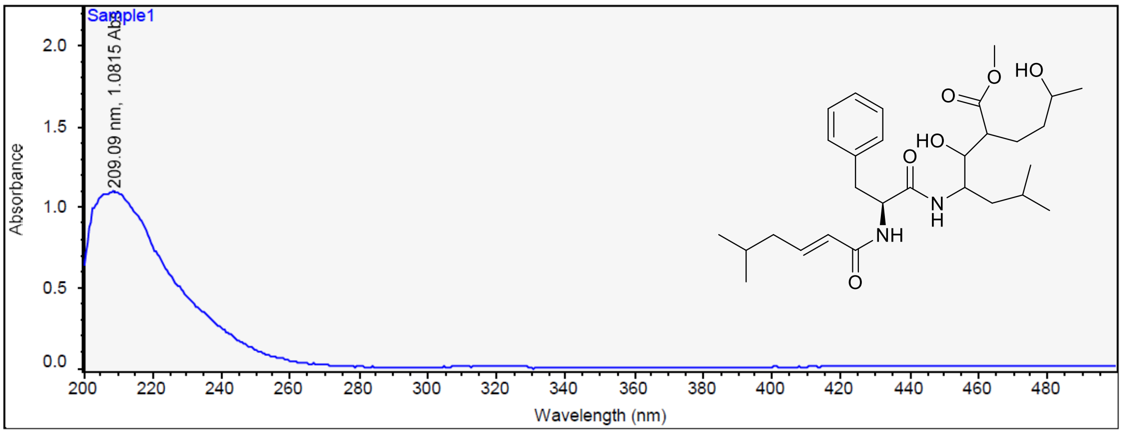

Figure S8. UV-visible spectrum of mintaimycin A$_3$ (**4**).

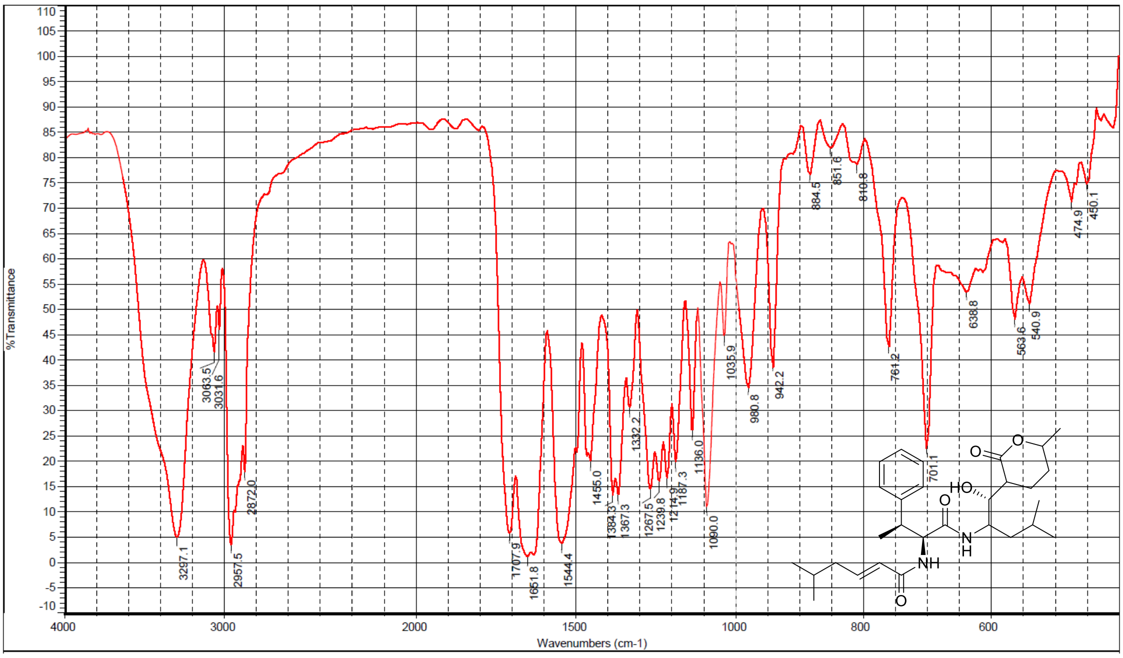

Figure S9. IR spectrum of mintaimycin A$_1$ (**1**).

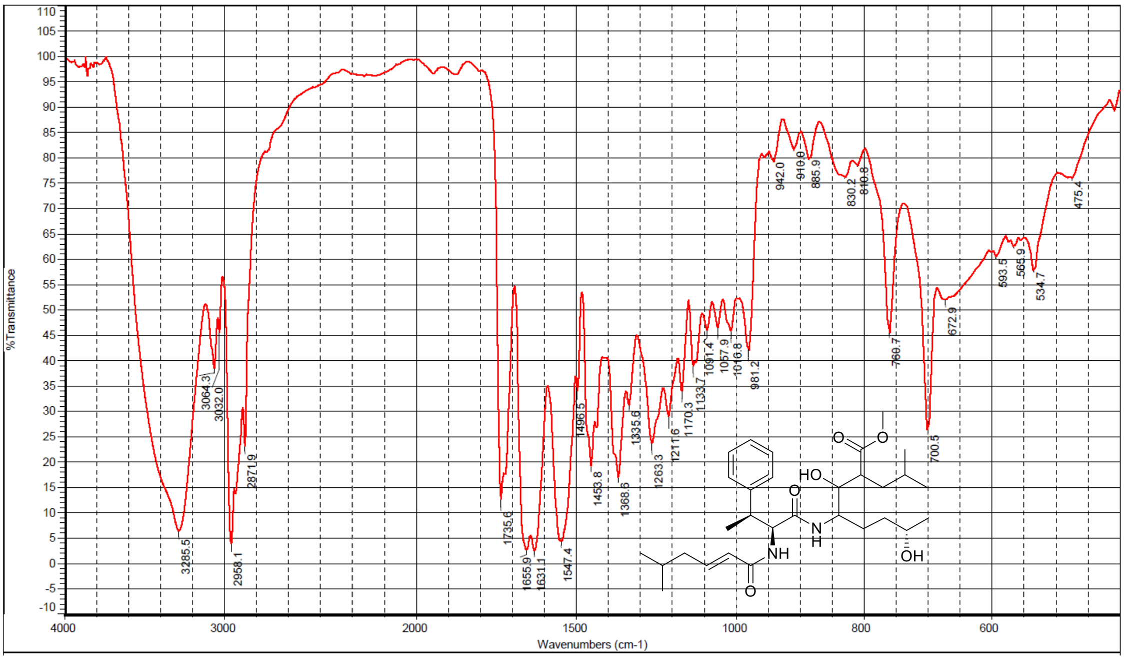

Figure S10. IR spectrum of mintaimycin B (**2**).

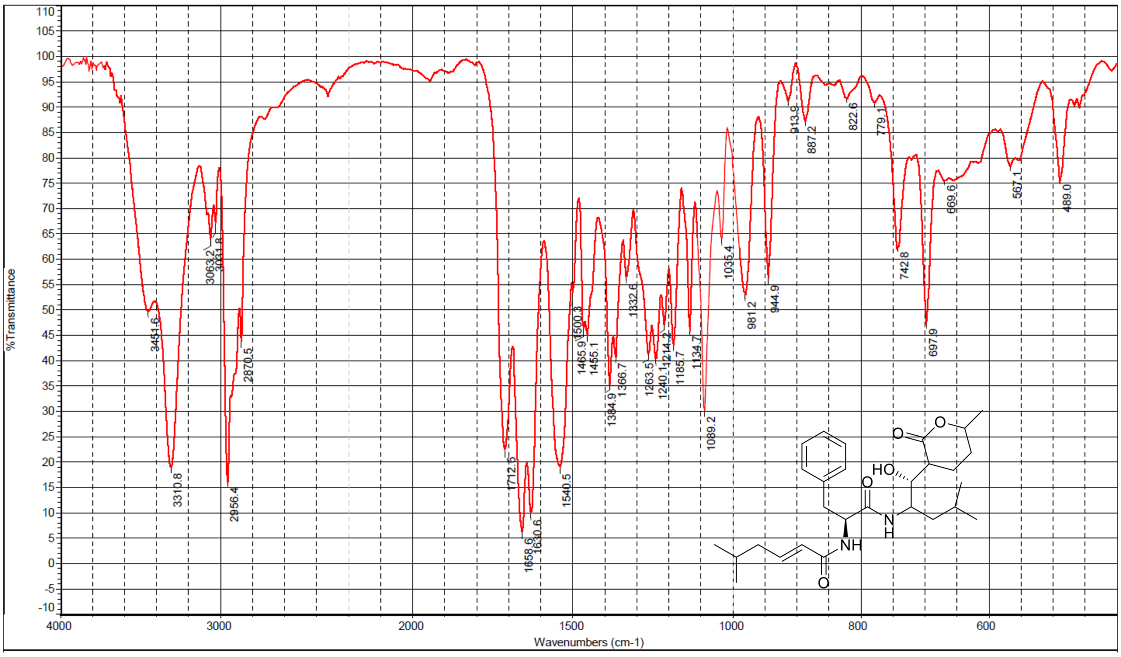

Figure S11. IR spectrum of mintaimycin A_2 (**3**).

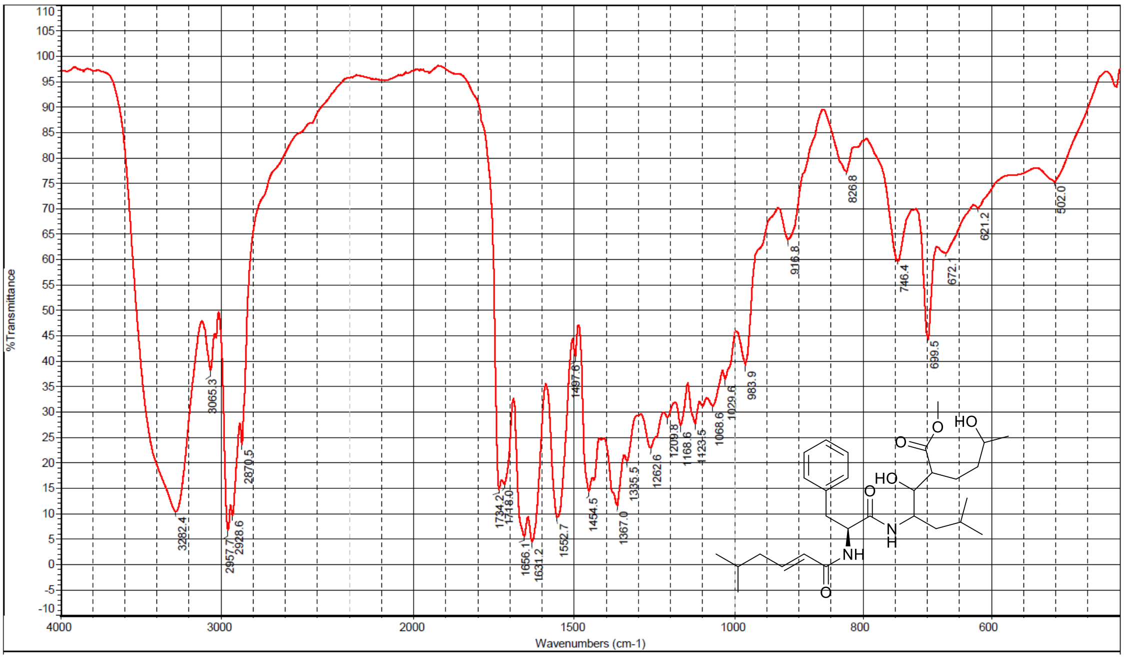

Figure S12. IR spectrum of mintaimycin A_3 (**4**).

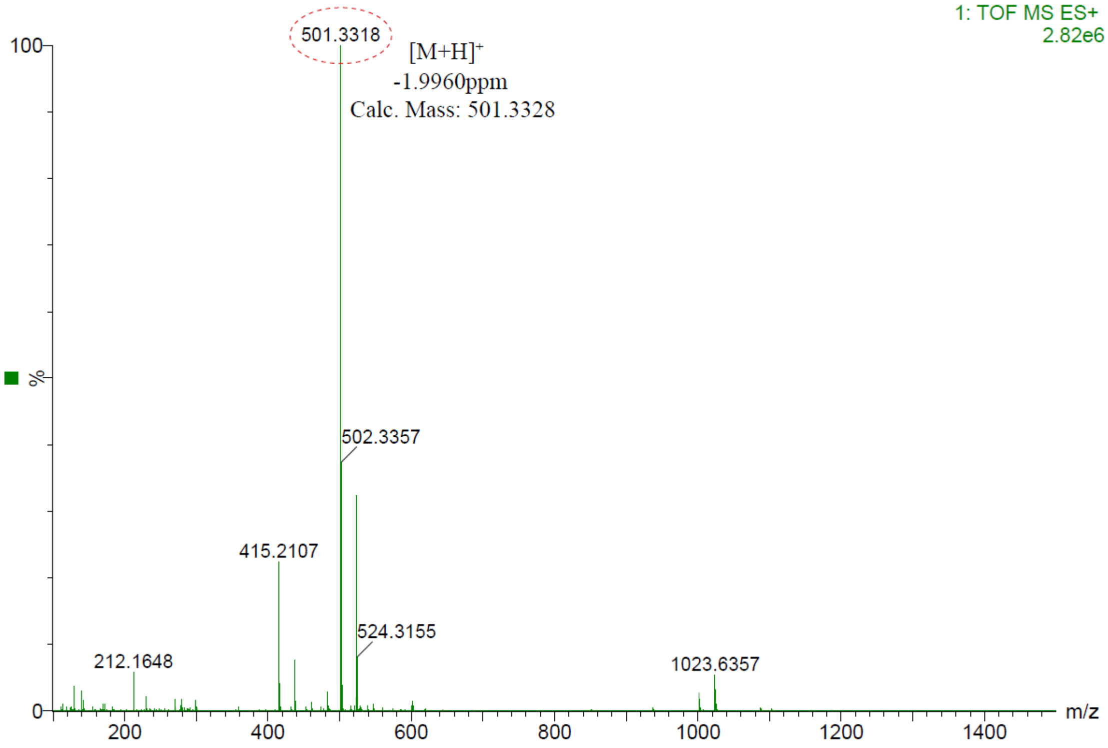

Figure S13. HRESIMS spectrum of mintaimycin A_1 (**1**).

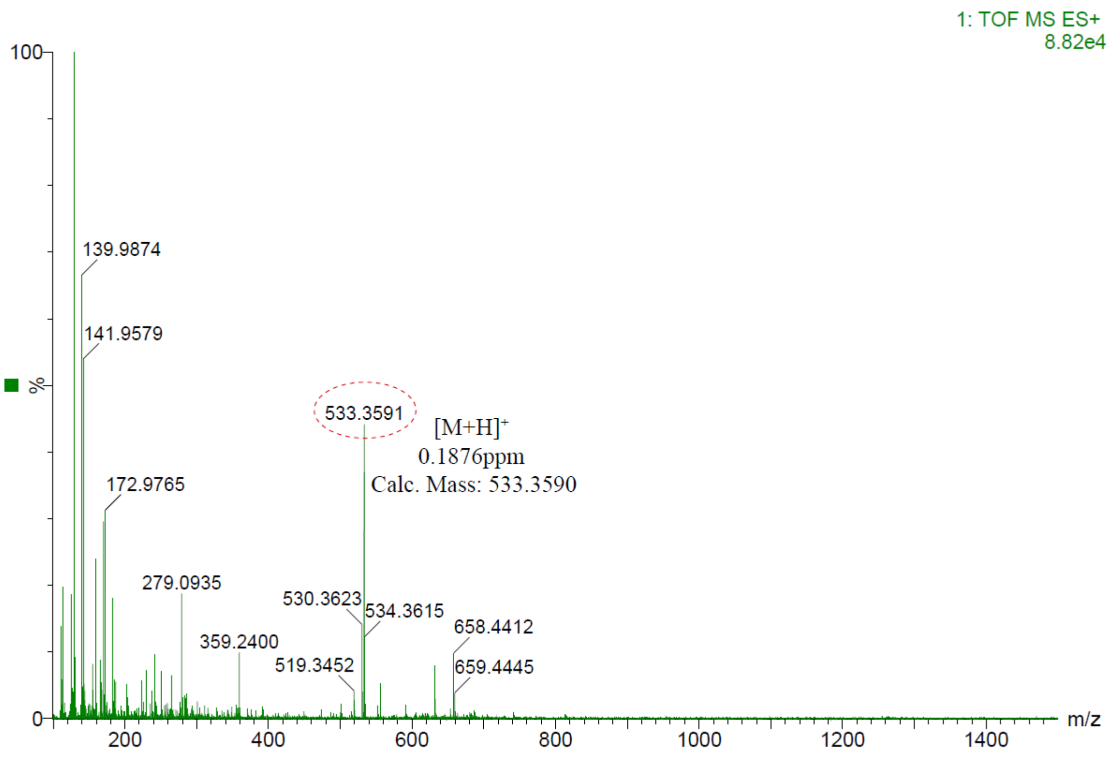

Figure S14. HRESIMS spectrum of mintaimycin B (**2**).

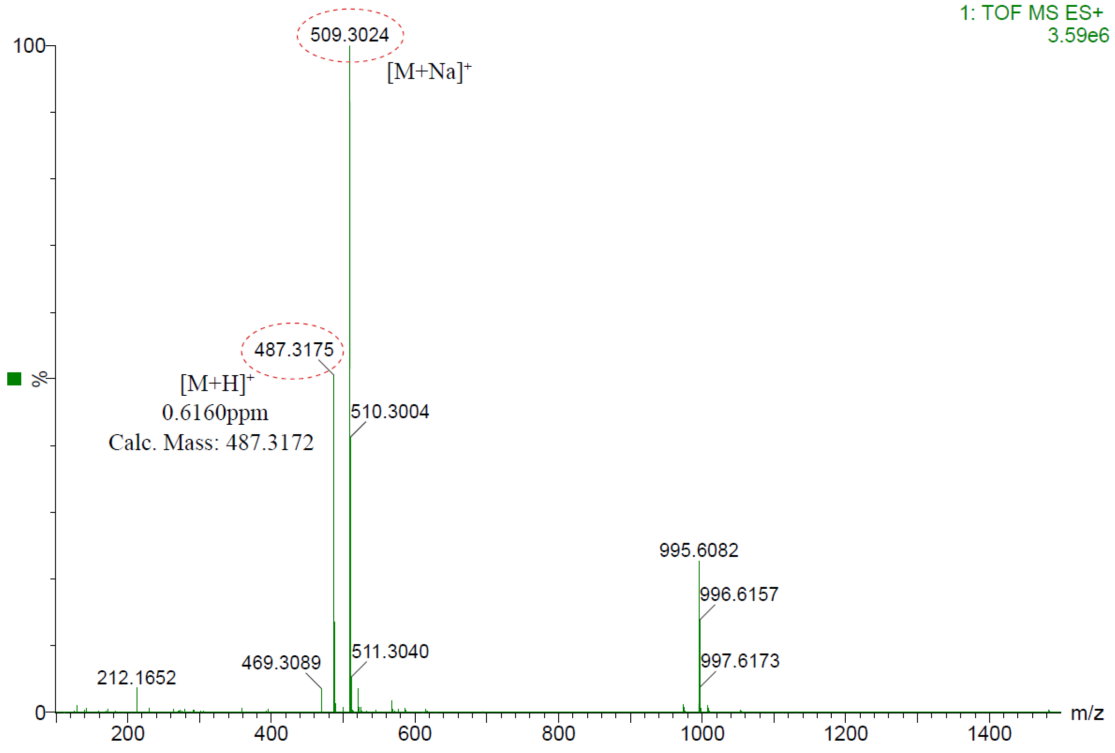

Figure S15. HRESIMS spectrum of mintaimycin A_2 (**3**).

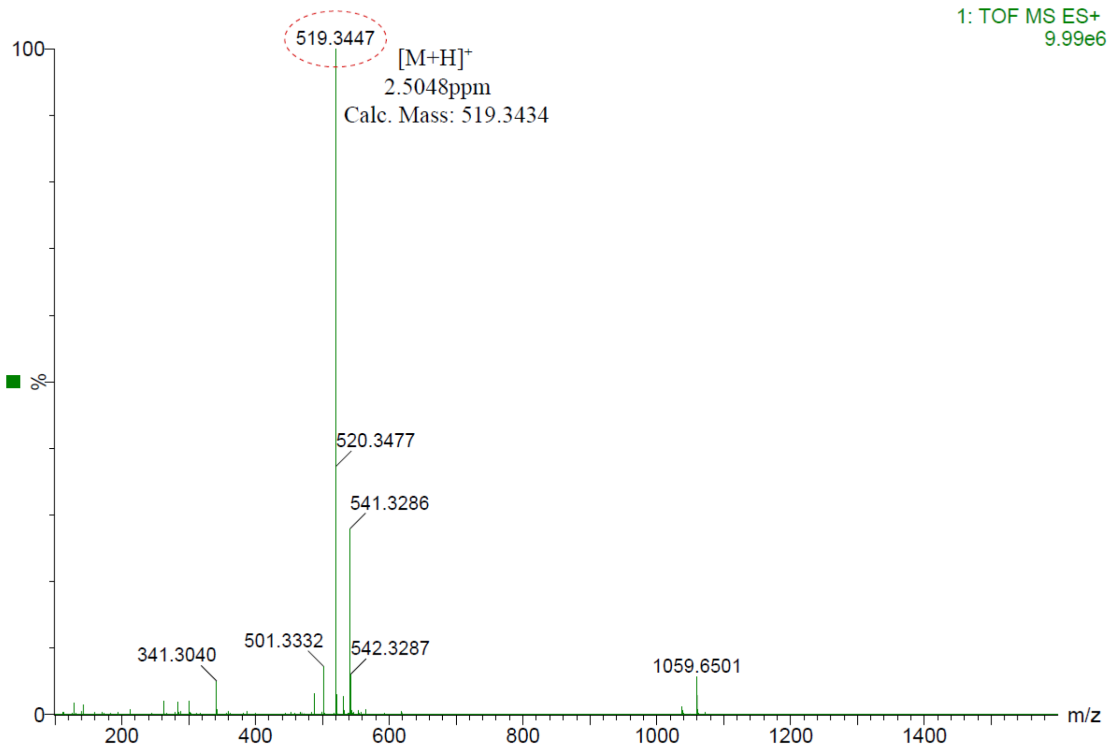

Figure S16. HRESIMS spectrum of mintaimycin A_3 (**4**).

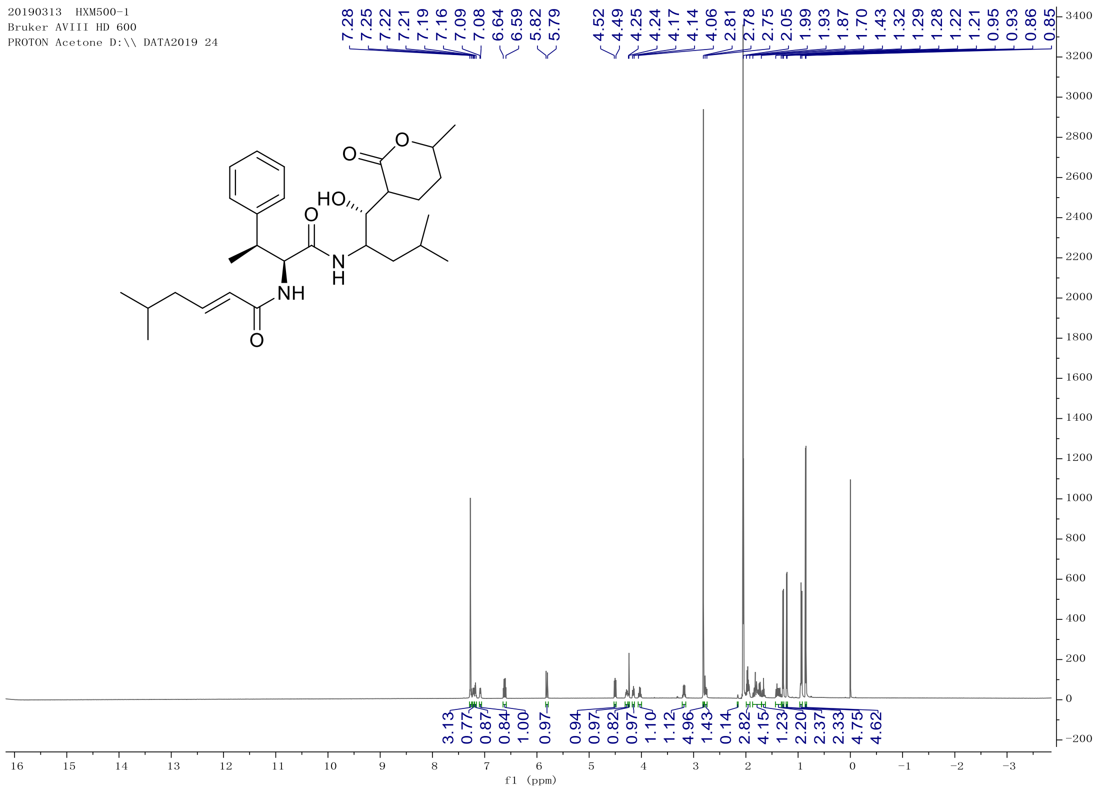

Figure S17. ^{1}H NMR spectrum (600 MHz) of mintaimycin A_1 (**1**) in acetone-d_6.

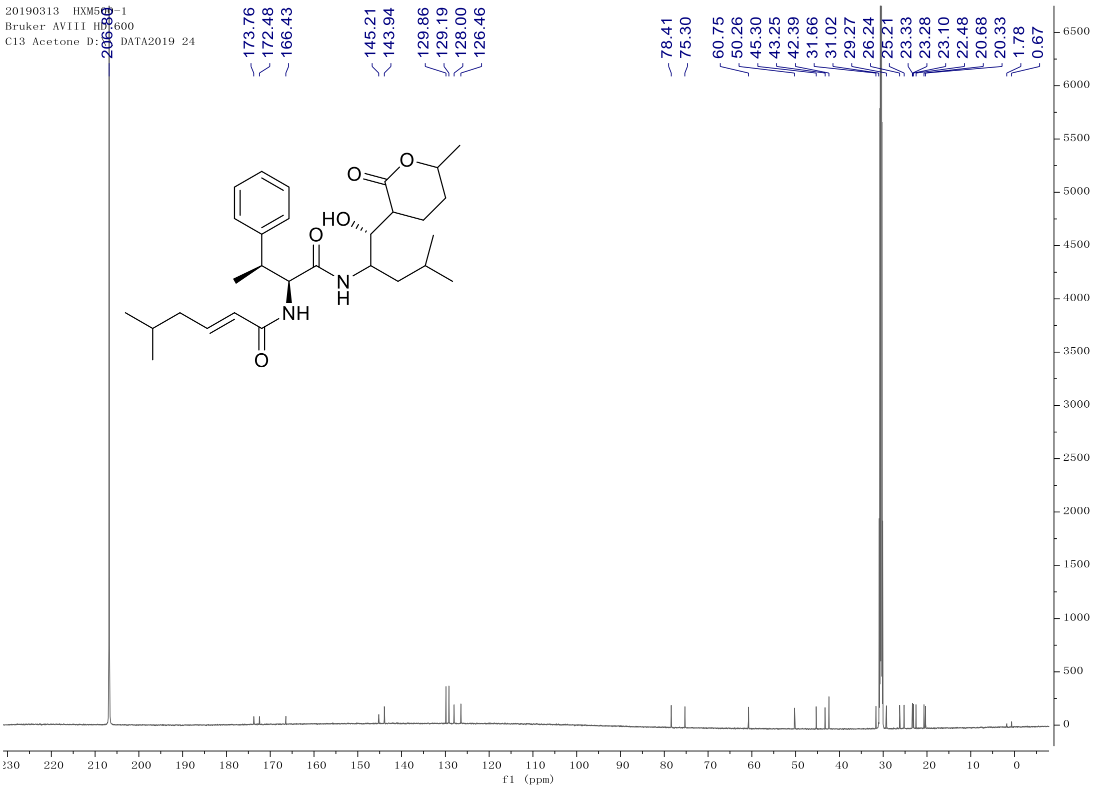

Figure S18. ^{13}C NMR spectrum (150 MHz) of mintaimycin A_1 (**1**) in acetone-d_6.

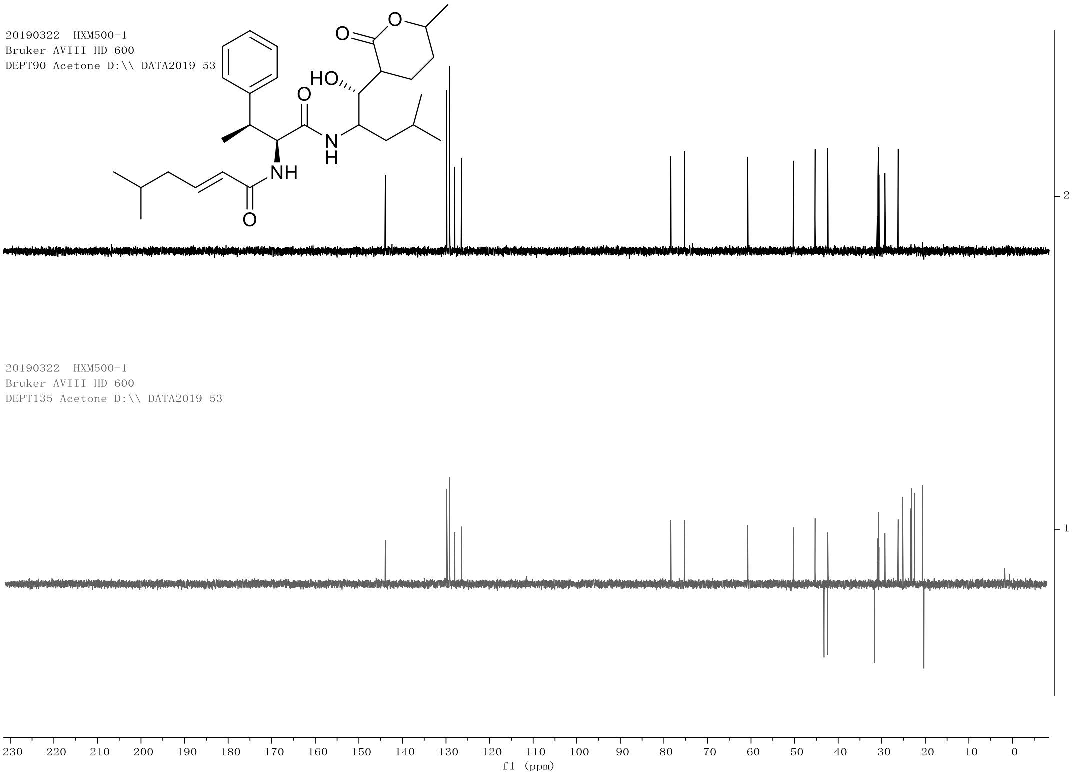

Figure S19. DEPT spectrum (150 MHz) of mintaimycin A_1 (**1**) in acetone-d_6.

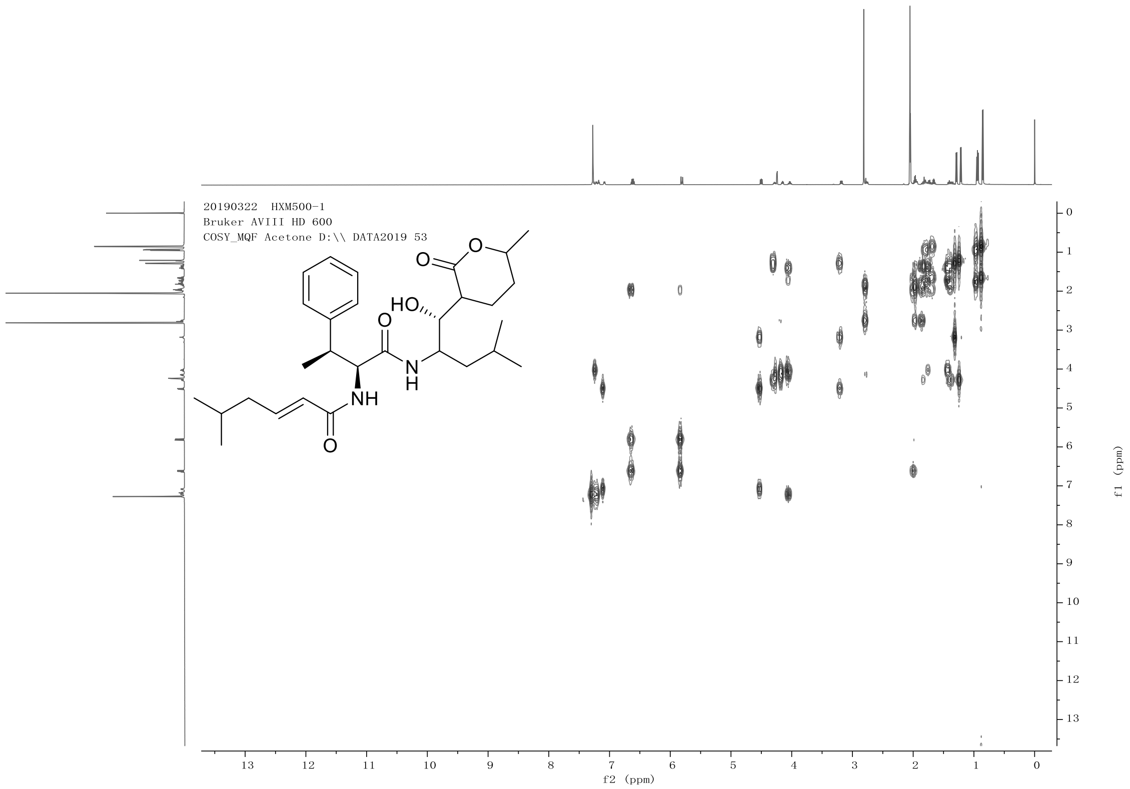

Figure S20. ^{1}H-^{1}H COSY spectrum (600 MHz) of mintaimycin A_1 (**1**) in acetone-d_6.

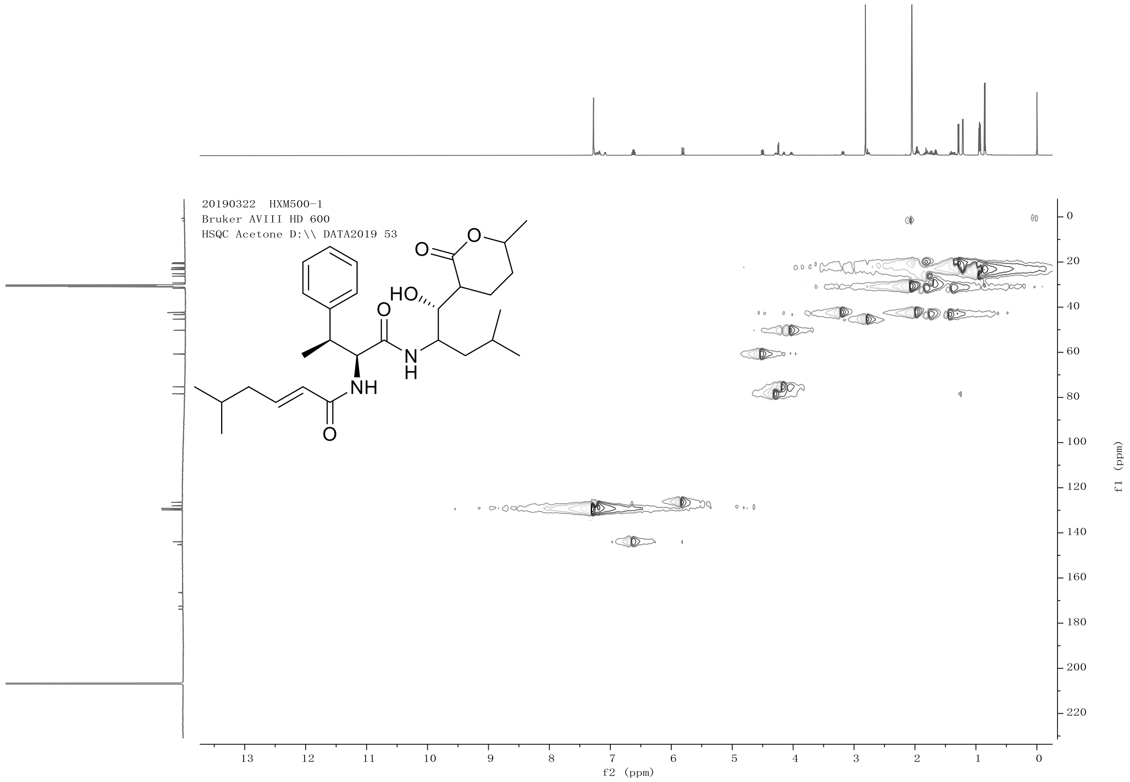

Figure S21. HSQC spectrum (150 MHz) of mintaimycin A_1 (**1**) in acetone-d_6.

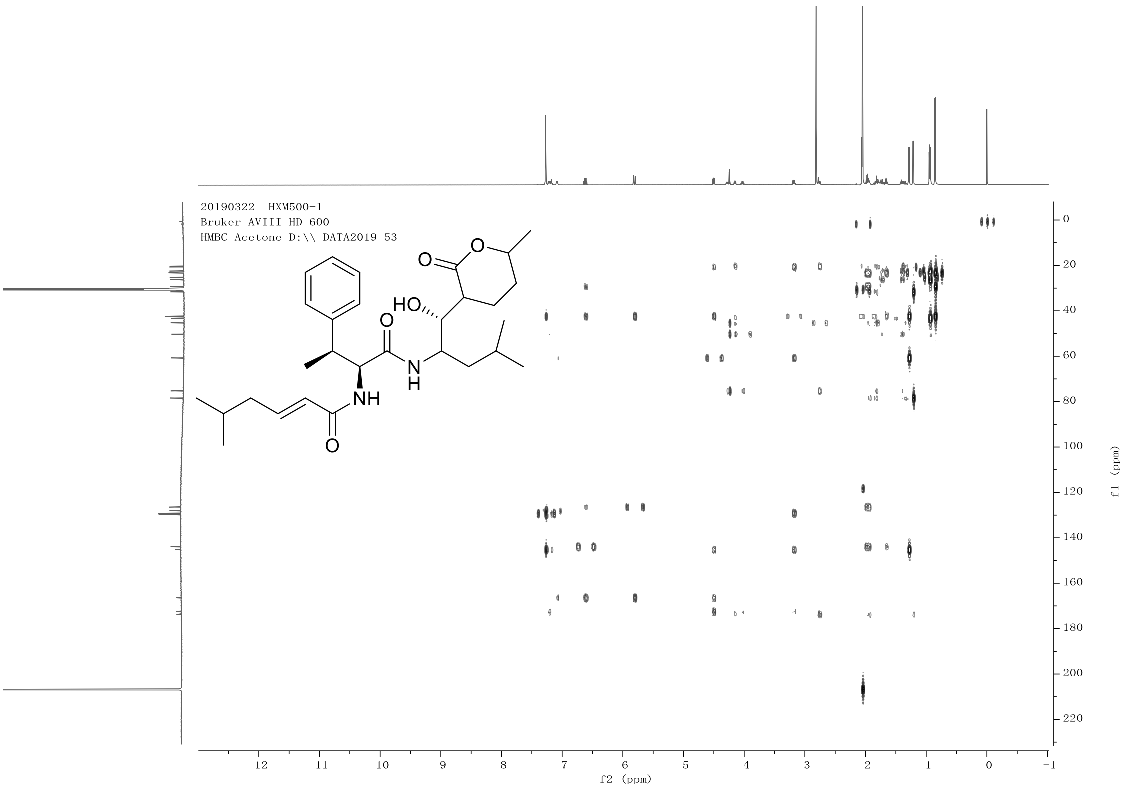

Figure S22. HMBC spectrum (150 MHz) of mintaimycin A_1 (**1**) in acetone-d_6.

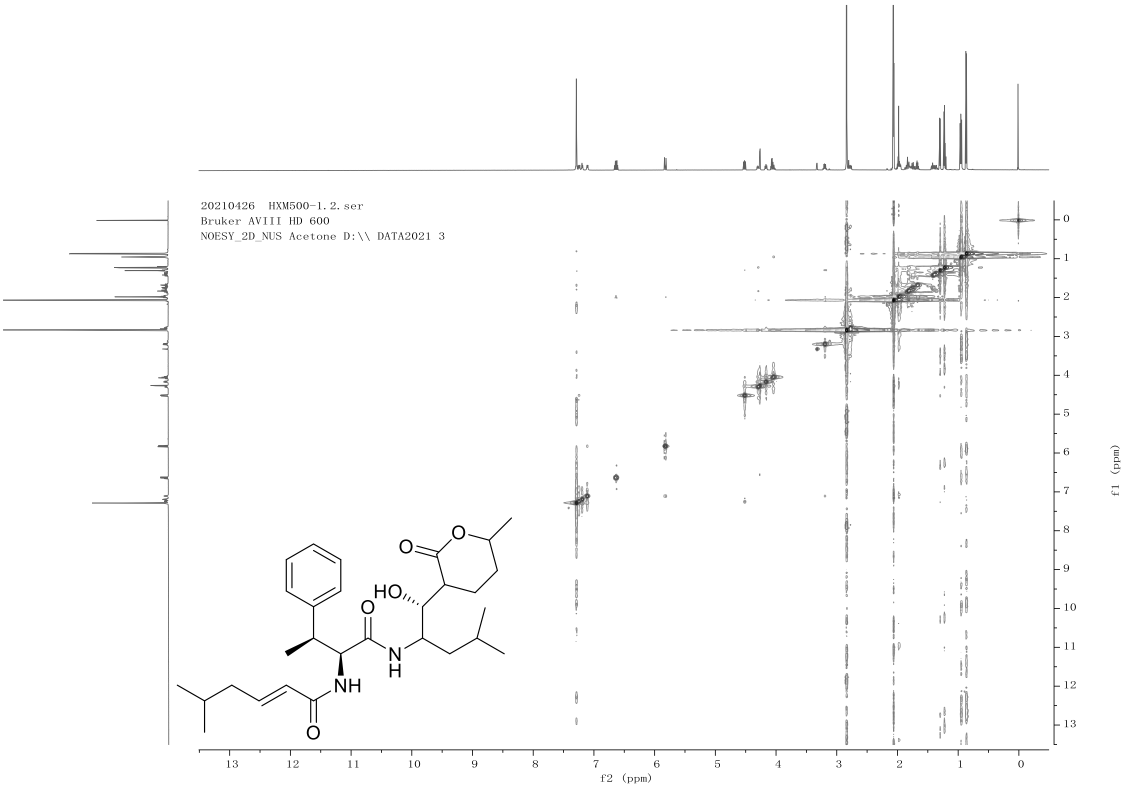

Figure S23. NOESY spectrum (600 MHz) of mintaimycin A_1 (**1**) in acetone-d_6.

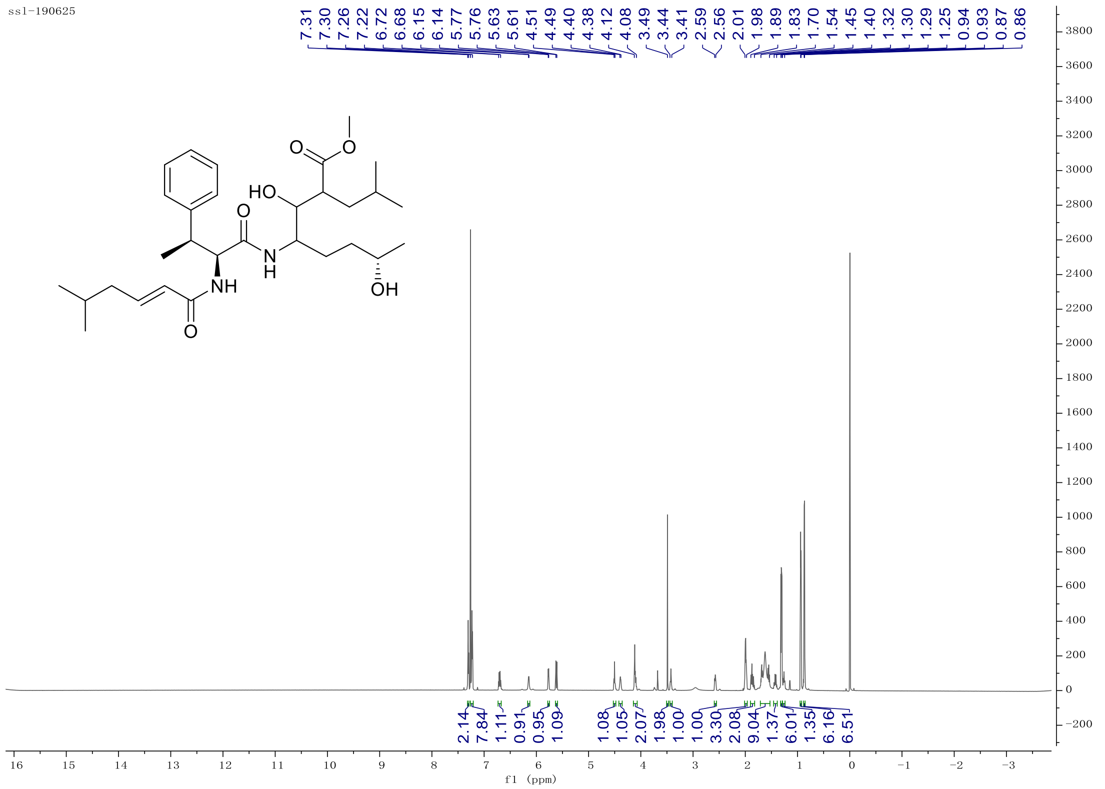

Figure S24. ^{1}H NMR spectrum (800 MHz) of mintaimycin B (**2**) in $CDCl_3$.

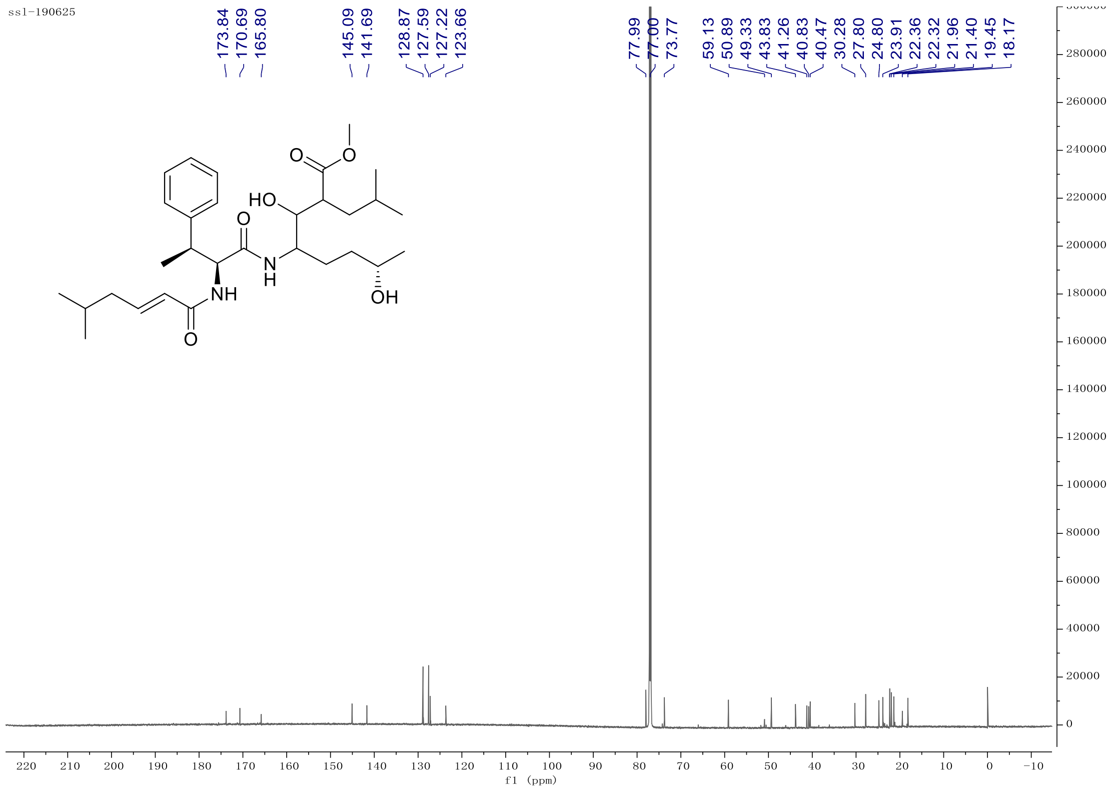

Figure S25. ^{13}C NMR spectrum (200 MHz) of mintaimycin B (**2**) in $CDCl_3$.

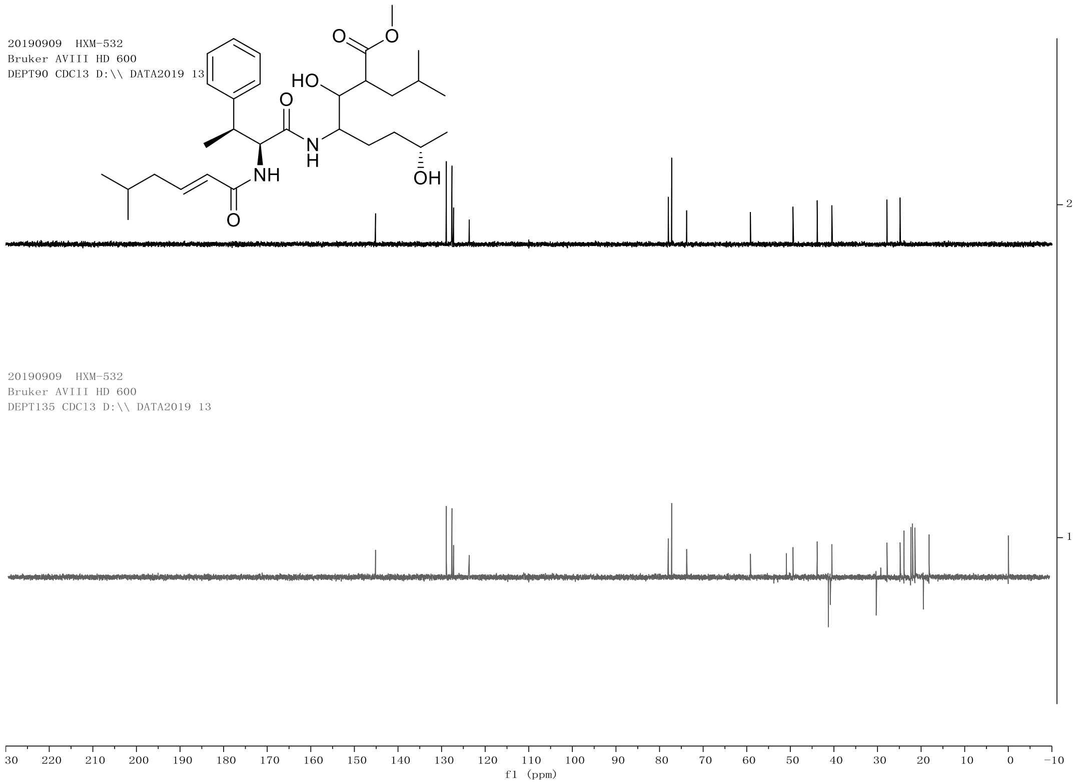

Figure S26. DEPT spectrum (150 MHz) of mintaimycin B (**2**) in $CDCl_3$.

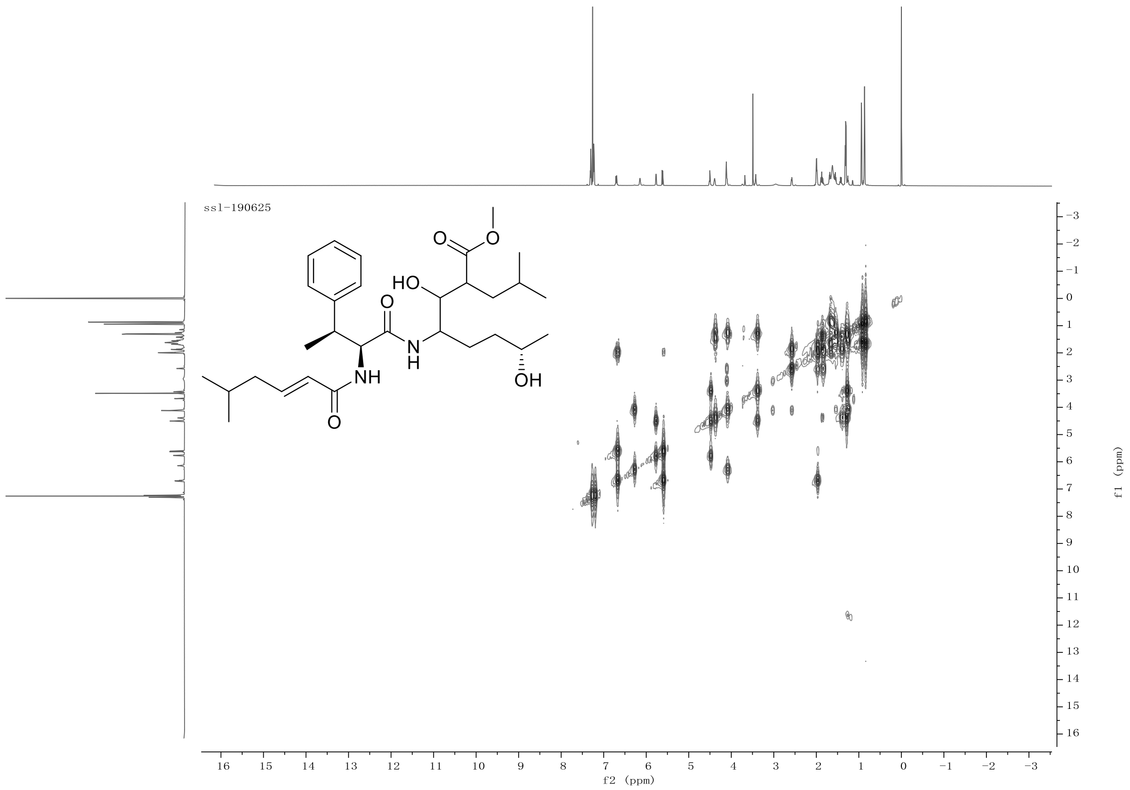

Figure S27. ^{1}H-^{1}H COSY spectrum (800 MHz) of mintaimycin B (**2**) in $CDCl_3$.

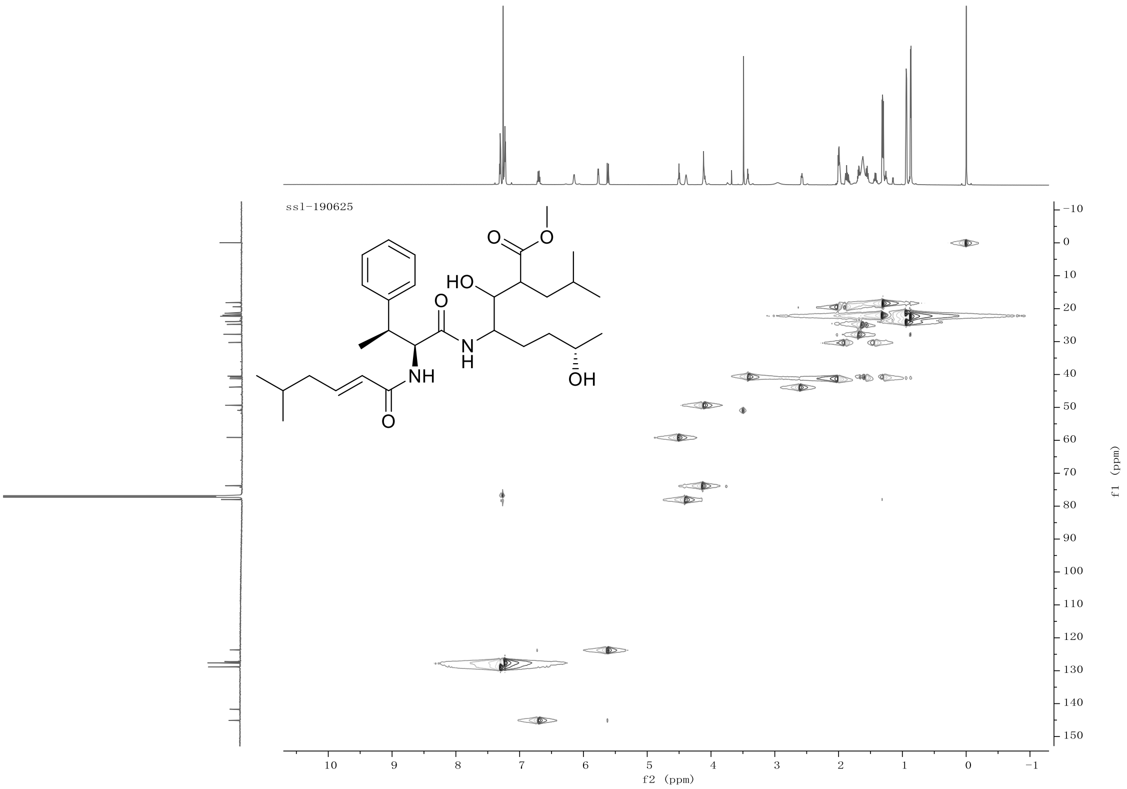

Figure S28. HSQC spectrum (200 MHz) of mintaimycin B (**2**) in $CDCl_3$.

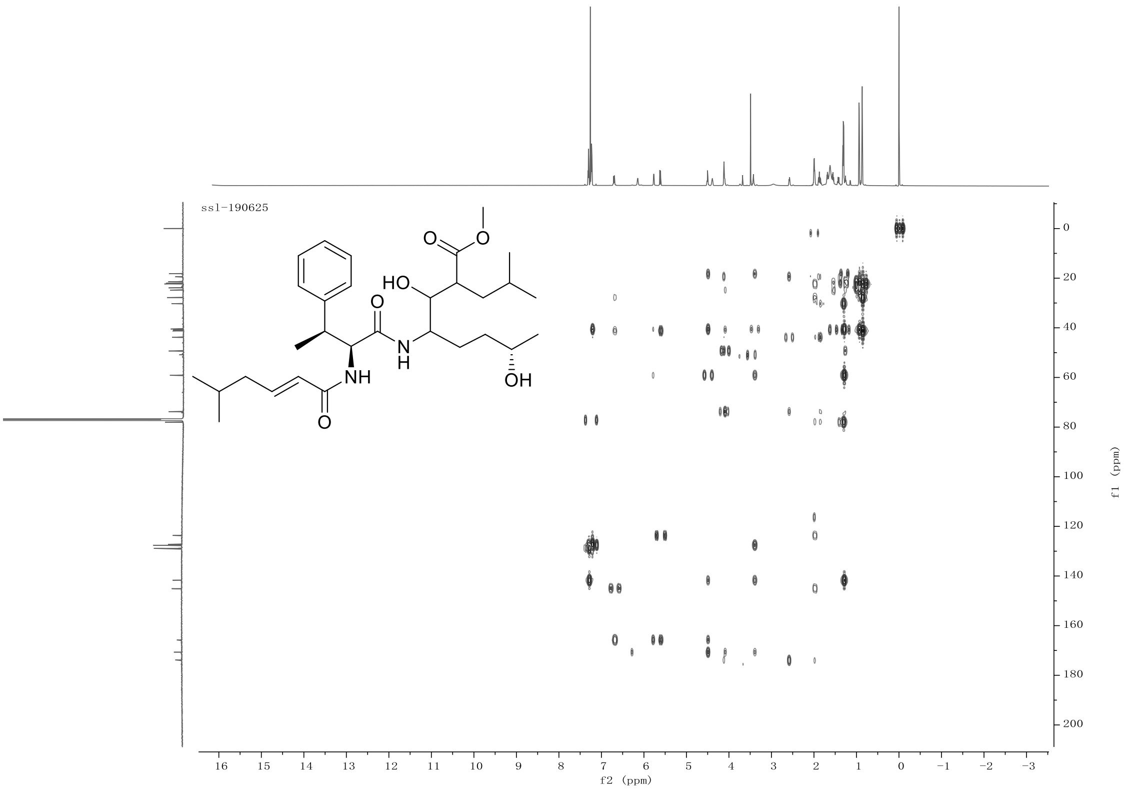

Figure S29. HMBC spectrum (200 MHz) of mintaimycin B (**2**) in $CDCl_3$.

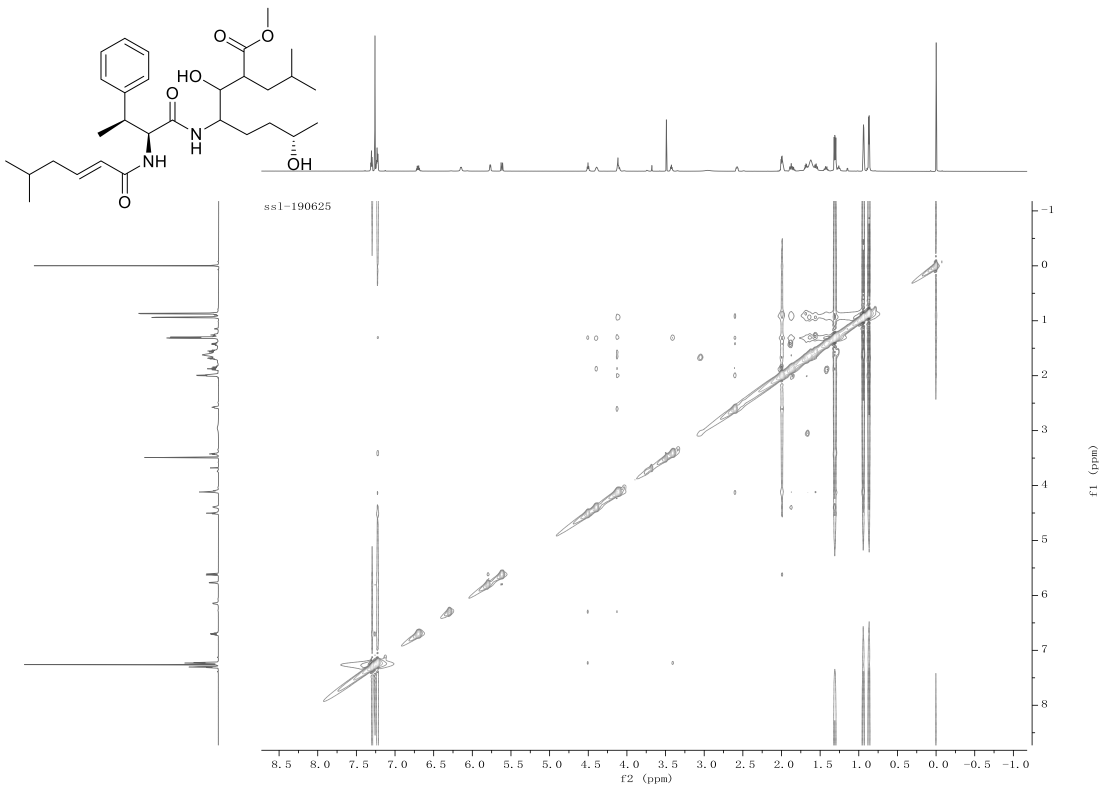

Figure S30. ROESY spectrum (800 MHz) of mintaimycin B (**2**) in $CDCl_3$.

Table S1. ^{1}H NMR data of mintaimycin B (**2**) and the known antibiotic M 9026 factor 3.

mintaimycin B (**2**)	antibiotic M 9026 factor 3
0.87, d (3H, *J* = 6.4)<br>0.87, d (3H, *J* = 6.4)	0.88, d (6H, *J* = 7)
0.93, d (3H, *J* = 6.4)<br>0.94, d (3H, *J* = 6.4)	0.91-0.94, 2d (6H, *J* = 7)
1.30, d (3H, *J* = 7.2)<br>1.31, d (3H, *J* = 7.2)	1.16, d (3H, *J* = 6.5)<br>1.31, d (3H, *J* = 7)
1.26, m (1H); 1.55, m (1H)<br>1.42, m (1H); 1.89, m (1H)<br>1.64, m (1H)<br>1.87, m (1H); 1.99, m (1H)<br>1.88, m (1H)<br>2.00, m (2H)	1.4-2.0, m (8H)<br>1.99, ddd (2H, *J* = 7-7-1)
2.58, m (1H)	2.49, ddd (1H, *J* = 2.5, 7-11)
	3.07, b (1H)*
3.43, m (1H)	3.34, dq (1H, *J* = 8)
3.49, s (3H)	3.68, s (3H)
	3.70, m (1H)*
4.10, overlap (1H)	3.75, dd (1H, *J* = 10)
4.11, overlap (1H)	3.88, b (1H)
4.39, m (1H)	4.01, dddd (*J* = 9, *J* = 2-11)
4.50, t (1H, *J* = 7.2)	4.54, dd (1H, *J* = 9)
5.62, d (1H, *J* = 15.2)	5.62, dt (1H, *J* = 15)
5.77, d (1H, *J* = 7.2)	6.16, d (1H)
6.15, d (1H, *J* = 8.0)	6.52, d (1H)
6.70, td (1H, *J* = 15.2, 7.2)	6.65, dt (1H)
7.23, d (1H, *J* = 7.2)<br>7.24, overlap (1H)<br>7.24, overlap (1H)<br>7.31, t (1H, *J* = 7.2)<br>7.31, t (1H, *J* = 7.2)	7.27, m (5H)

Note: ^{1}H NMR spectra were measured in $CDCl_3$ for mintaimycin B (**2**), and acetone-d_6 (speculated, unspecified in the patent) for antibiotic M 9026 factor 3. *: Hydroxy hydrogen signal.

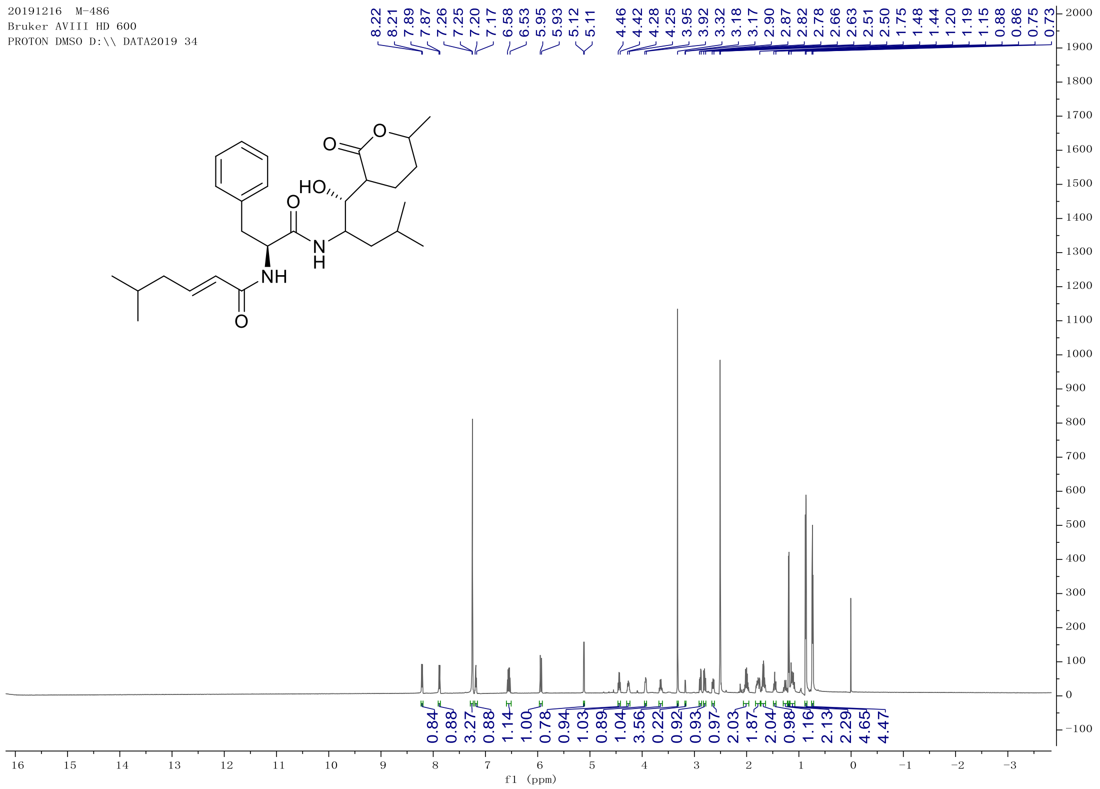

Figure S31. ^{1}H NMR spectrum (600 MHz) of mintaimycin A_2 (**3**) in DMSO-d_6.

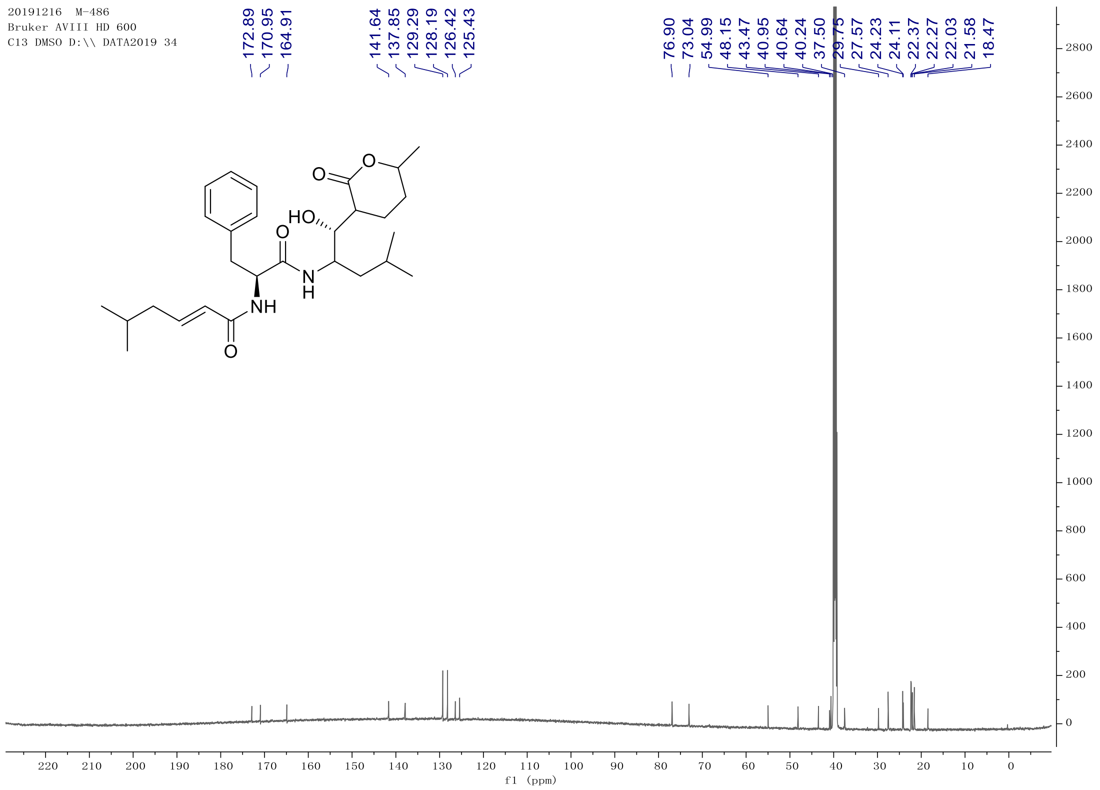

Figure S32. ^{13}C NMR spectrum (150 MHz) of mintaimycin A_2 (**3**) in DMSO-d_6.

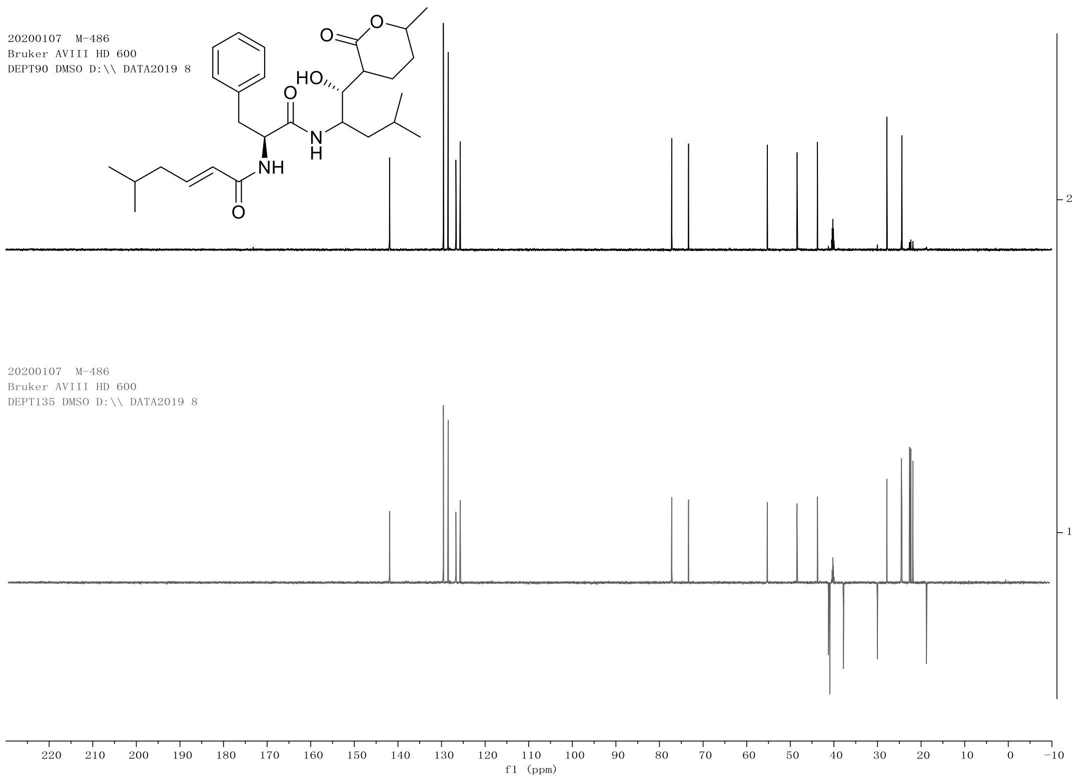

Figure S33. DEPT spectrum (150 MHz) of mintaimycin A_2 (**3**) in DMSO-d_6.

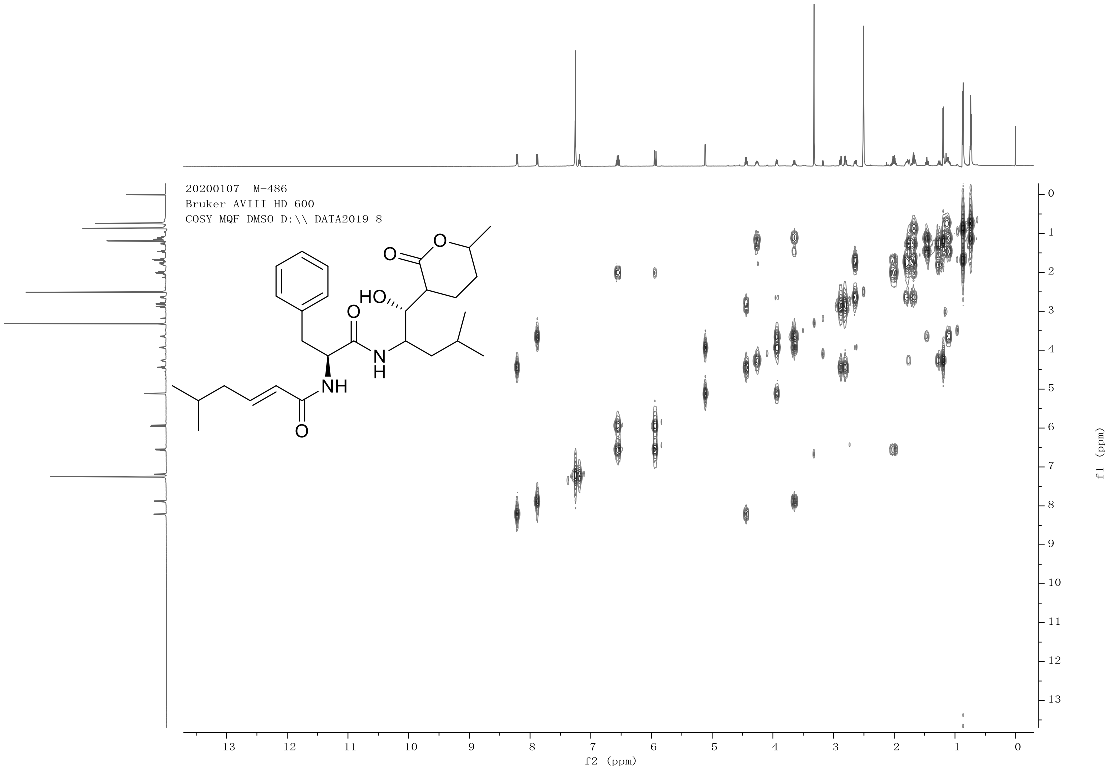

Figure S34. ^{1}H-^{1}H COSY spectrum (600 MHz) of mintaimycin A_2 (**3**) in DMSO-d_6.

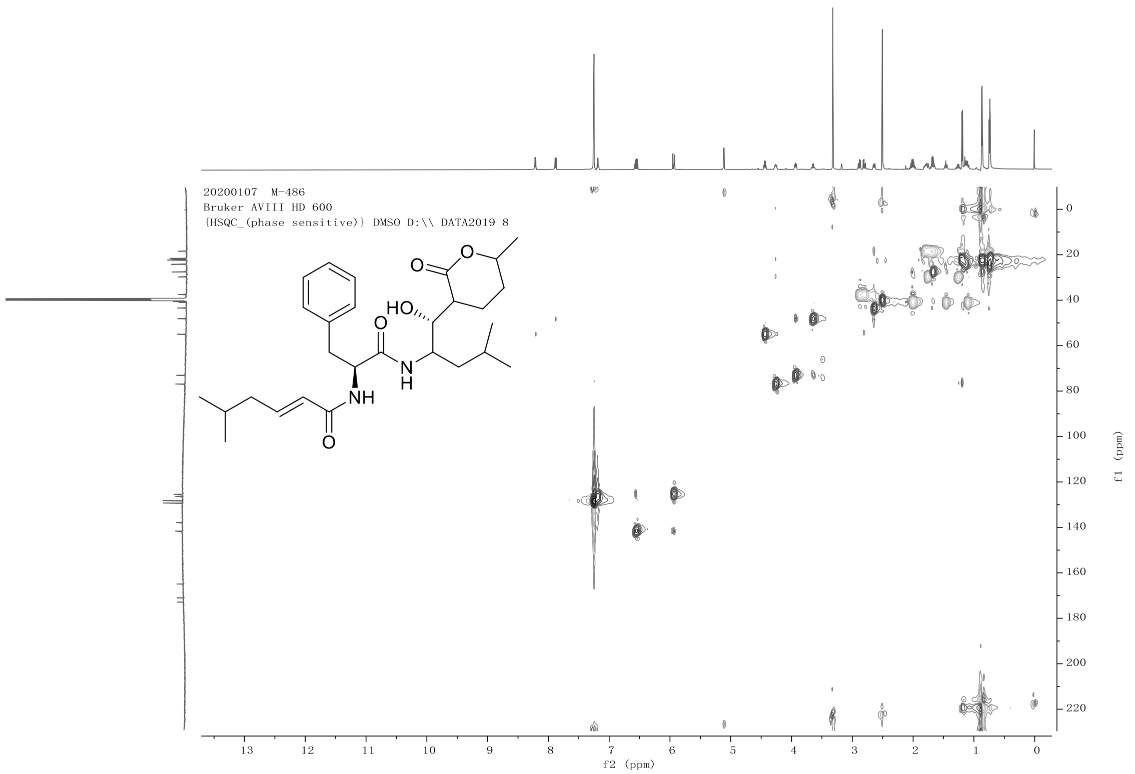

Figure S35. HSQC spectrum (150 MHz) of mintaimycin A_2 (**3**) in DMSO-d_6.

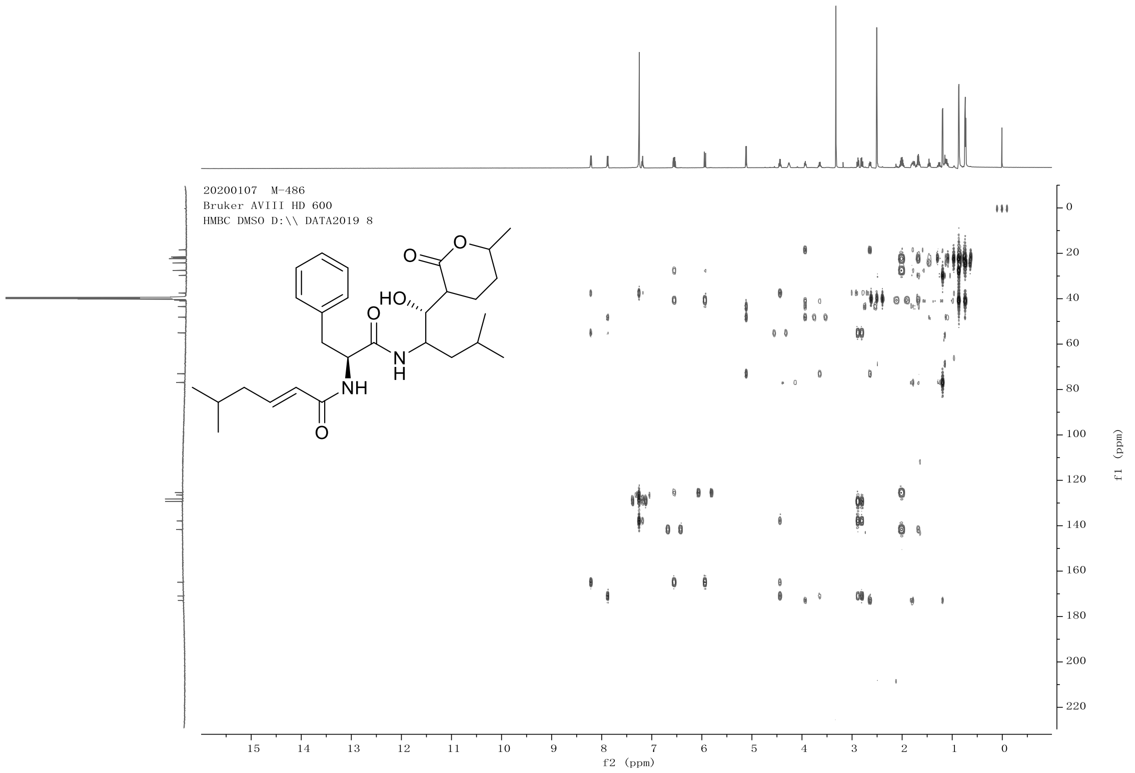

Figure S36. HMBC spectrum (150 MHz) of mintaimycin A_2 (**3**) in DMSO-d_6.

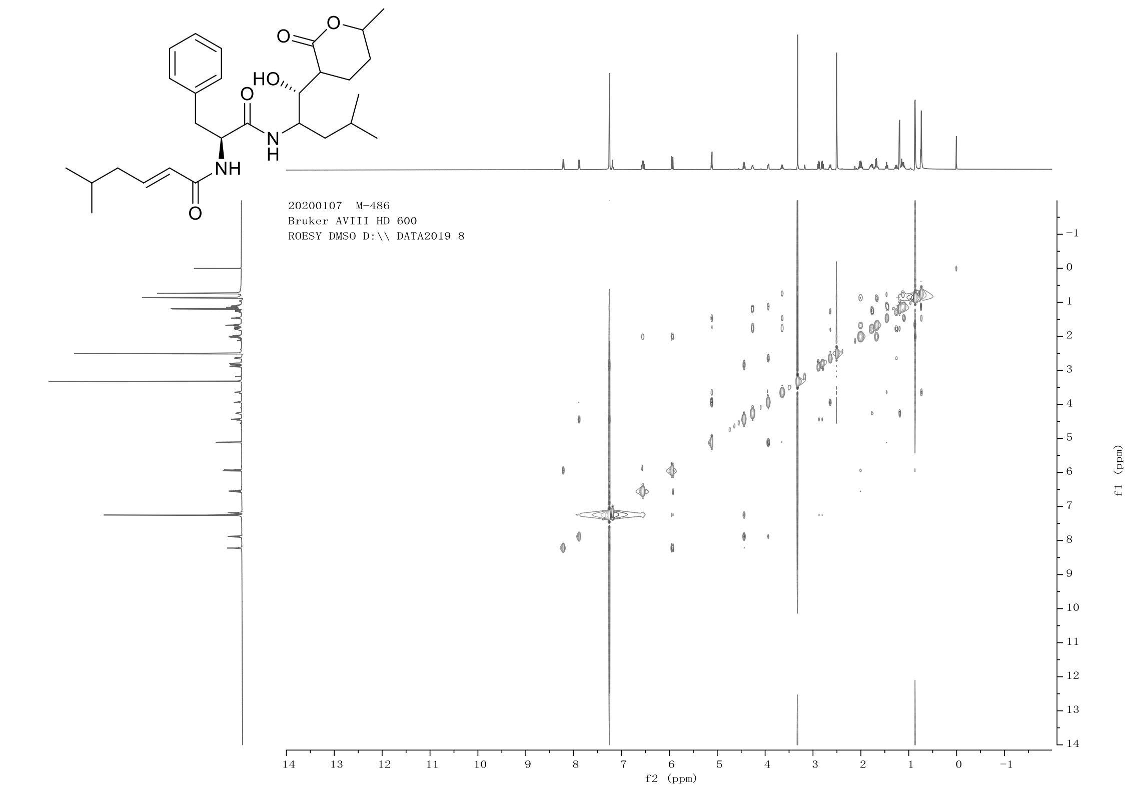

Figure S37. ROESY spectrum (600 MHz) of mintaimycin A_2 (**3**) in DMSO-d_6.

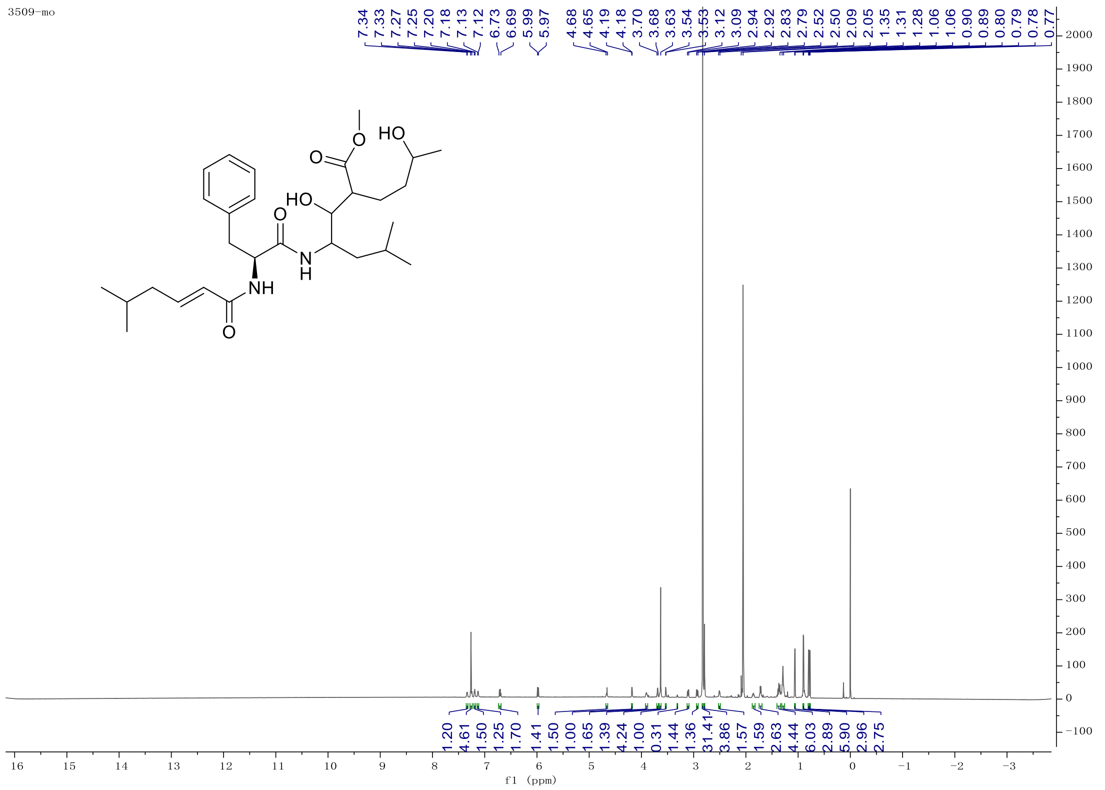

Figure S38. ^{1}H NMR spectrum (800 MHz) of mintaimycin A_3 (**4**) in acetone-d_6.

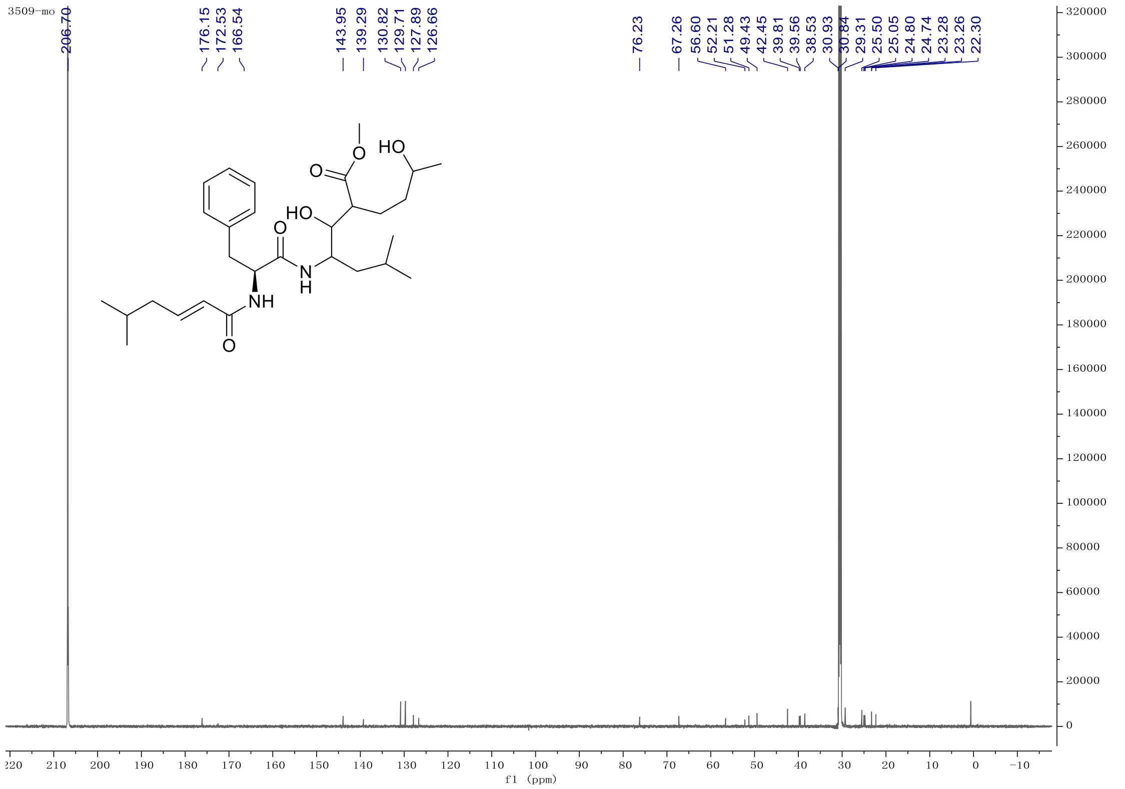

Figure S39. ^{13}C NMR spectrum (200 MHz) of mintaimycin A$_3$ (**4**) in acetone-d_6.

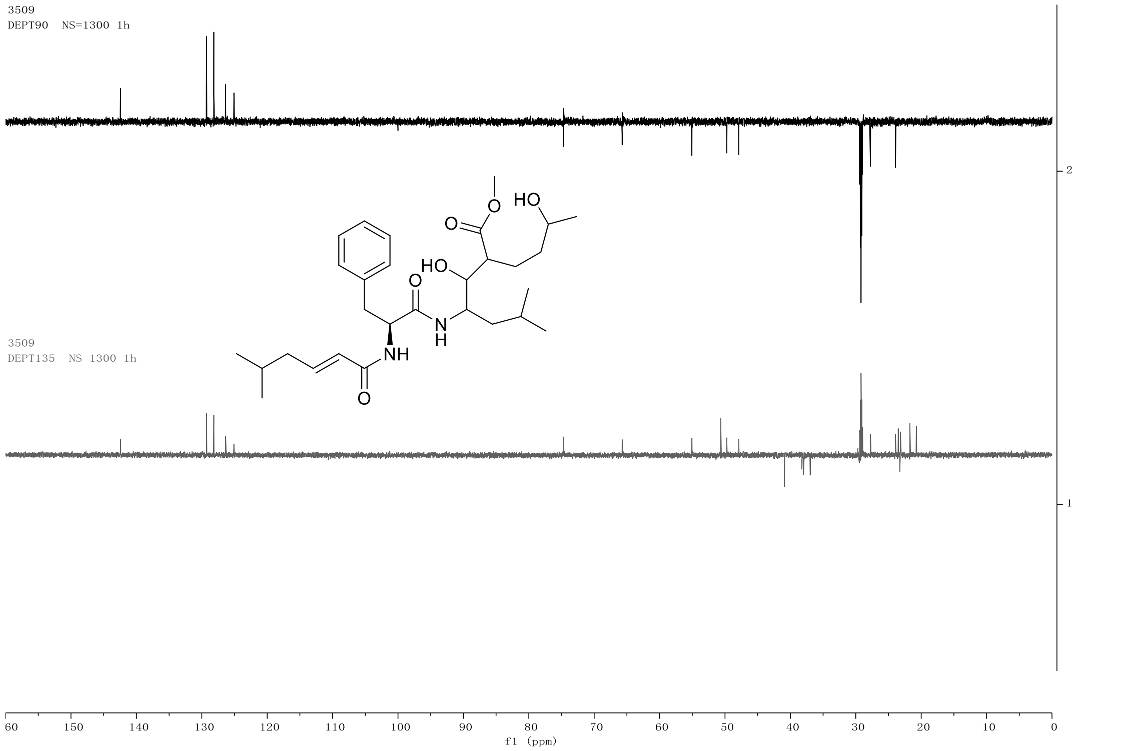

Figure S40. DEPT spectrum (175 MHz) of mintaimycin A$_3$ (**4**) in acetone-d_6.

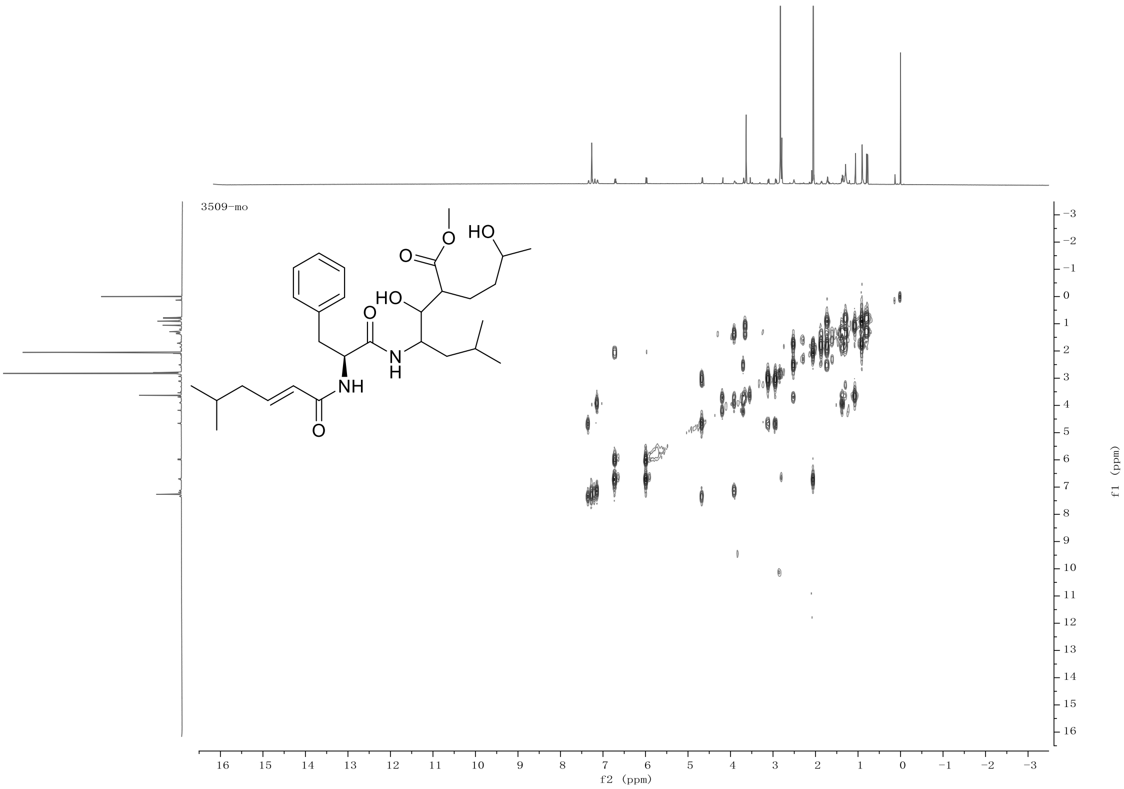

Figure S41. ^{1}H-^{1}H COSY spectrum (800 MHz) of mintaimycin A_3 (**4**) in acetone-d_6.

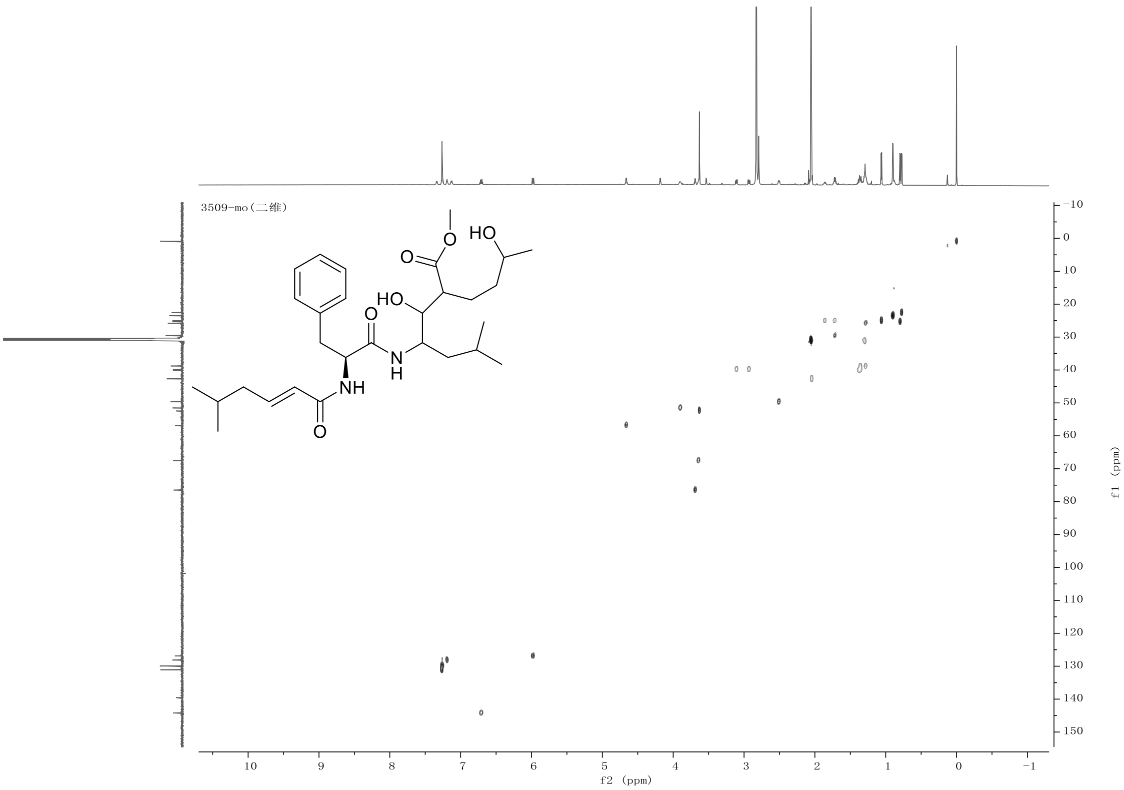

Figure S42. HSQC spectrum (200 MHz) of mintaimycin A_3 (**4**) in acetone-d_6.

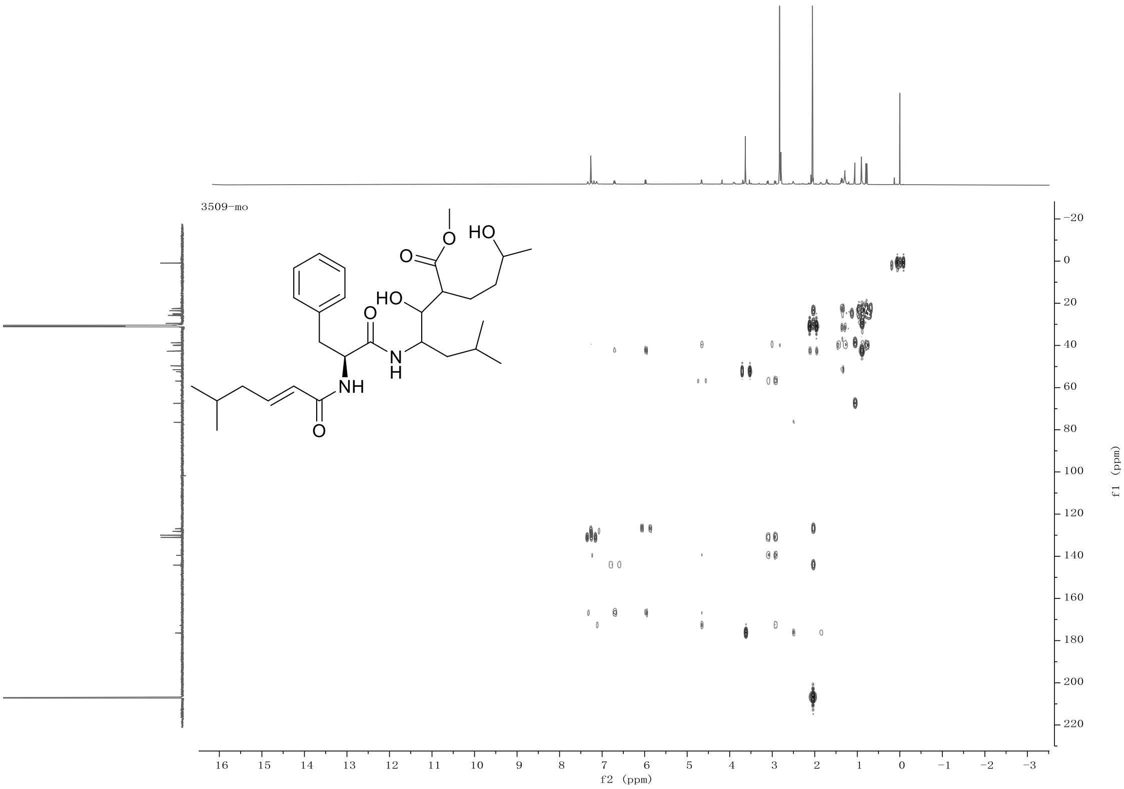

Figure S43. HMBC spectrum (200 MHz) of mintaimycin A_3 (**4**) in acetone-d_6.

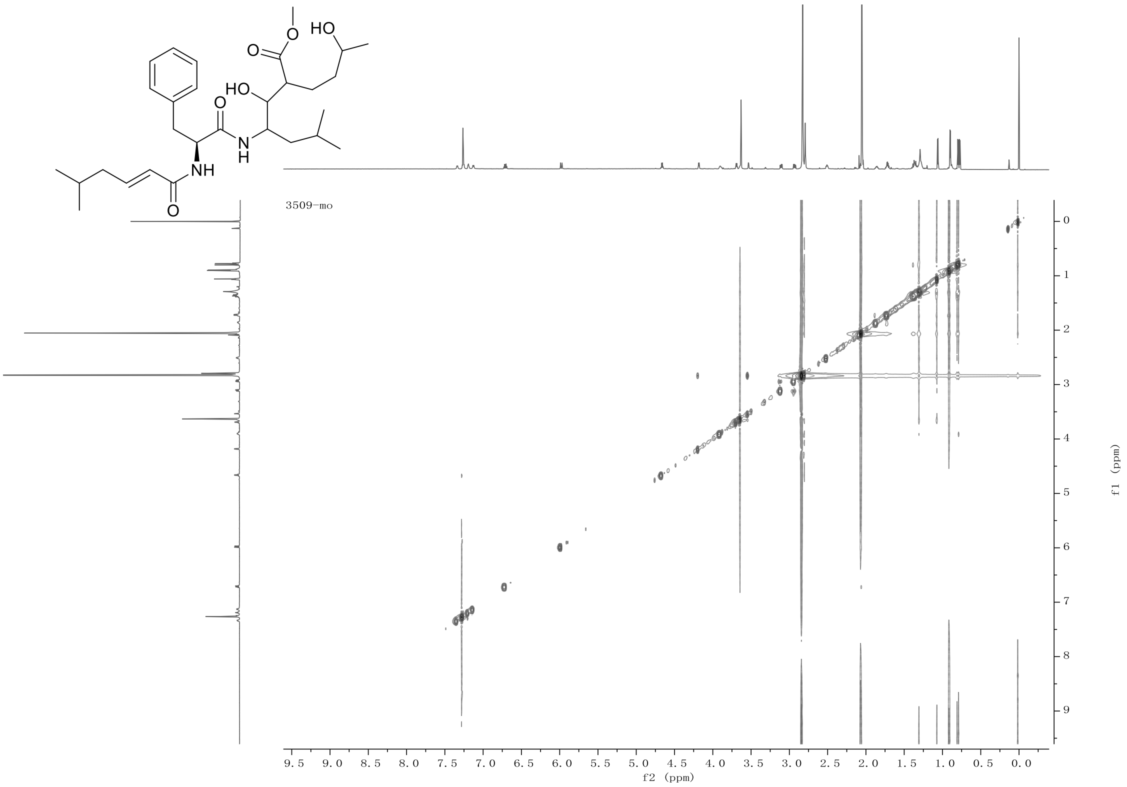

Figure S44. ROESY spectrum (800 MHz) of mintaimycin A_3 (**4**) in acetone-d_6.

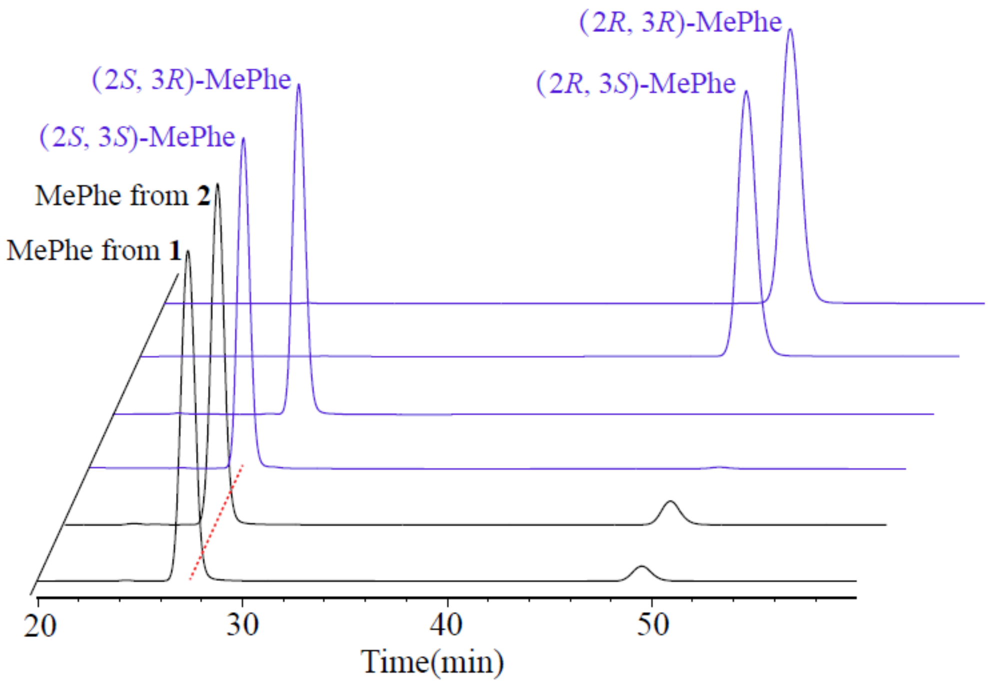

Figure S45. SIM mode (*m/z* 474.1) of mintaimycin A_1 (**1**) and mintaimycin B (**2**) by Marfey's method. LC-MS using a ZORBAX SB-C_3 column (Agilent, 5 µm, 150 × 4.6 mm). The reaction products were analyzed by LC-MS starting with 35% MeCN/65% H_2O (H_2O contained 0.1% formic acid, v/v) followed by a gradient elution profile to 40% MeCN/60% H_2O (H_2O contained 0.1% formic acid, v/v) over 60 min at a flow rate of 1.0 mL/min, monitoring at 340 nm.

Retention times for the amino acid standards:

27.5 min for (2*S*, 3*S*)-*β*-MePhe,

28.9 min for (2*S*, 3*R*)-*β*-MePhe,

49.5 min for (2*R*, 3*S*)-*β*-MePhe,

50.5 min for (2*R*, 3*R*)-*β*-MePhe.

The hydrolysates of both **1** and **2** revealed a peak at 27.4 min.

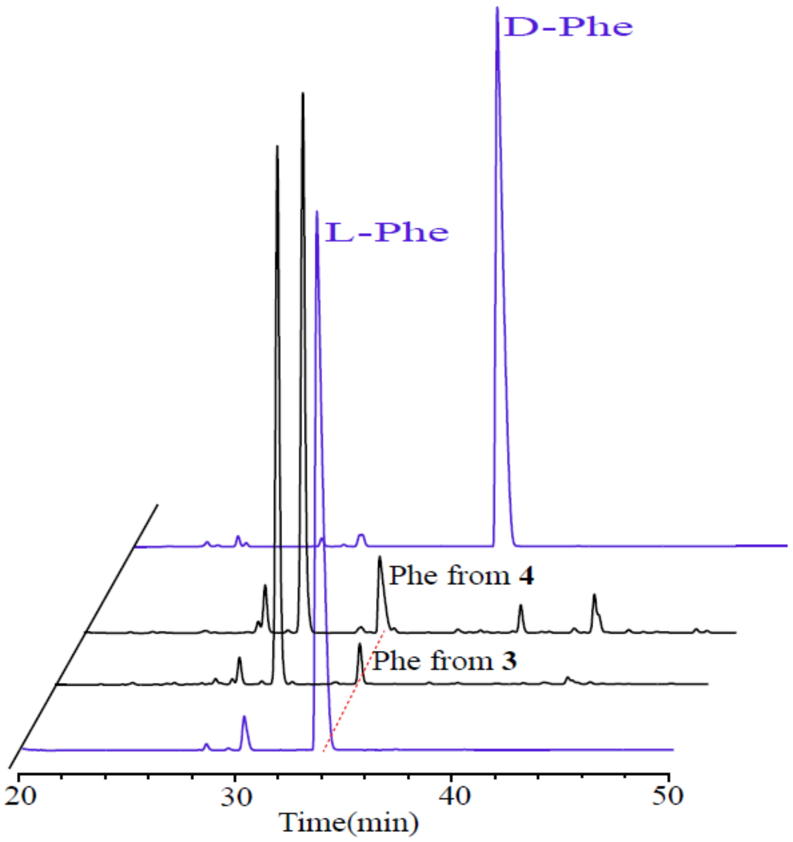

Figure S46. HPLC analysis of mintaimycin $A_{3\text{-}4}$ (**3-4**) by Marfey's method. The reaction products were analyzed by HPLC starting with 10% MeCN/90% H_2O (H_2O contained 0.1% HAc, v/v) followed by a gradient elution profile to 80% MeCN/20% H_2O (H_2O contained 0.1% HAc, v/v) over 60 min at a flow rate of 1.0 mL/min, monitoring at 340 nm.

Retention times for the amino acid standards:

33.6 min for L-Phe,

36.6 min for D-Phe.

The hydrolysates of **3** and **4** revealed a peak at 33.9 and 33.6 min, respectively.

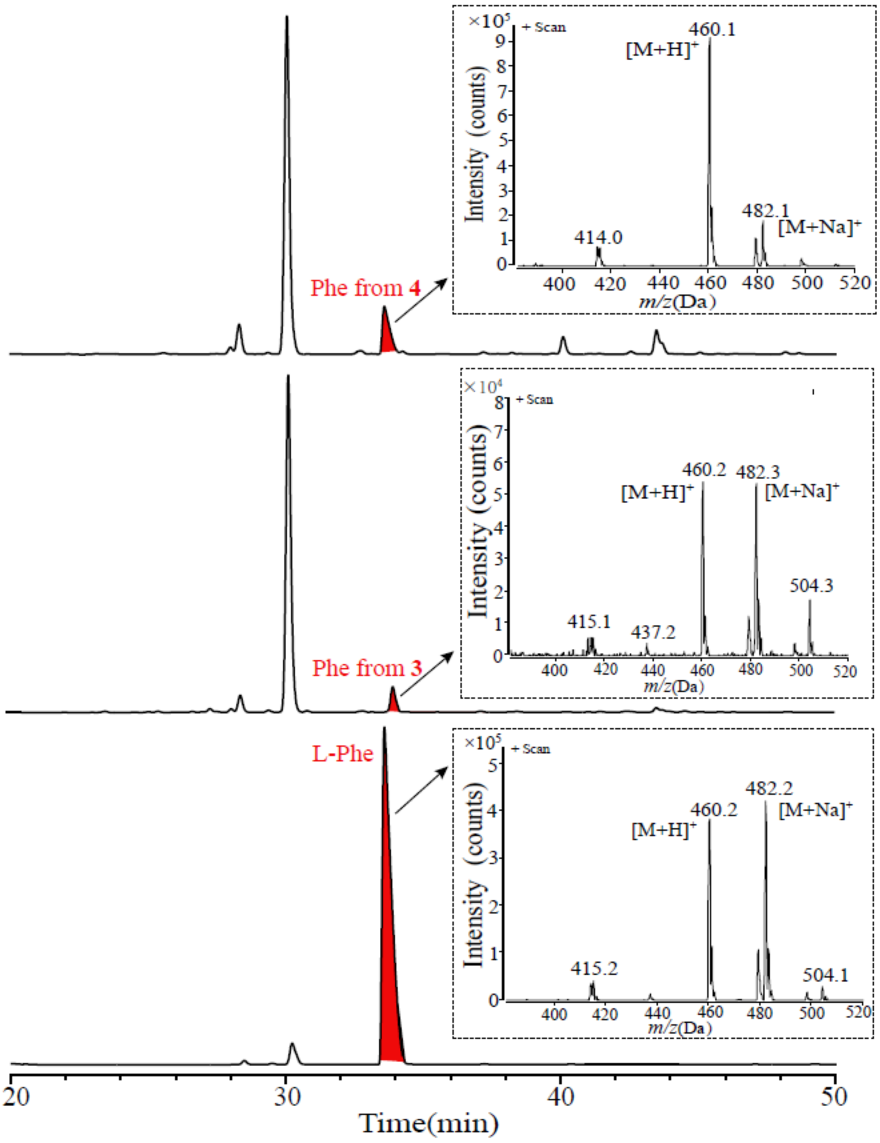

Figure S47. LC-MS analysis of mintaimycin $A_{3\text{-}4}$ (**3-4**) by Marfey's method.

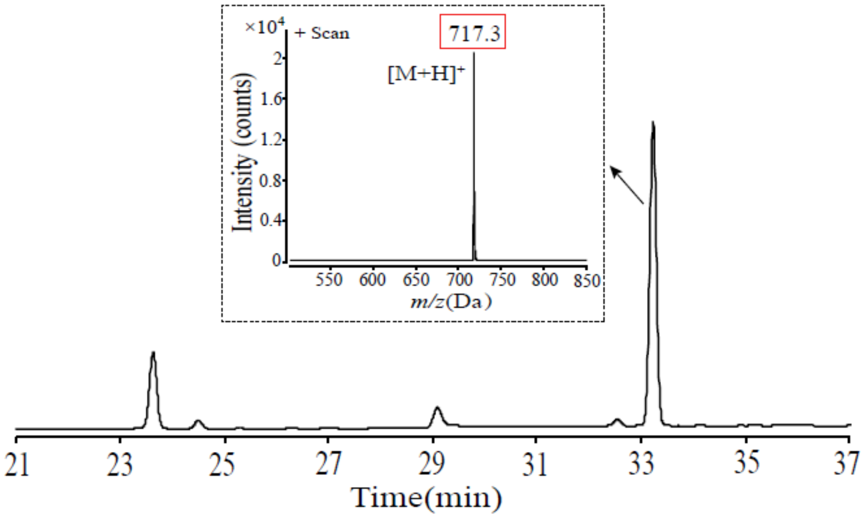

Figure S48. LC-MS spectrum of (*R*)-MTPA ester of mintaimycin A_1 (**1**).

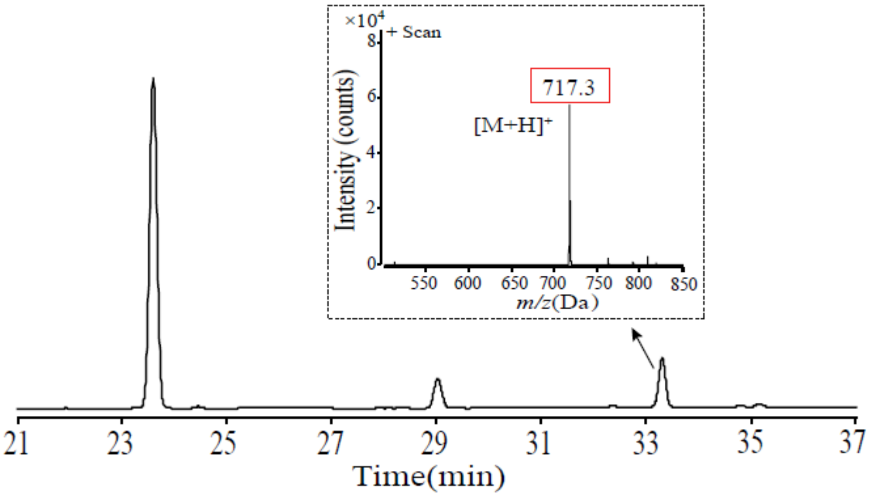

Figure S49. LC-MS spectrum of (*S*)-MTPA ester of mintaimycin A_1 (**1**).

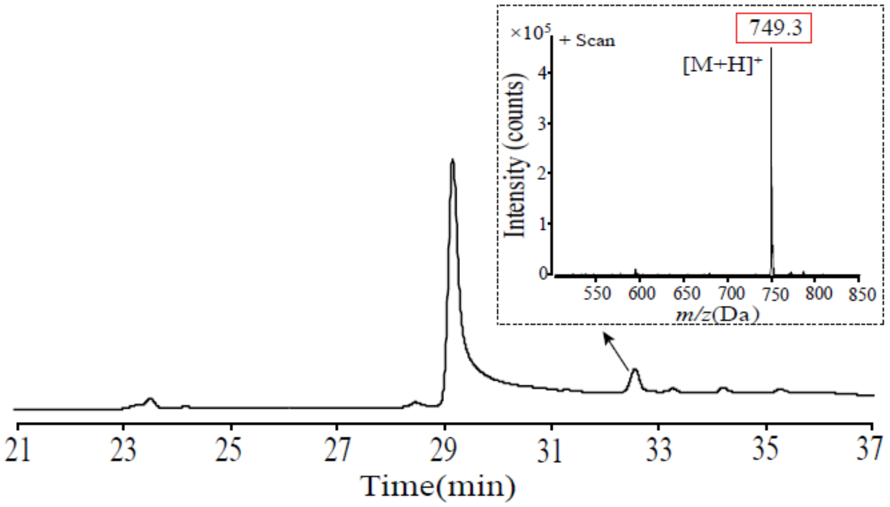

Figure S50. LC-MS spectrum of (*R*)-MTPA ester of mintaimycin B (**2**).

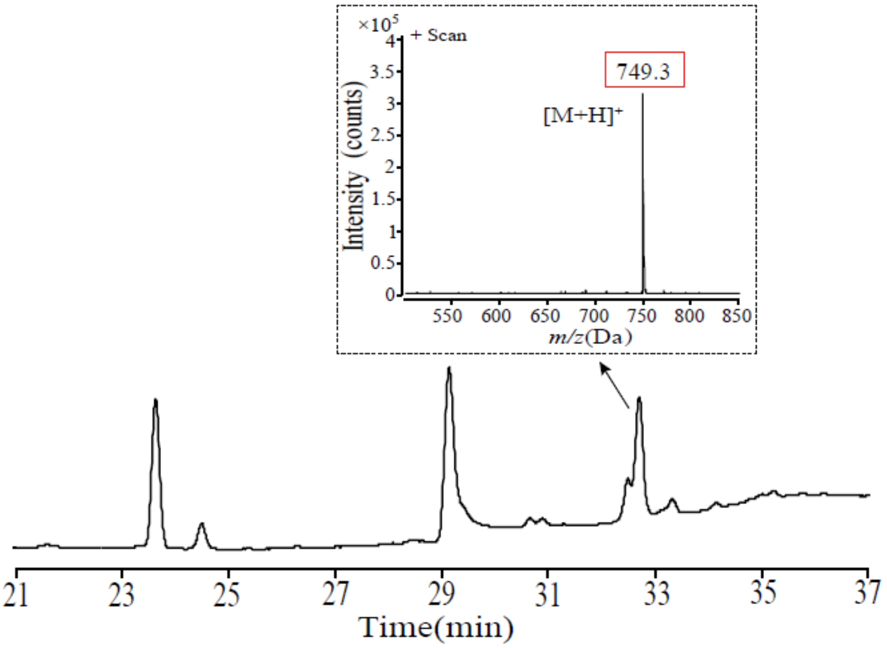

Figure S51. LC-MS spectrum of (*S*)-MTPA ester of mintaimycin B (**2**).

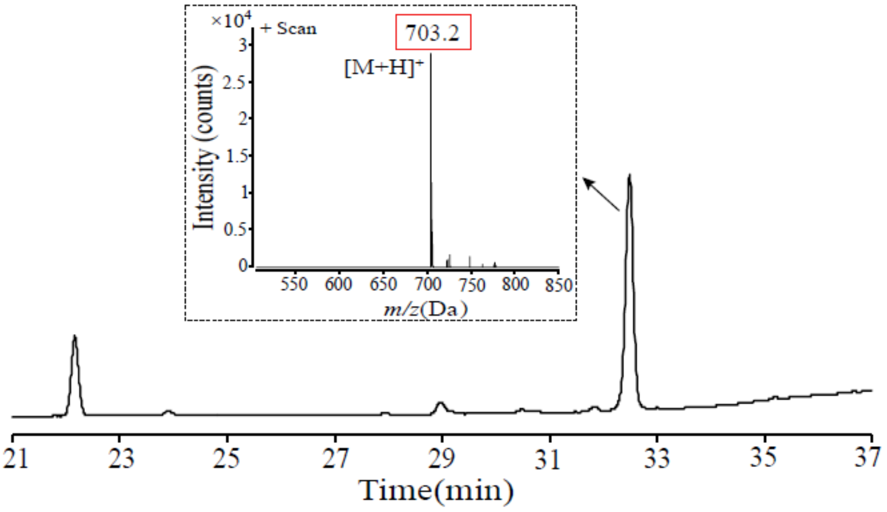

Figure S52. LC-MS spectrum of (*R*)-MTPA ester of mintaimycin A_2 (**3**).

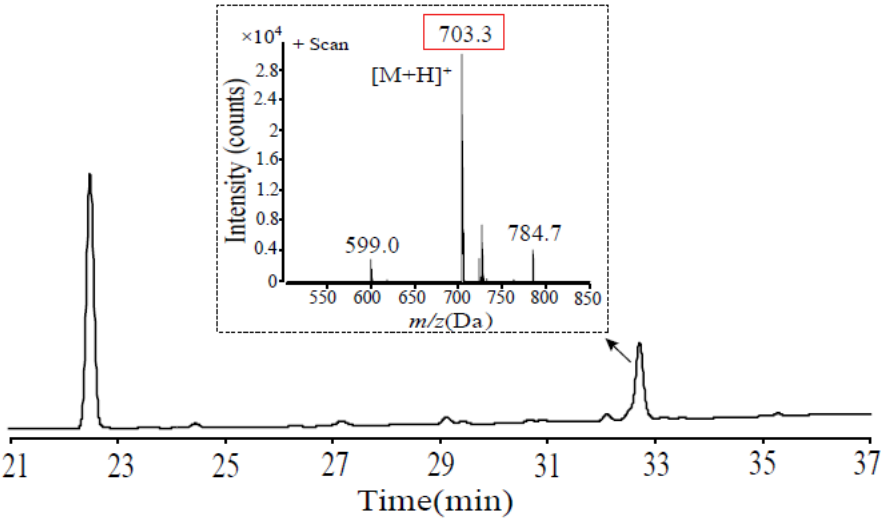

Figure S53. LC-MS spectrum of (*S*)-MTPA ester of mintaimycin A_2 (**3**).

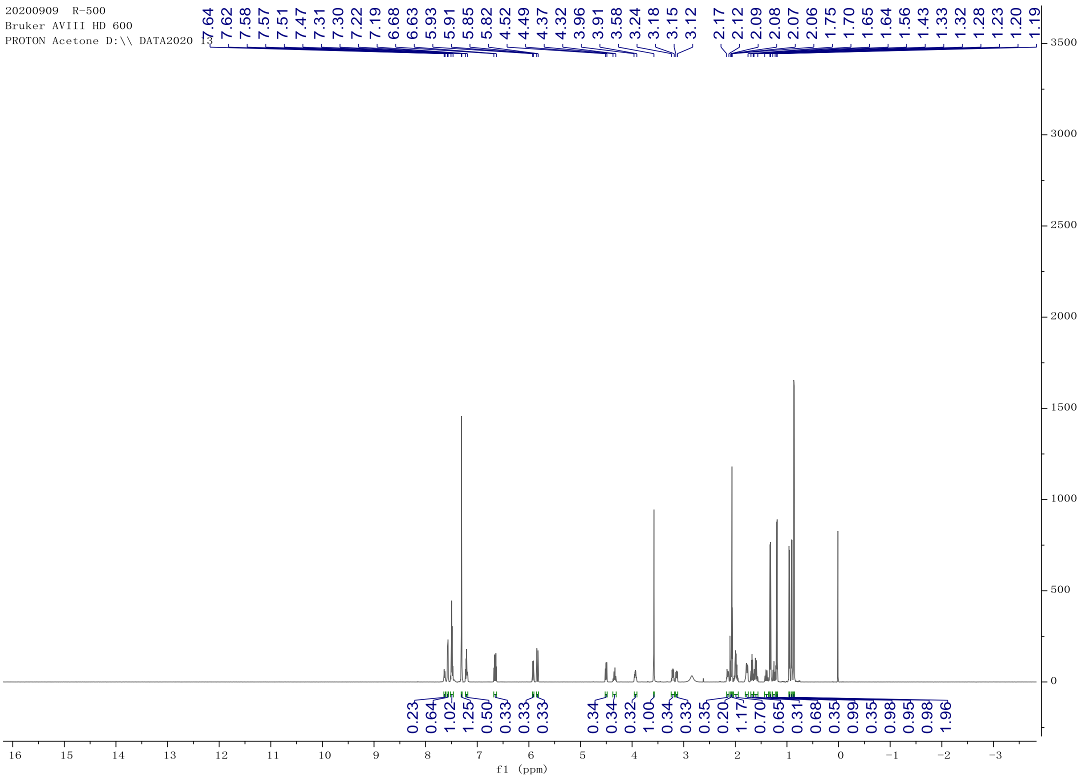

Figure S54. ^{1}H NMR spectrum (600 MHz) of (*R*)-MTPA ester of mintaimycin A_1 (**1**) in acetone-d_6.

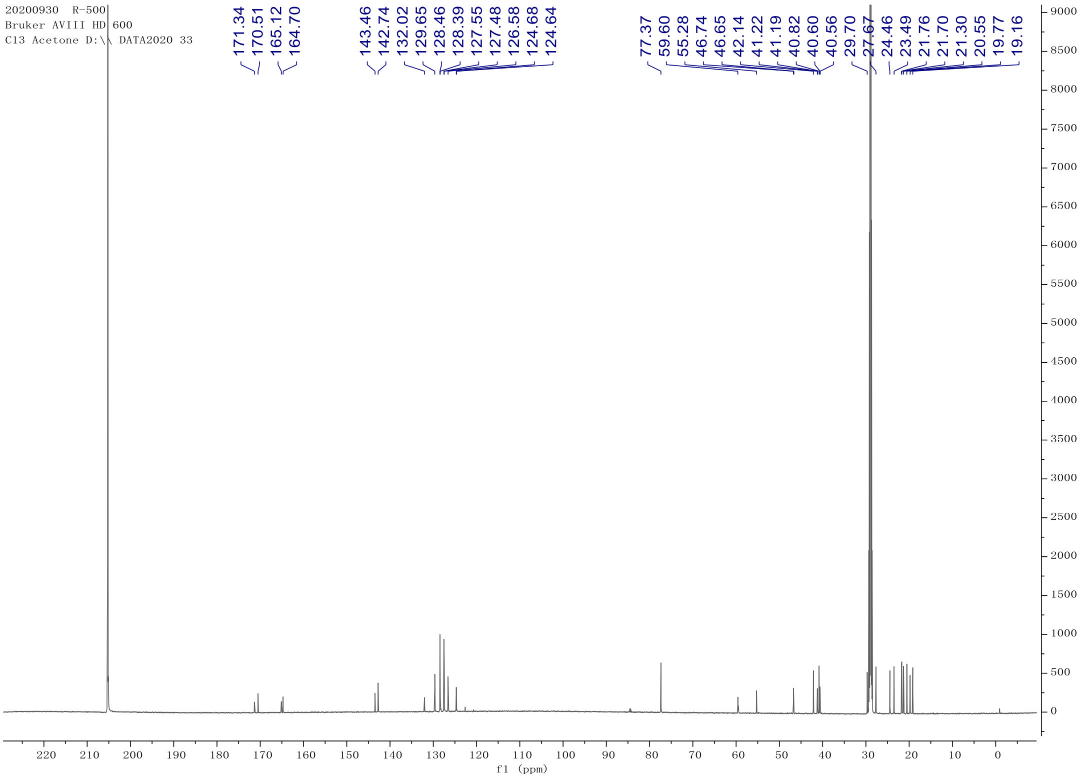

Figure S55. ^{13}C NMR spectrum (150 MHz) of (*R*)-MTPA ester of mintaimycin A_1 (**1**) in acetone-d_6.

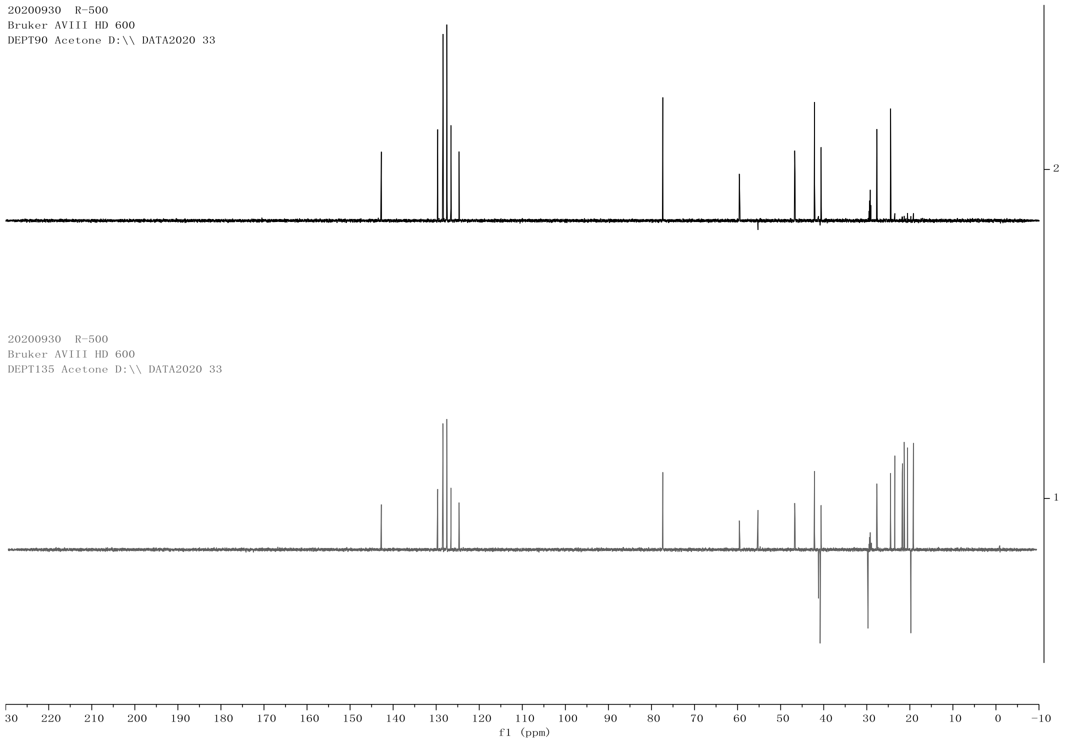

Figure S56. DEPT spectrum (150 MHz) of (*R*)-MTPA ester of mintaimycin A_1 (**1**) in acetone-d_6.

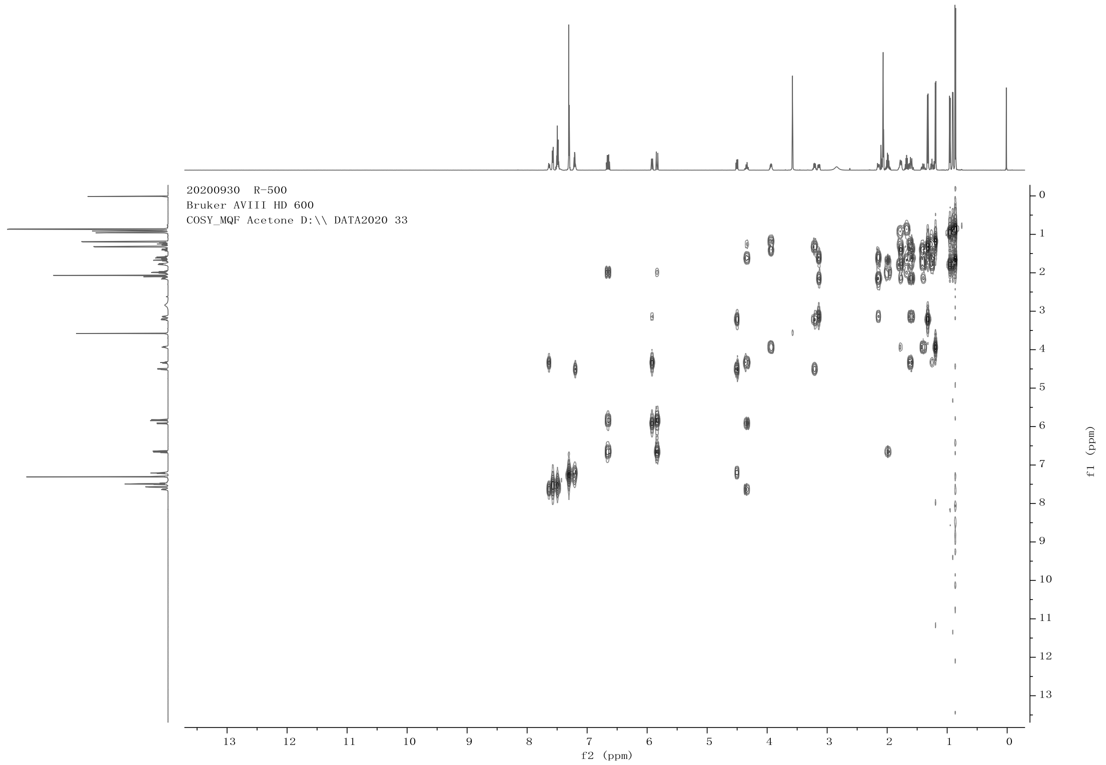

Figure S57. ^{1}H-^{1}H COSY spectrum (600 MHz) of (*R*)-MTPA ester of mintaimycin A_1 (**1**) in acetone-d_6.

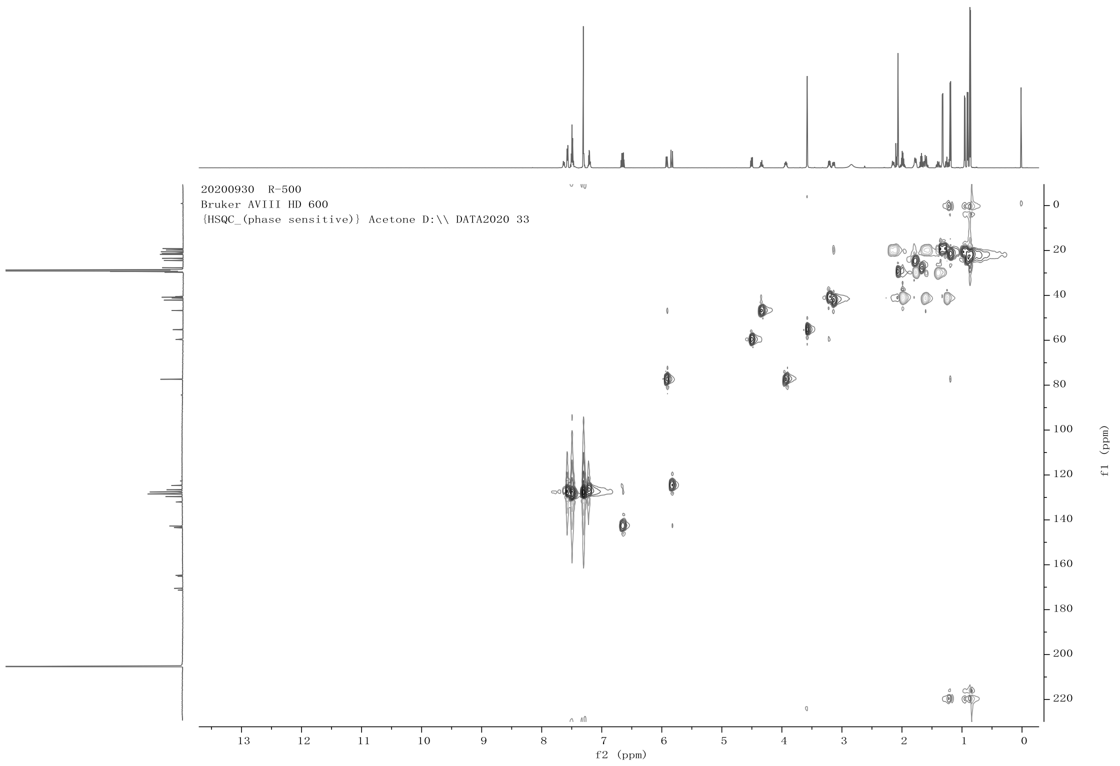

Figure S58. HSQC spectrum (150 MHz) of (*R*)-MTPA ester of mintaimycin A_1 (**1**) in acetone-d_6.

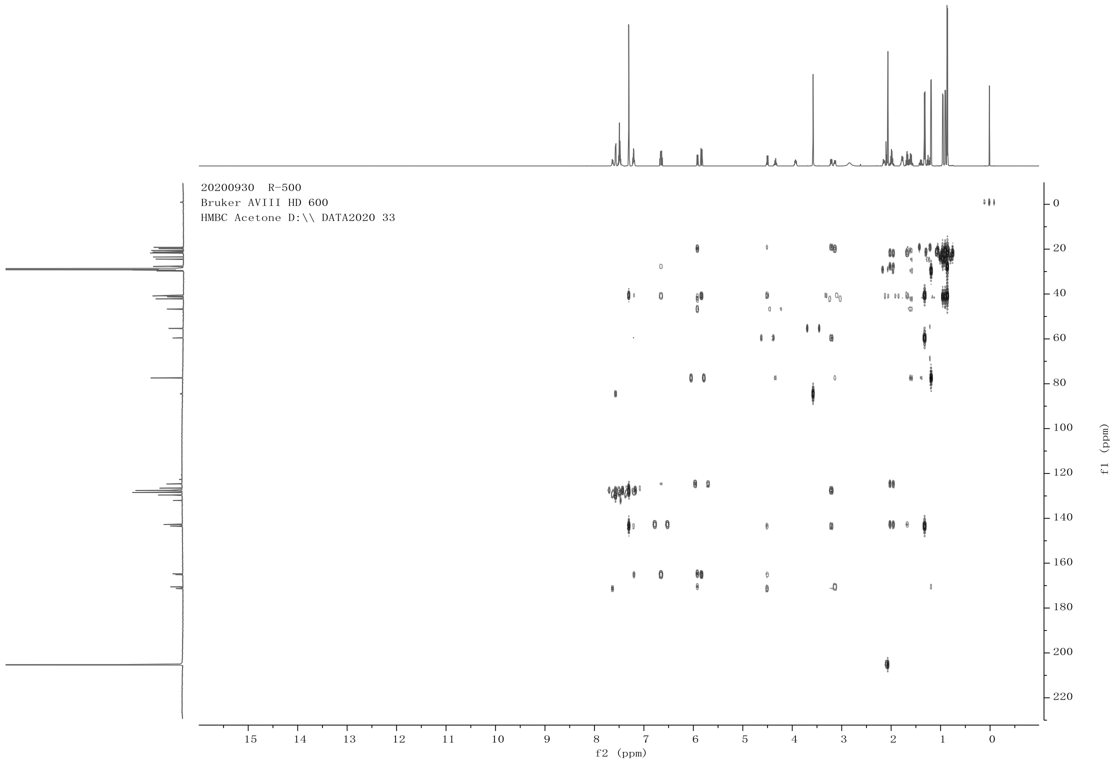

Figure S59. HMBC spectrum (150 MHz) of (*R*)-MTPA ester of mintaimycin A_1 (**1**) in acetone-d_6.

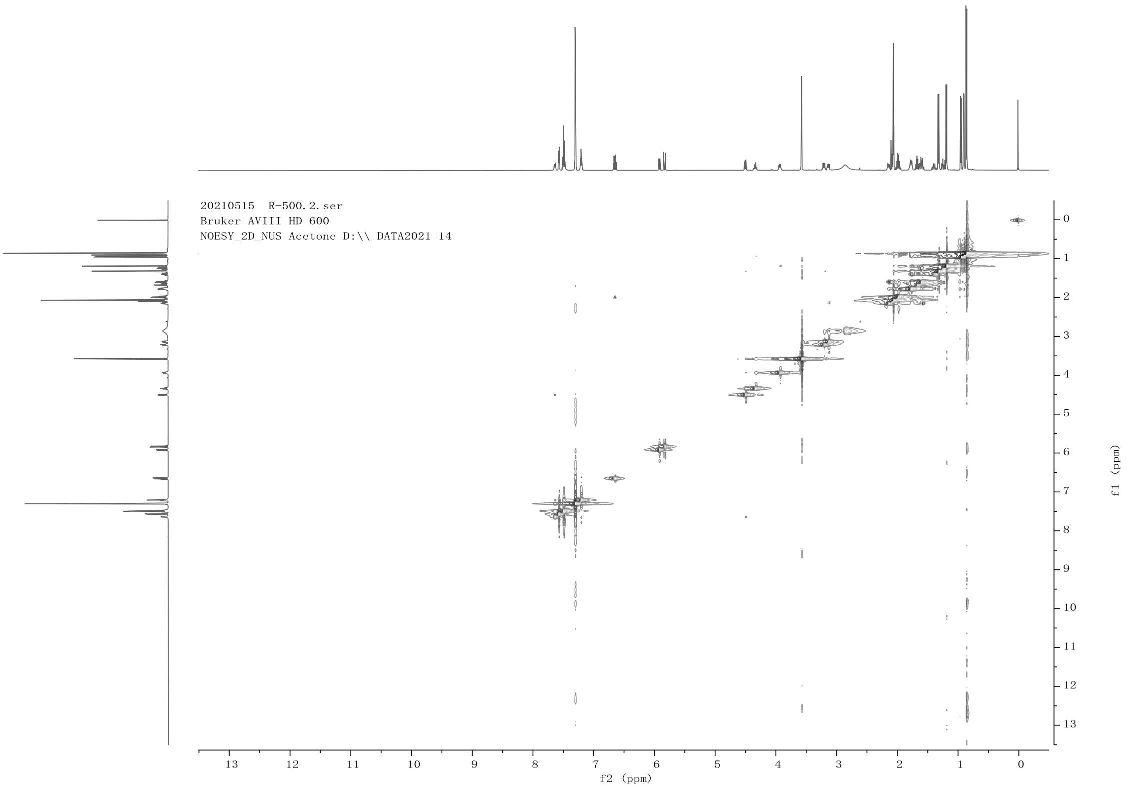

Figure S60. NOESY spectrum (600 MHz) of (*R*)-MTPA ester of mintaimycin A_1 (**1**) in acetone-d_6.

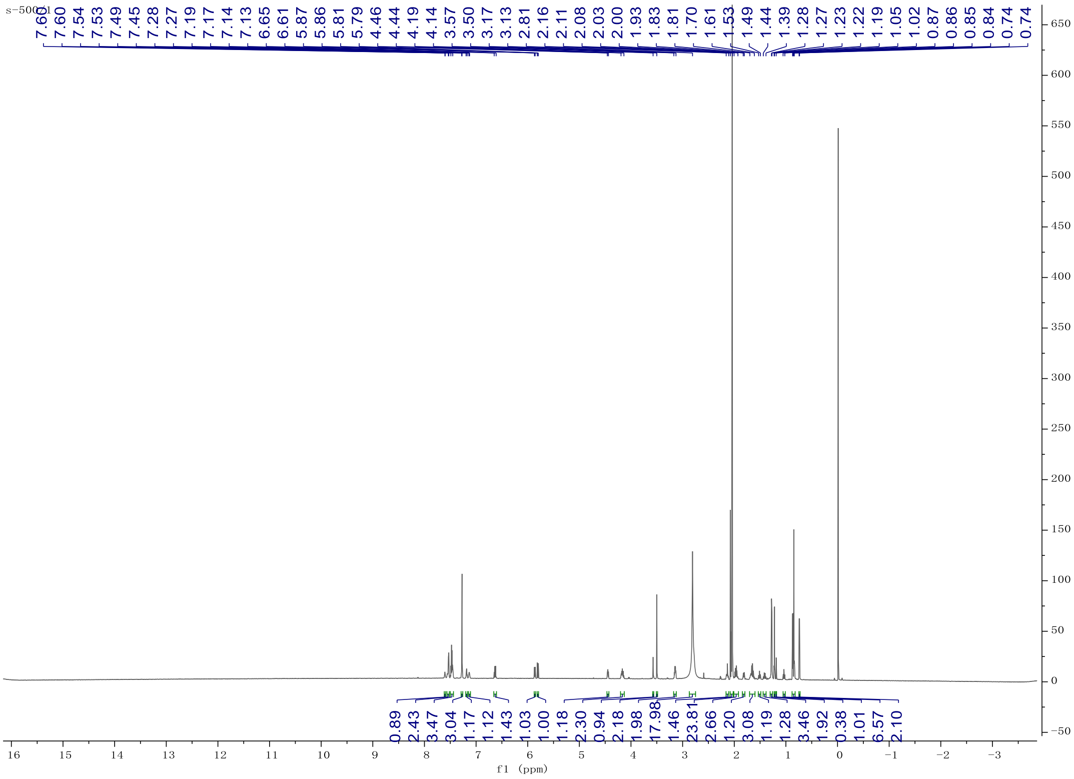

Figure S61. ^{1}H NMR Spectrum (800 MHz) of (*S*)-MTPA ester of mintaimycin A_1 (**1**) in acetone-d_6.

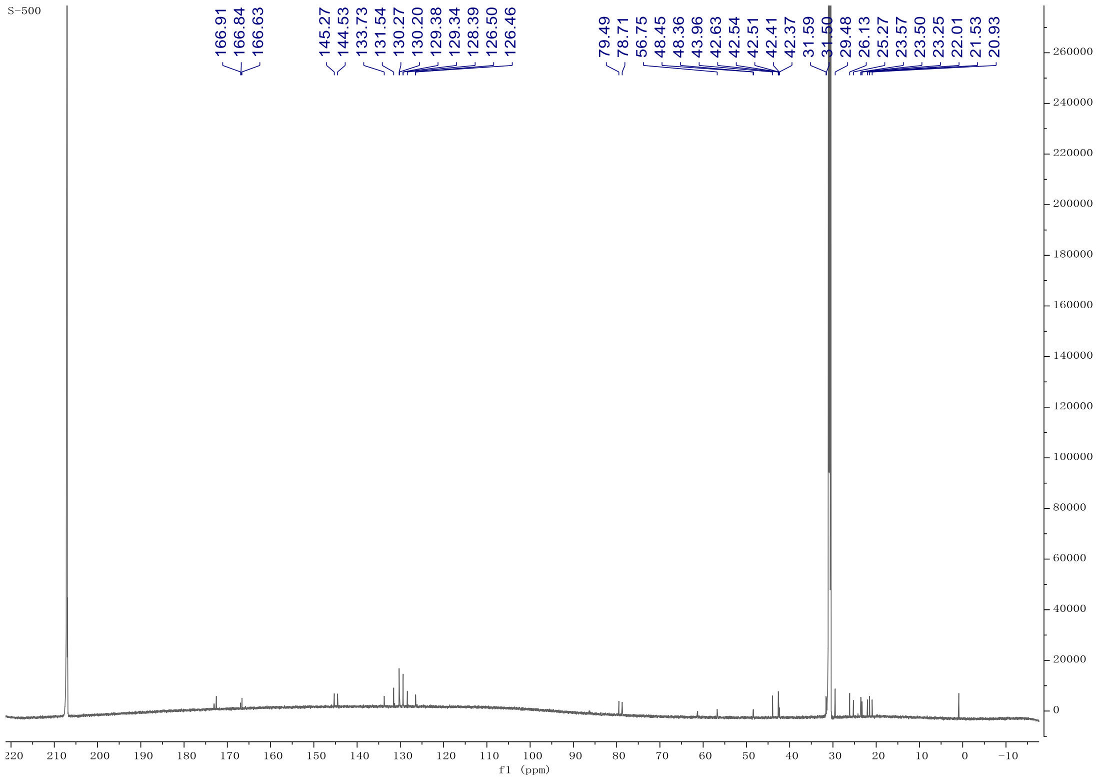

Figure S62. ^{13}C NMR spectrum (200 MHz) of (*S*)-MTPA ester of mintaimycin A_1 (**1**) in acetone-d_6.

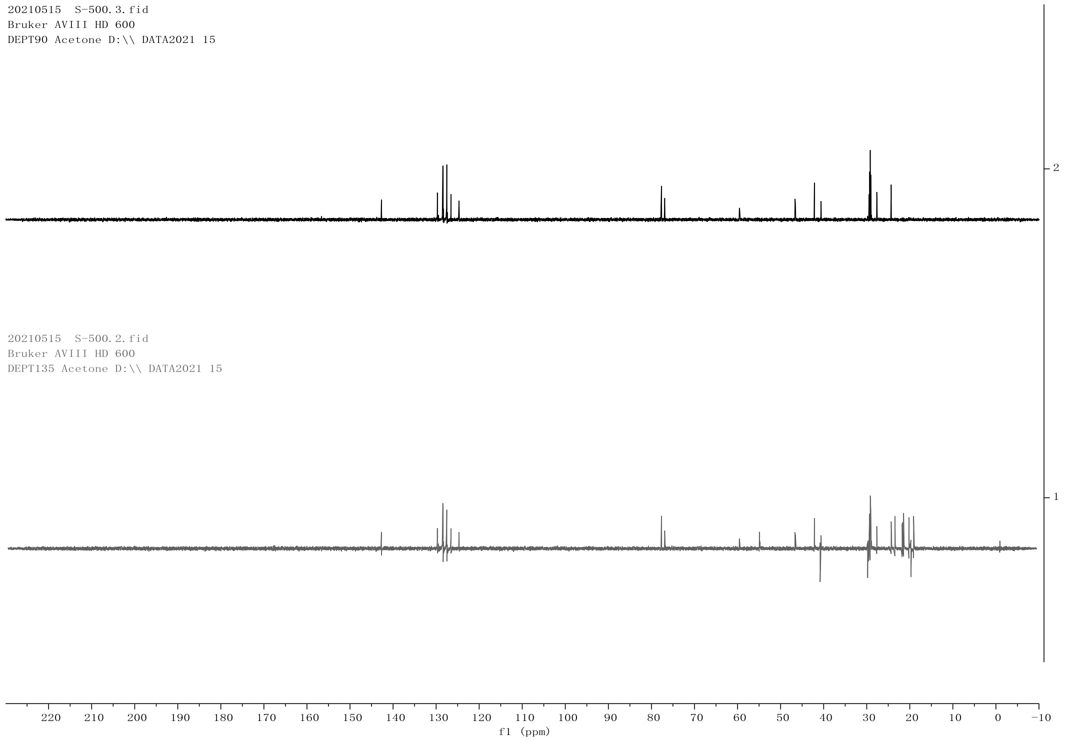

Figure S63. DEPT spectrum (150 MHz) of (*S*)-MTPA ester of mintaimycin A_1 (**1**) in acetone-d_6.

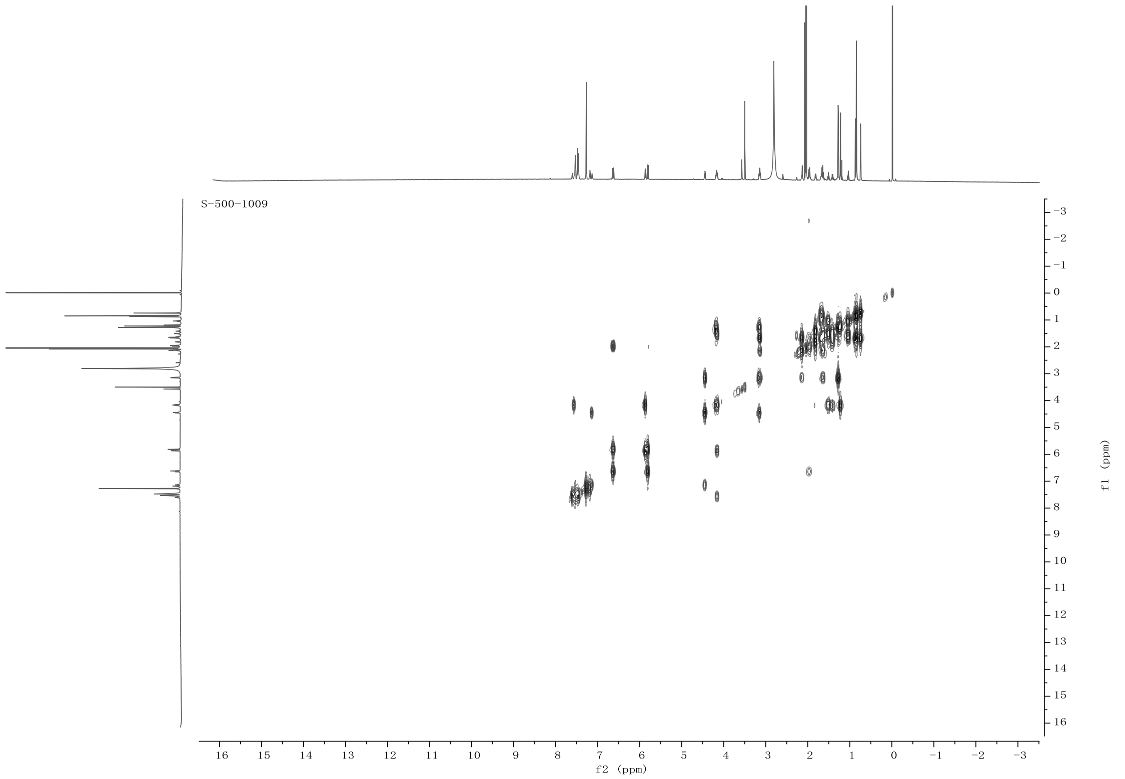

Figure S64. ^{1}H-^{1}H COSY spectrum (800 MHz) of (*S*)-MTPA ester of mintaimycin A$_1$ (**1**) in acetone-d_6.

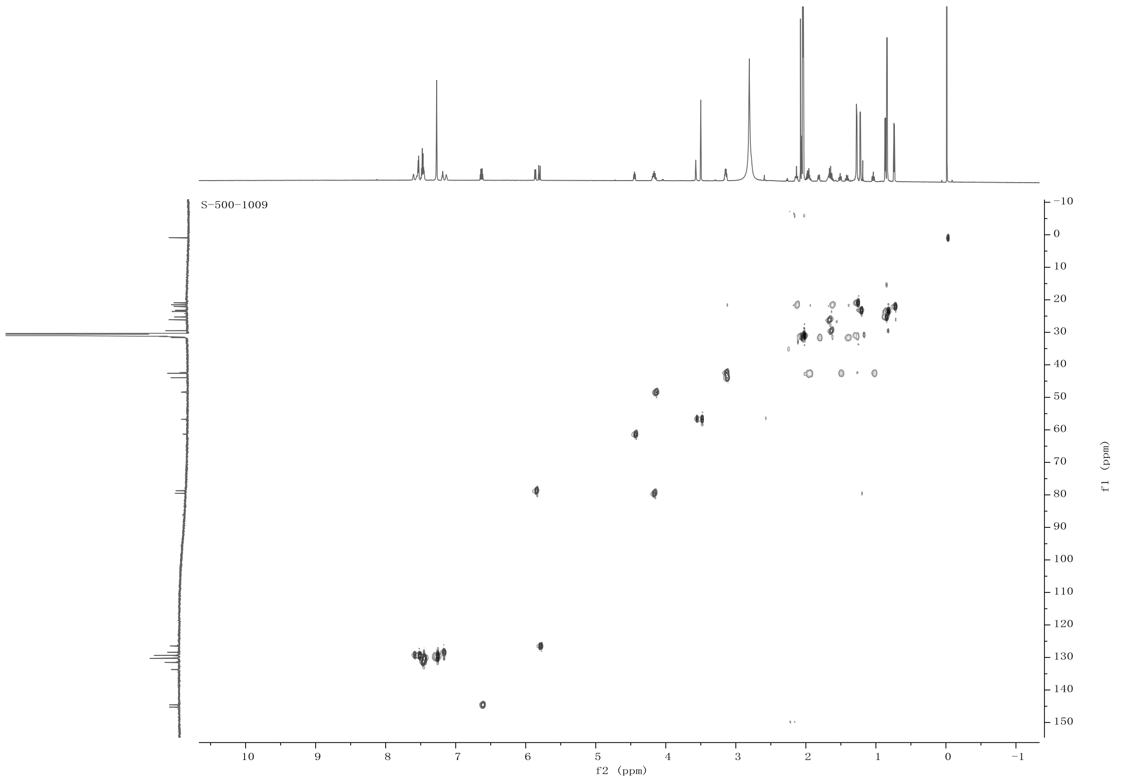

Figure S65. HSQC spectrum (200 MHz) of (*S*)-MTPA ester of mintaimycin A$_1$ (**1**) in acetone-d_6.

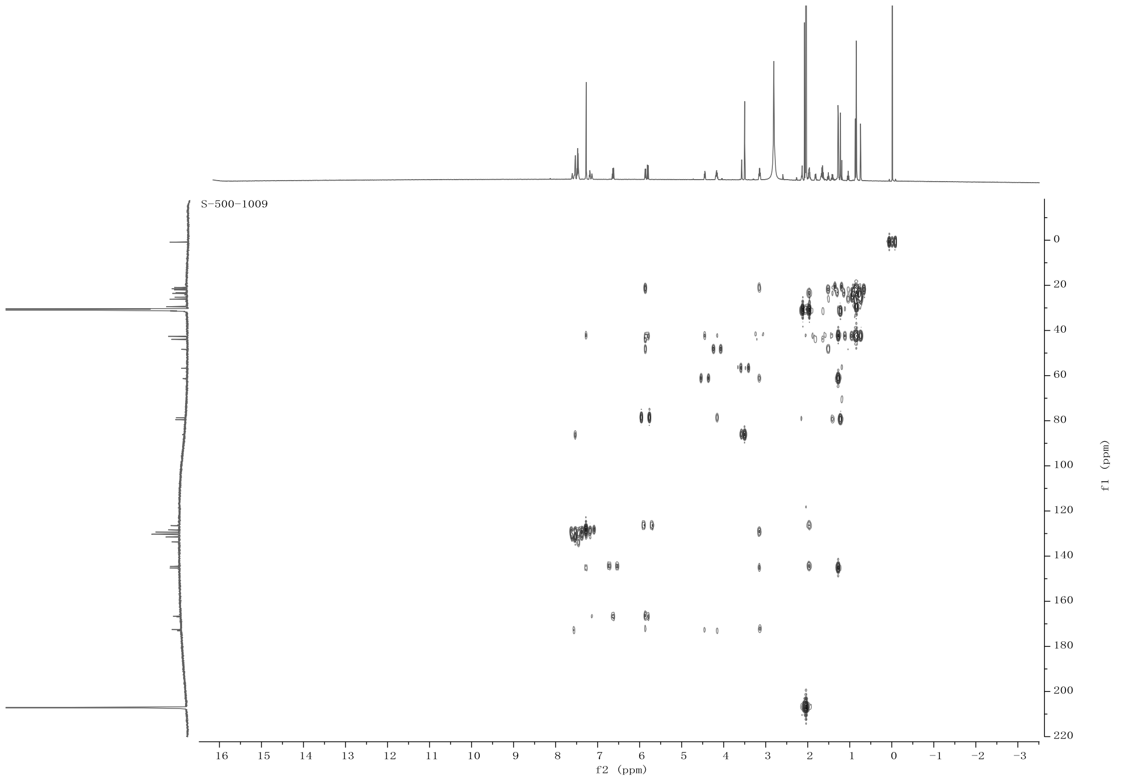

Figure S66. HMBC spectrum (200 MHz) of (*S*)-MTPA ester of mintaimycin A_1 (**1**) in acetone-d_6.

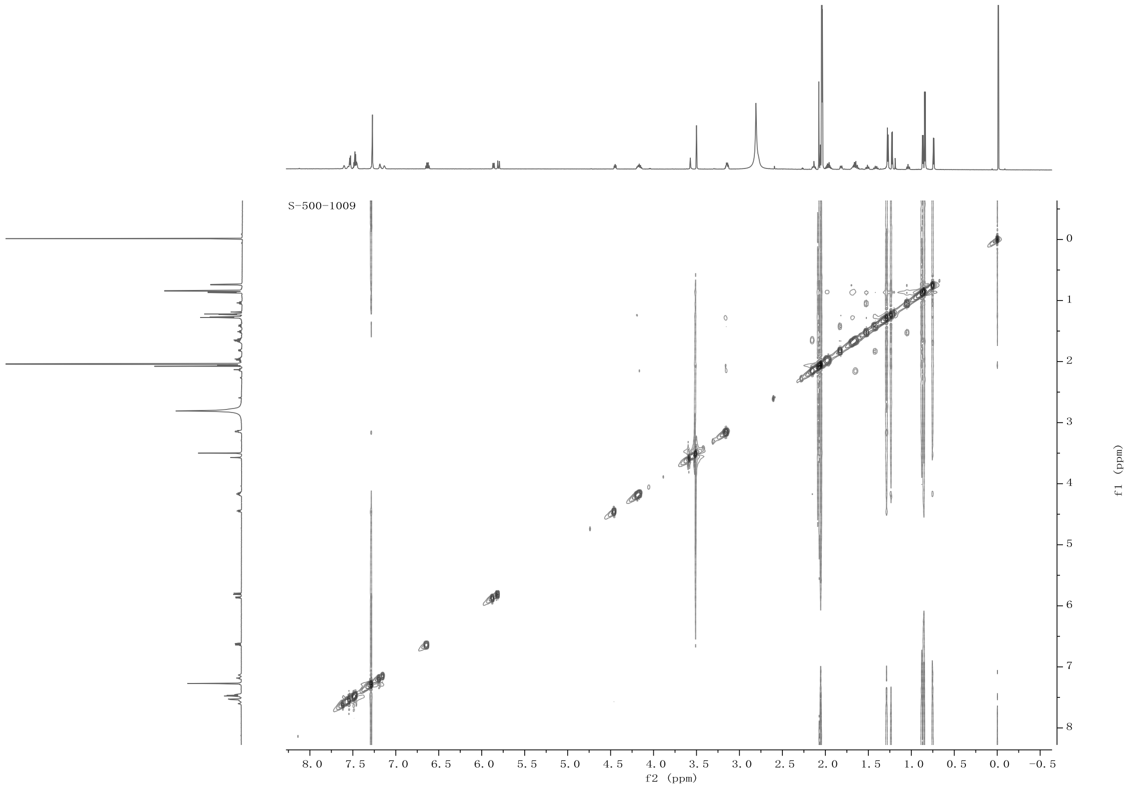

Figure S67. ROESY spectrum (800 MHz) of (*S*)-MTPA ester of mintaimycin A_1 (**1**) in acetone-d_6.

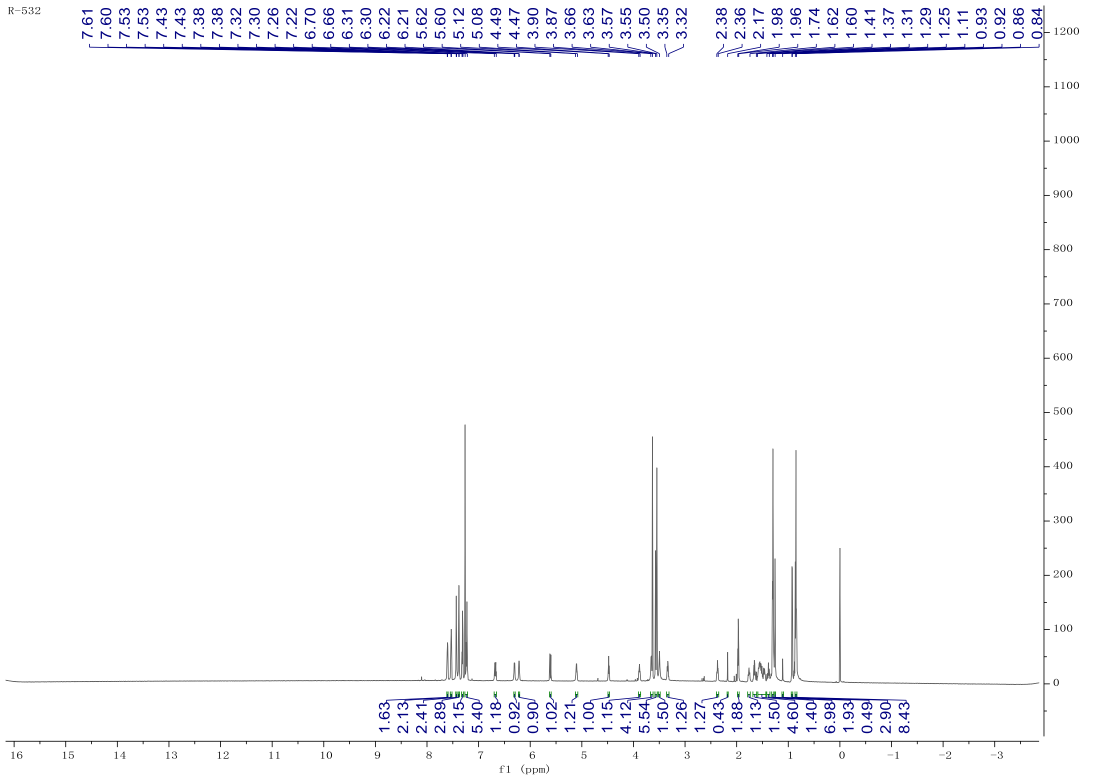

Figure S68. ^{1}H NMR spectrum (800 MHz) of (*R*)-MTPA ester of mintaimycin B (**2**) in $CDCl_3$.

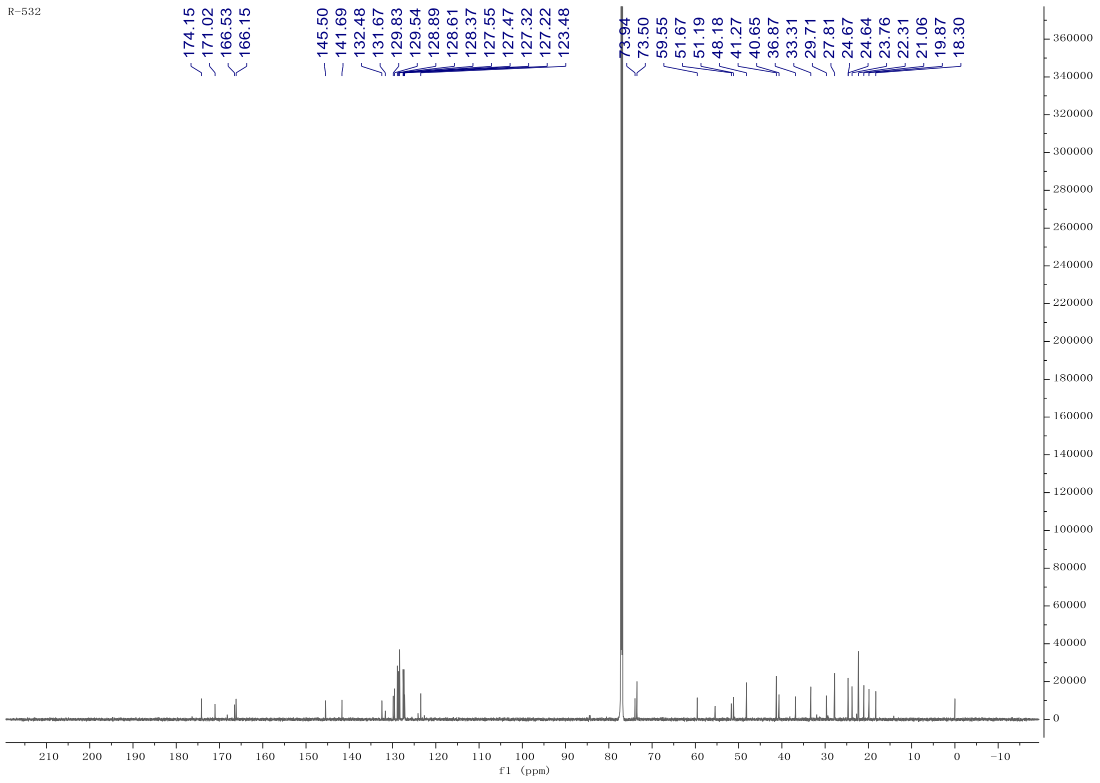

Figure S69. ^{13}C NMR spectrum (200 MHz) of (*R*)-MTPA ester of mintaimycin B (**2**) in $CDCl_3$.

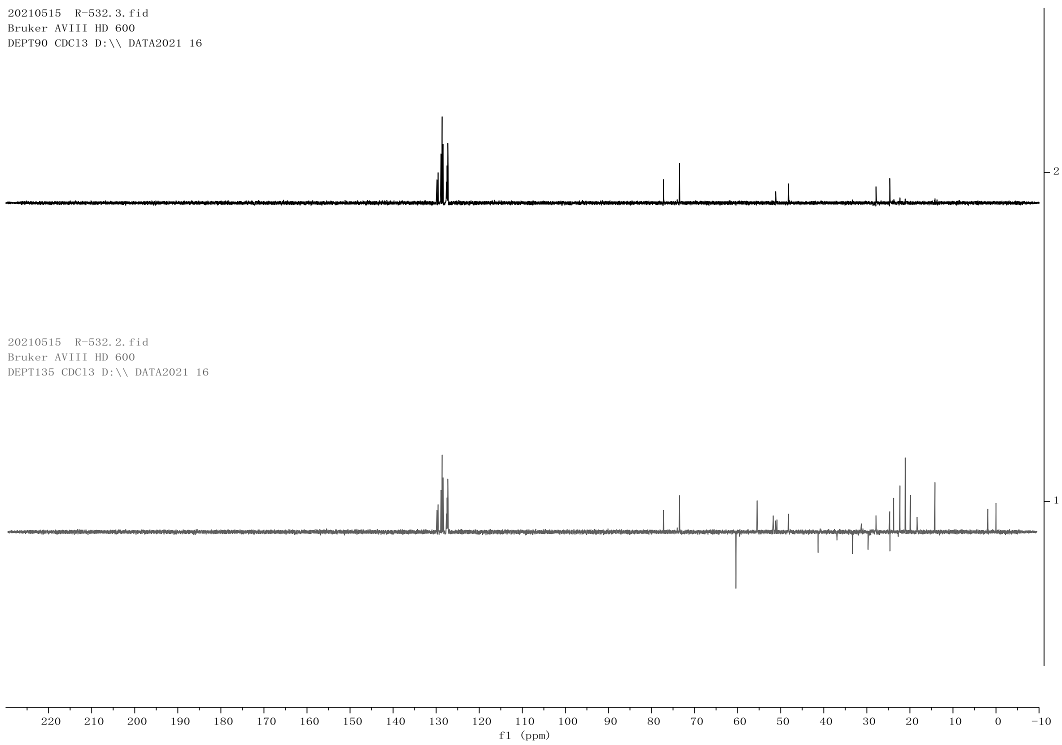

Figure S70. DEPT spectrum (150 MHz) of (*R*)-MTPA ester of mintaimycin B (**2**) in $CDCl_3$.

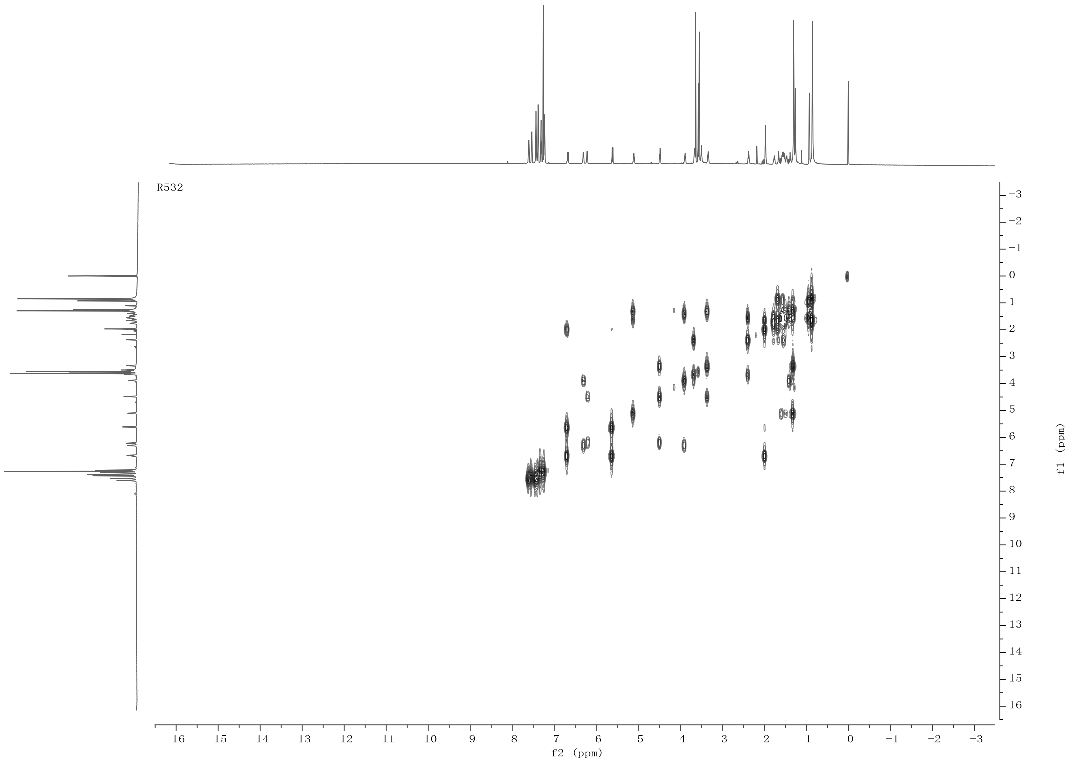

Figure S71. ^1H-^1H COSY spectrum (800 MHz) of (*R*)-MTPA ester of mintaimycin B (**2**) in $CDCl_3$.

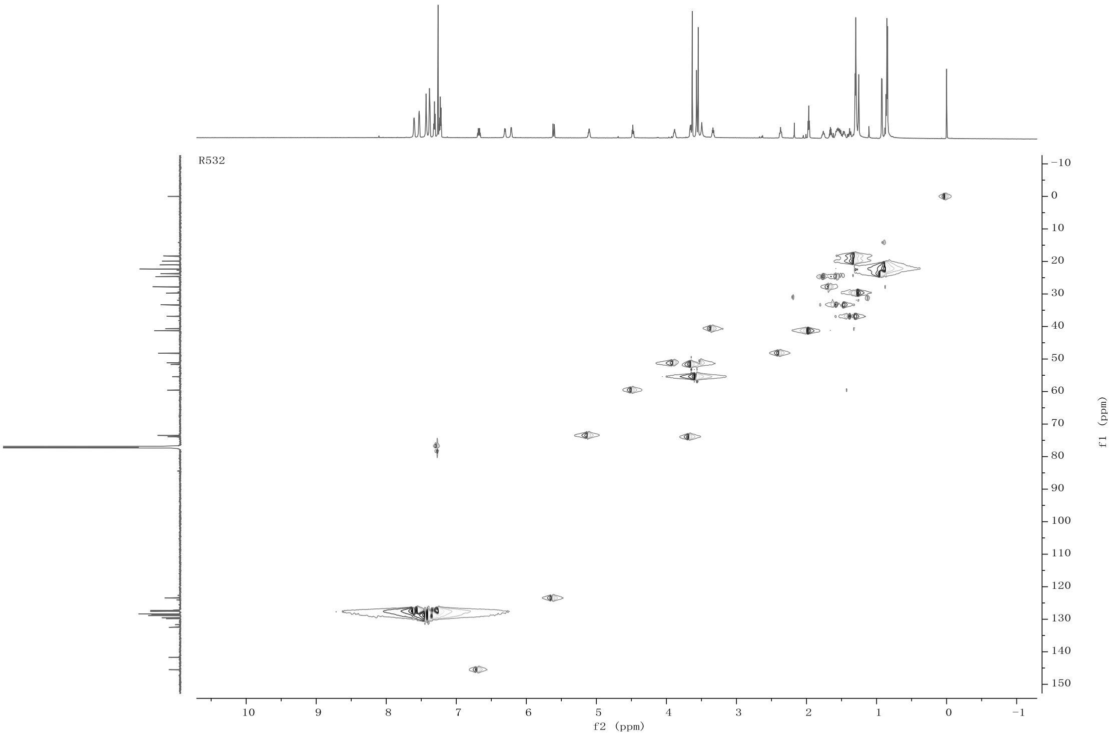

Figure S72. HSQC spectrum (200 MHz) of (*R*)-MTPA ester of mintaimycin B (**2**) in $CDCl_3$.

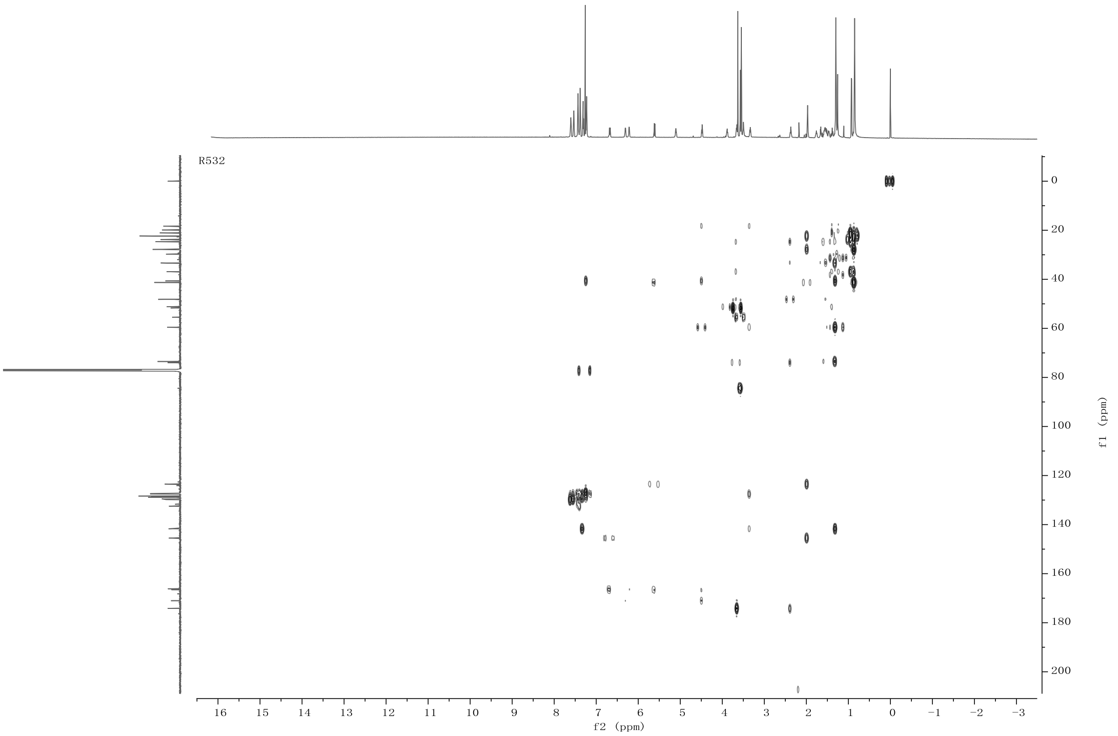

Figure S73. HMBC spectrum (200 MHz) of (*R*)-MTPA ester of mintaimycin B (**2**) in $CDCl_3$.

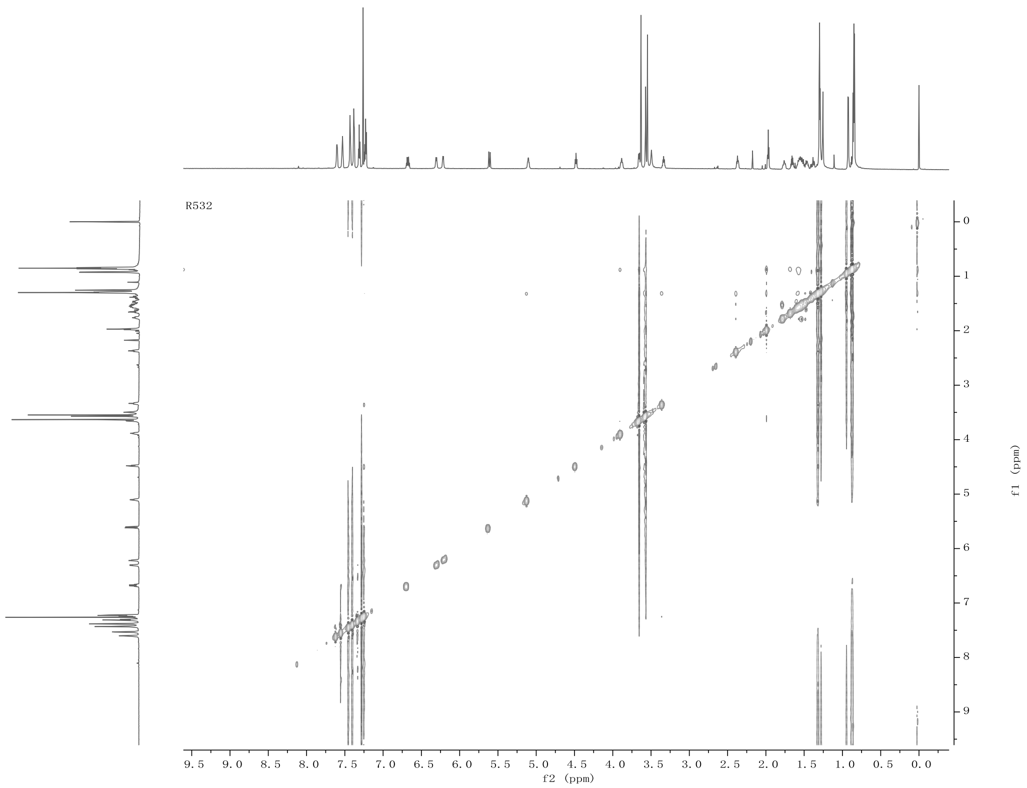

Figure S74. ROESY spectrum (800 MHz) of (*R*)-MTPA ester of mintaimycin B (**2**) in $CDCl_3$.

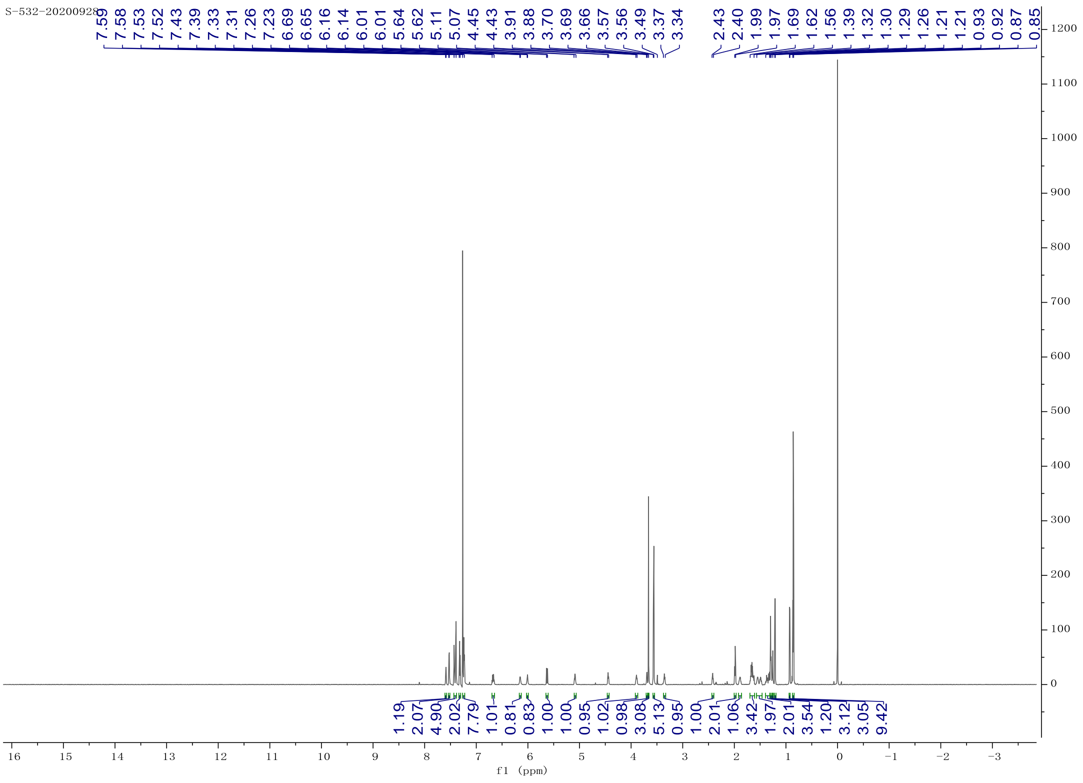

Figure S75. ^{1}H NMR spectrum (800 MHz) of (*S*)-MTPA ester of mintaimycin B (**2**) in $CDCl_3$.

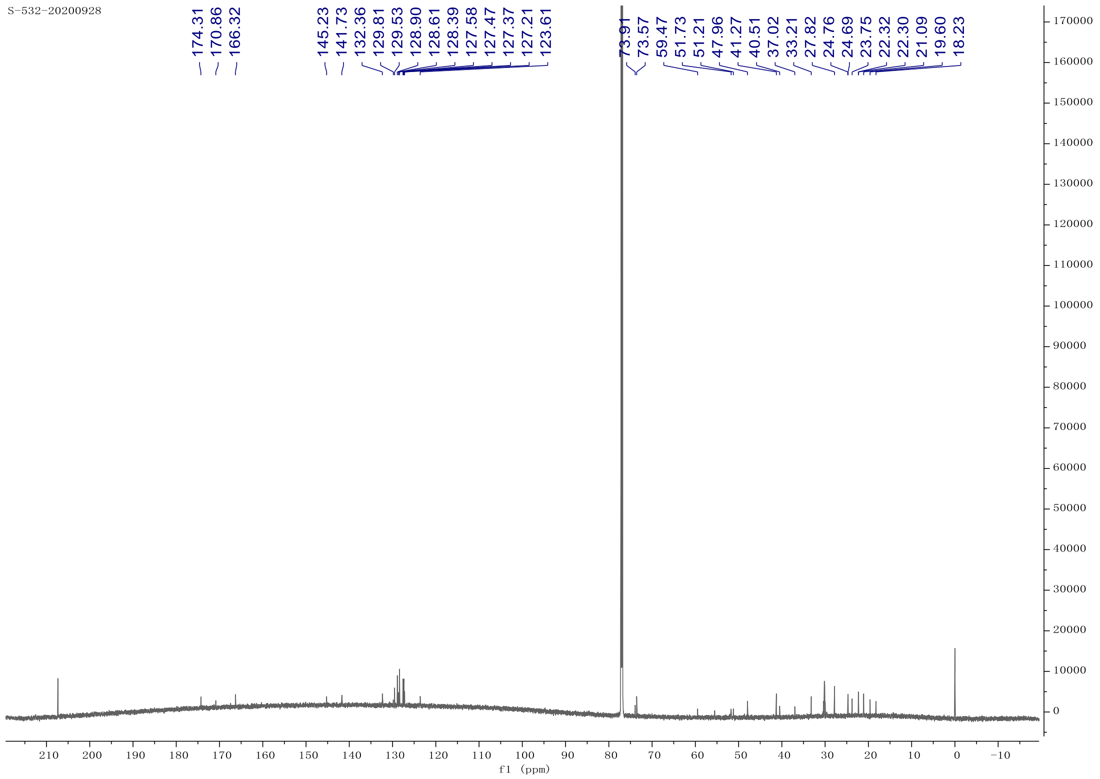

Figure S76. ^{13}C NMR spectrum (200 MHz) of (*S*)-MTPA ester of mintaimycin B (**2**) in $CDCl_3$.

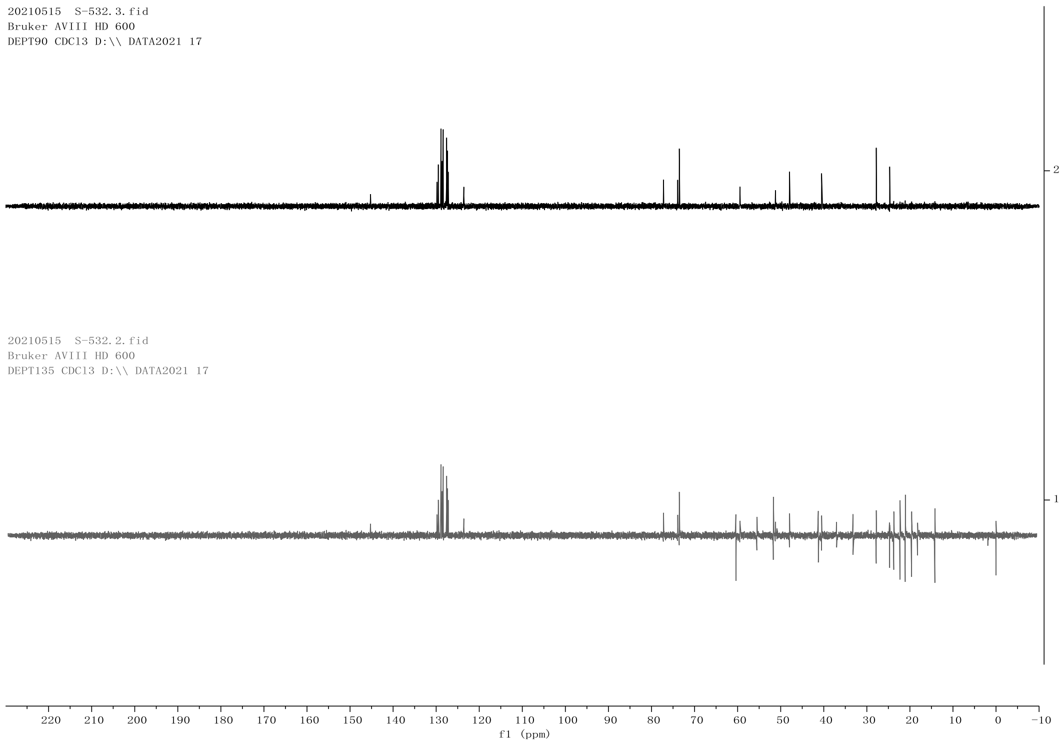

Figure S77. DEPT spectrum (150 MHz) of (*S*)-MTPA ester of mintaimycin B (**2**) in $CDCl_3$.

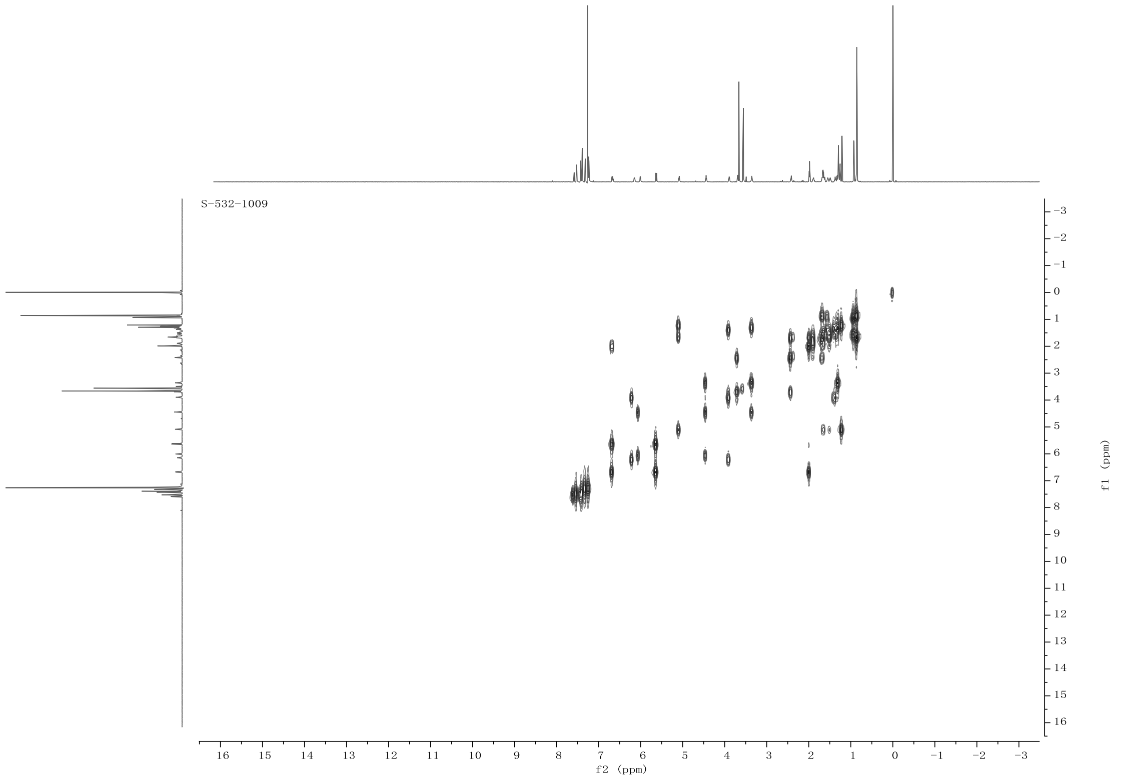

Figure S78. ^{1}H-^{1}H COSY spectrum (800 MHz) of (*S*)-MTPA ester of mintaimycin B (**2**) in $CDCl_3$.

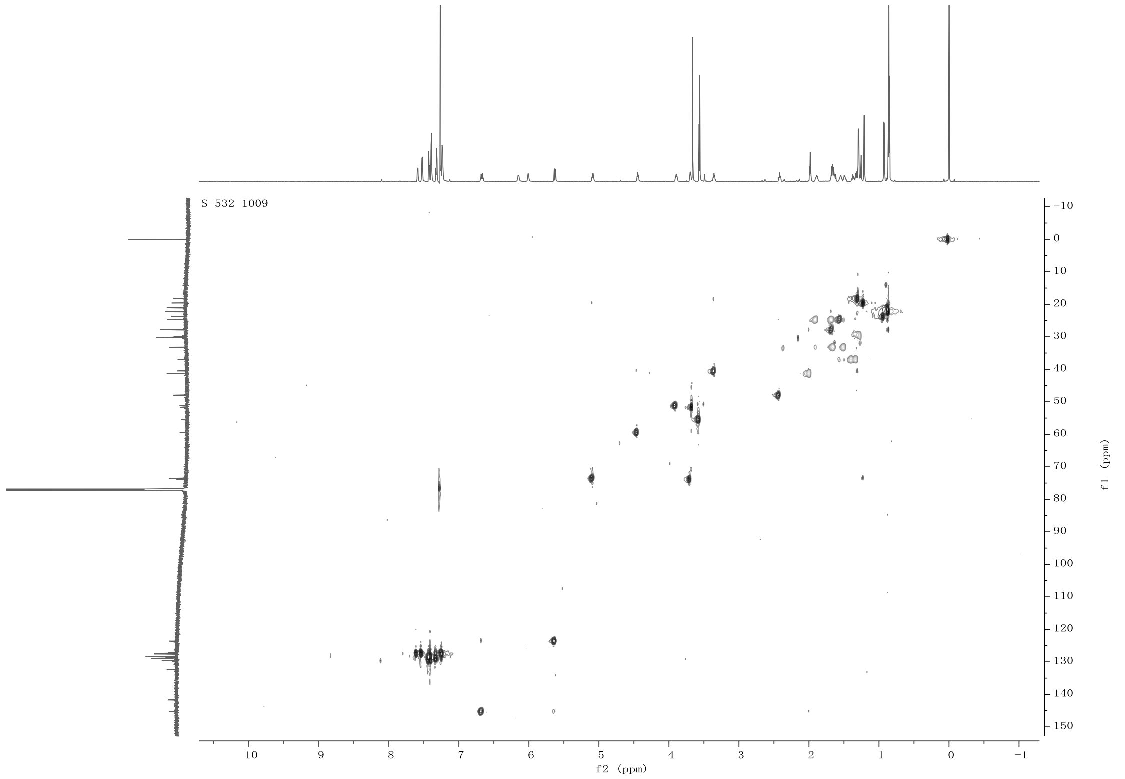

Figure S79. HSQC spectrum (200 MHz) of (*S*)-MTPA ester of mintaimycin B (**2**) in $CDCl_3$.

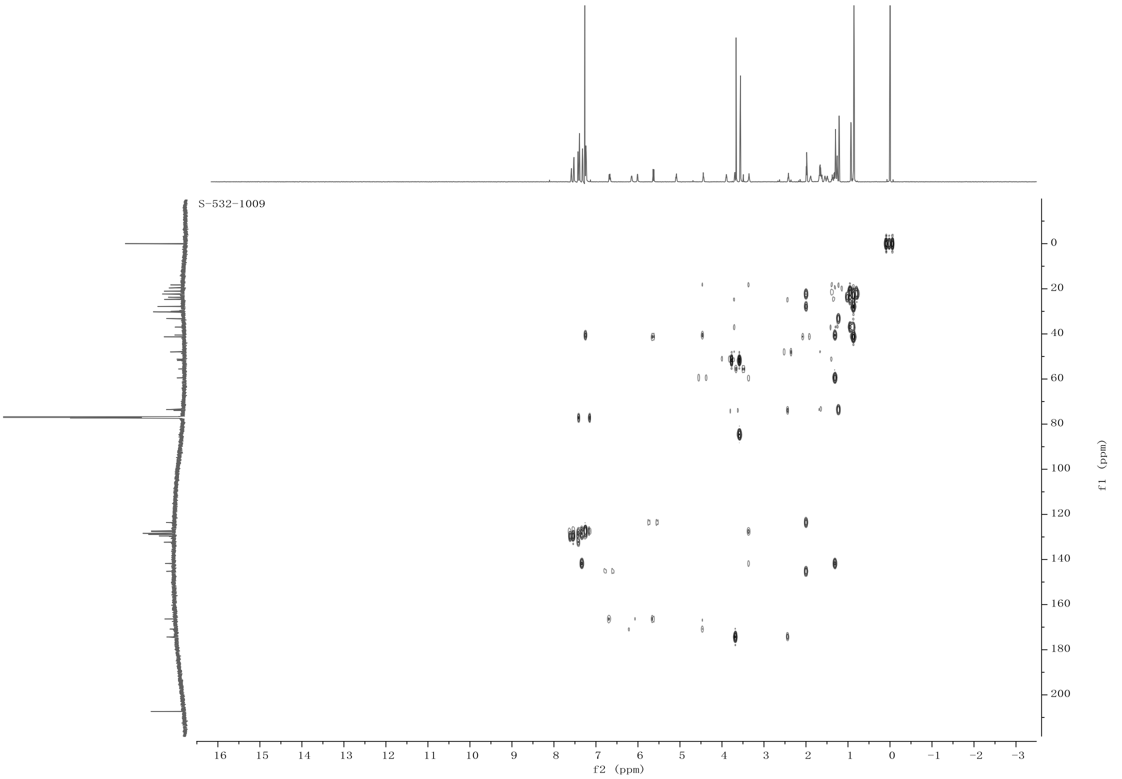

Figure S80. HMBC spectrum (200 MHz) of (*S*)-MTPA ester of mintaimycin B (**2**) in $CDCl_3$.

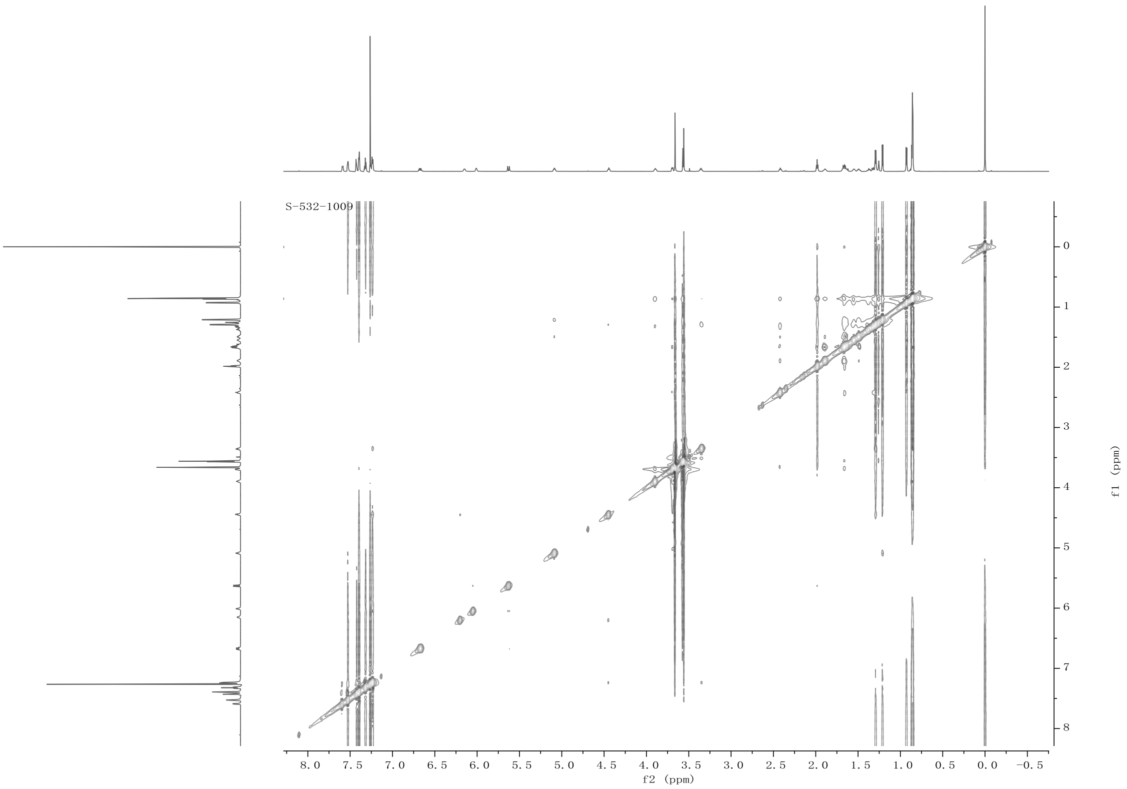

Figure S81. ROESY spectrum (800 MHz) of (*S*)-MTPA ester of mintaimycin B (**2**) in $CDCl_3$.

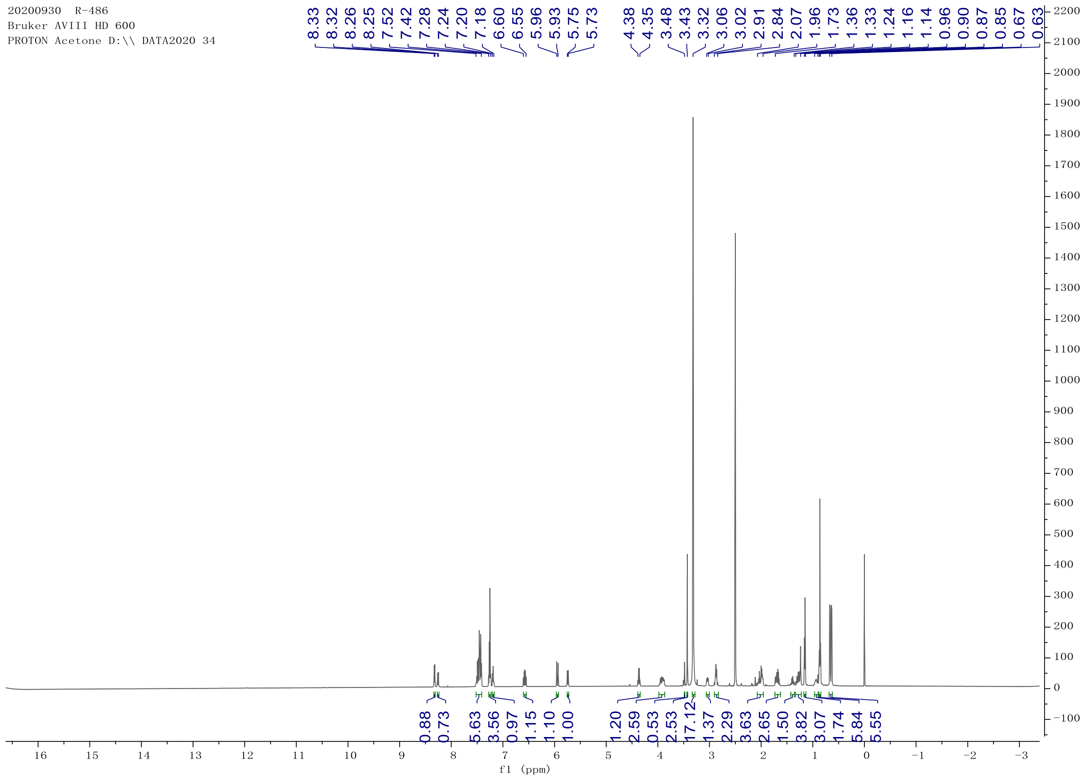

Figure S82. ^{1}H NMR spectrum (600 MHz) of (*R*)-MTPA ester of mintaimycin A_2 (**3**) in DMSO-d_6.

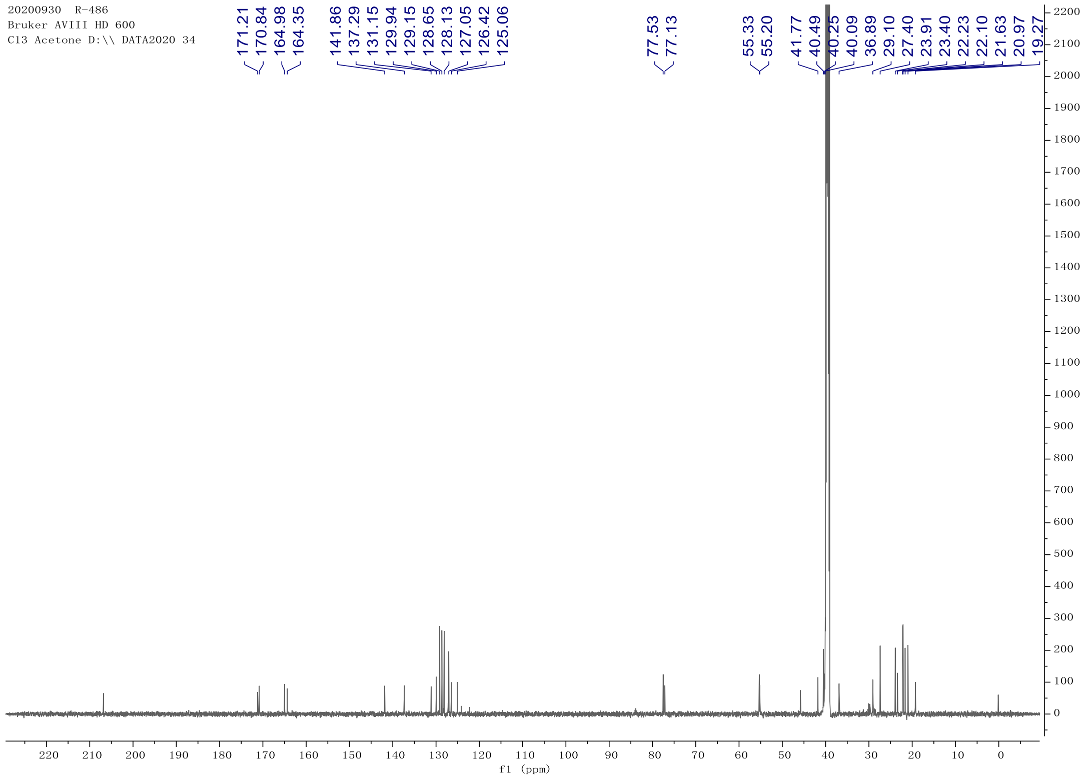

Figure S83. ^{13}C NMR spectrum (150 MHz) of (*R*)-MTPA ester of mintaimycin A_2 (**3**) in DMSO-d_6.

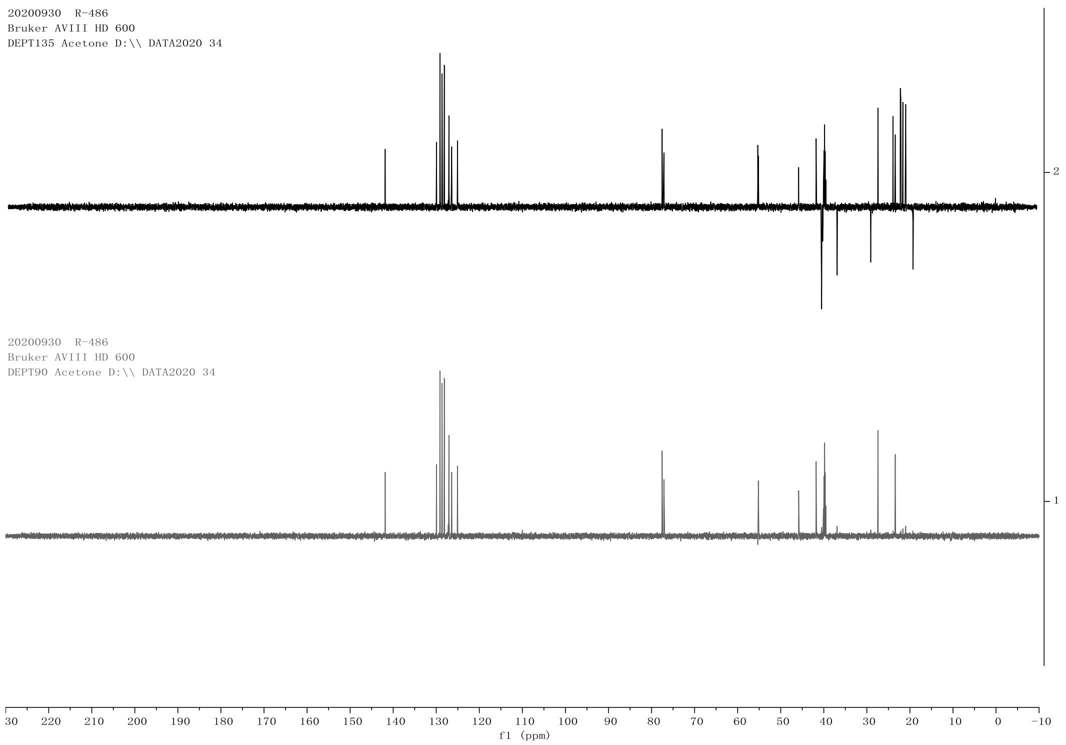

Figure S84. DEPT spectrum (150 MHz) of (*R*)-MTPA ester of mintaimycin A_2 (**3**) in DMSO-d_6.

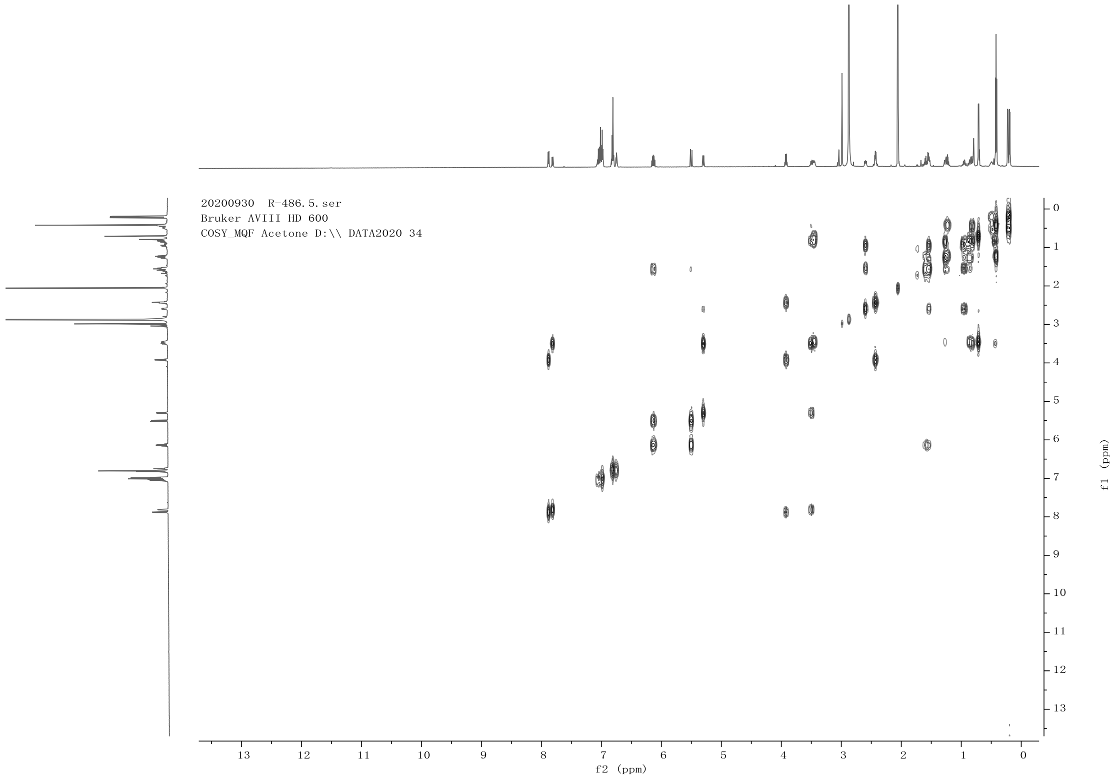

Figure S85. ^1H-^1H COSY spectrum (600 MHz) of (*R*)-MTPA ester of mintaimycin A_2 (**3**) in DMSO-d_6.

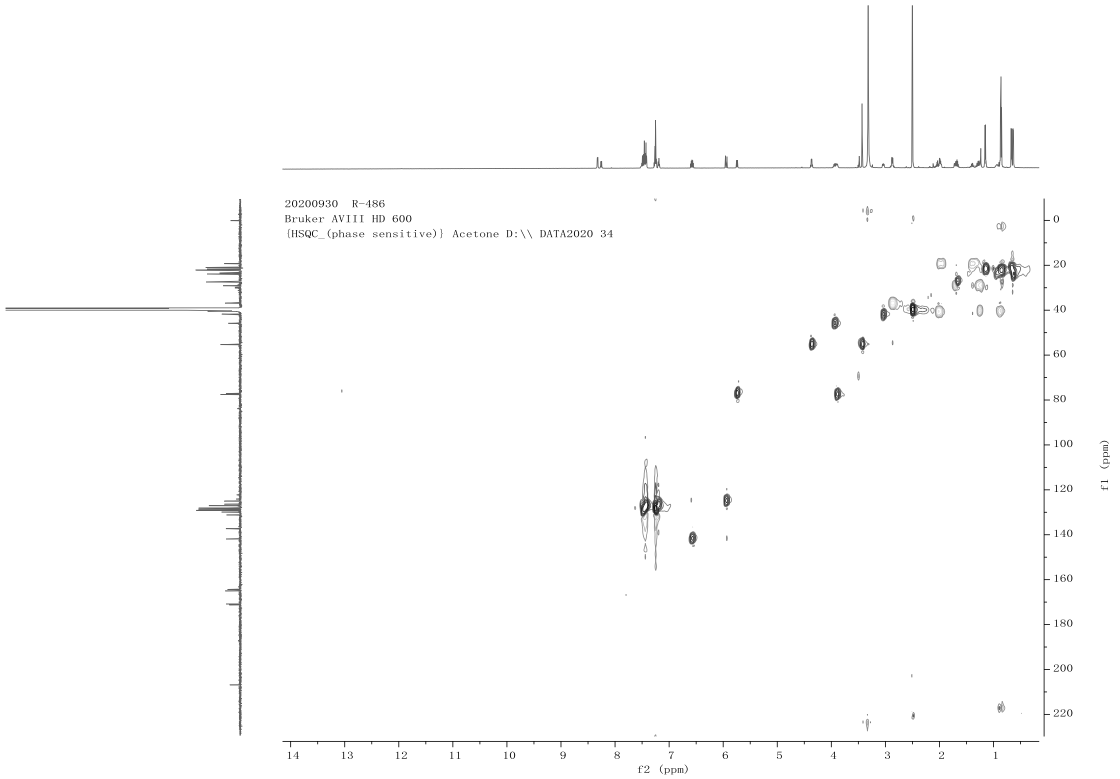

Figure S86. HSQC spectrum (150 MHz) of (*R*)-MTPA ester of mintaimycin A_2 (**3**) in DMSO-d_6.

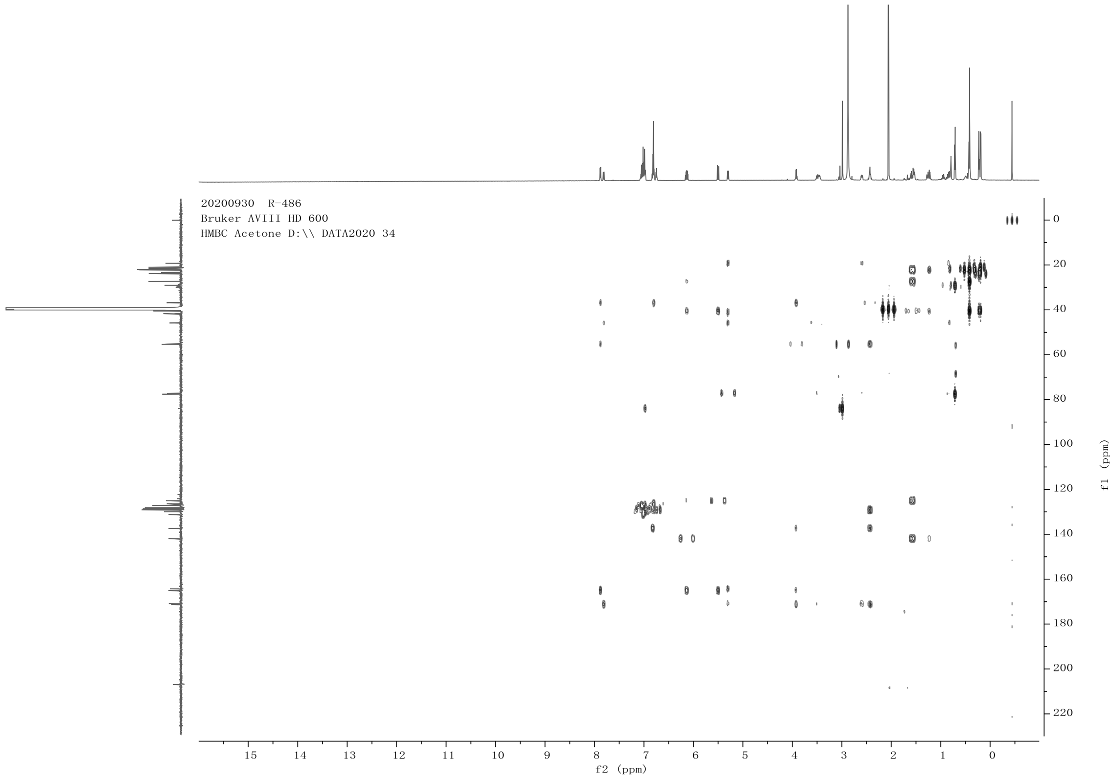

Figure S87. HMBC spectrum (150 MHz) of (*R*)-MTPA ester of mintaimycin A_2 (**3**) in DMSO-d_6.

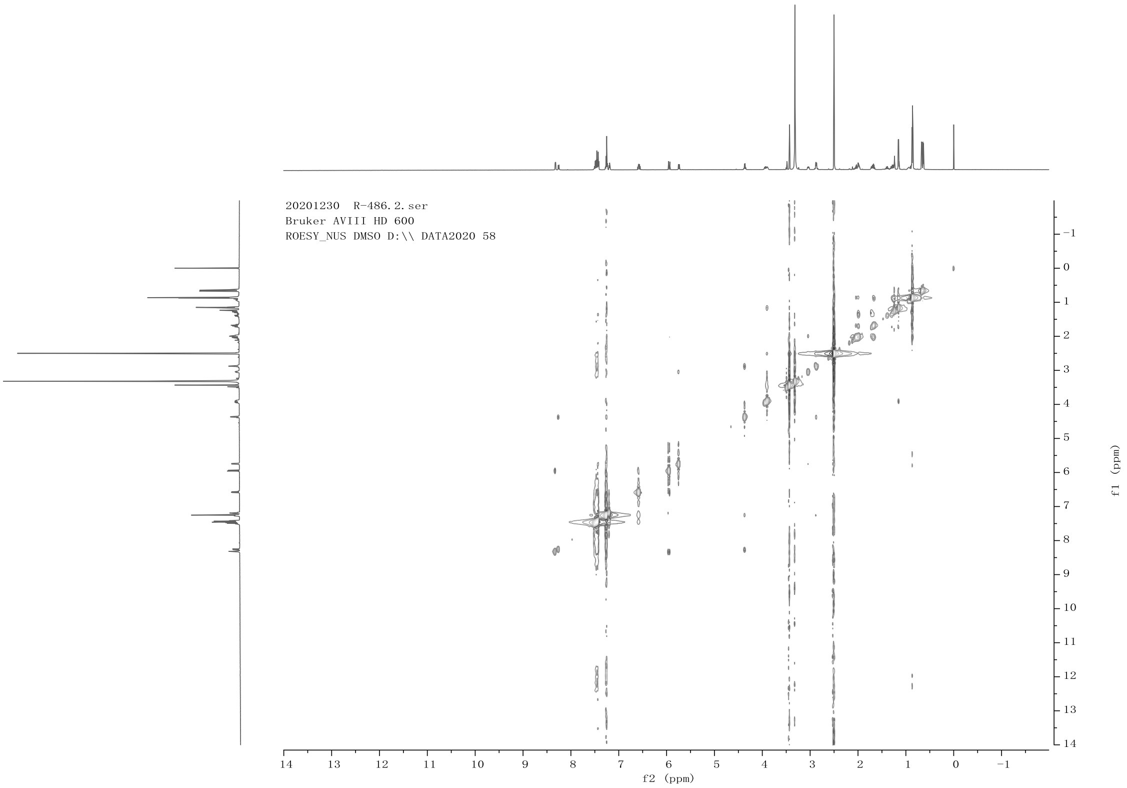

Figure S88. ROSY spectrum (600 MHz) of (*R*)-MTPA ester of mintaimycin A_2 (**3**) in DMSO-d_6.

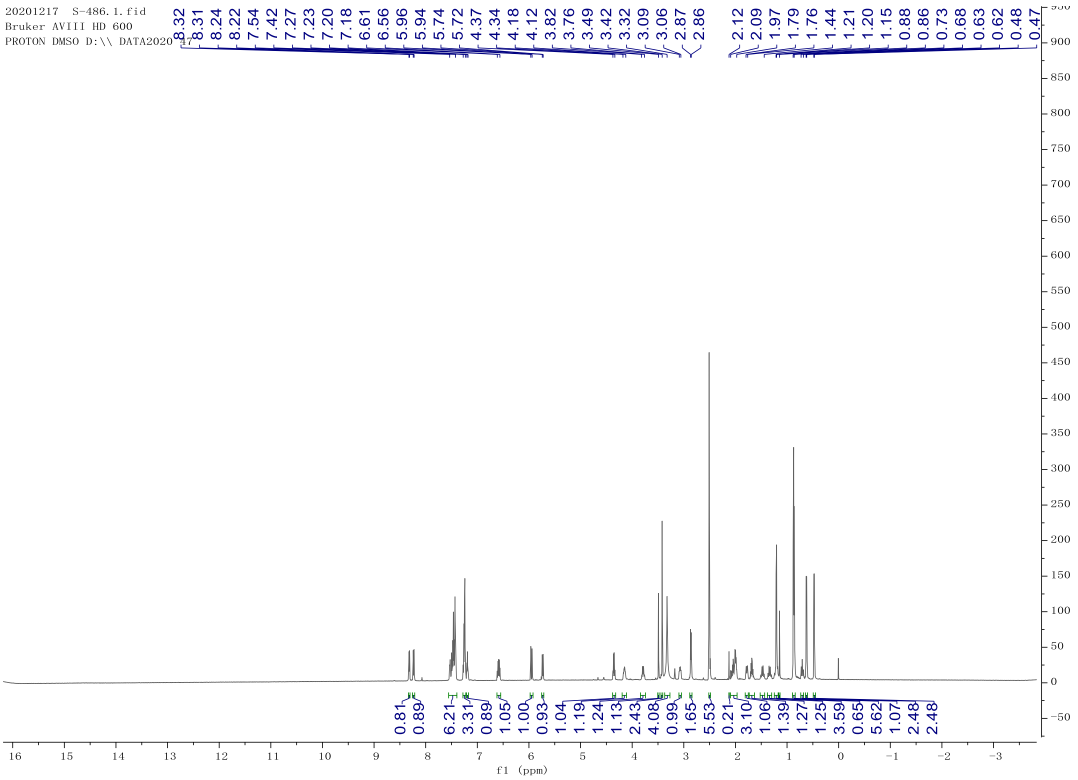

Figure S89. ^{1}H NMR spectrum (600 MHz) of (*S*)-MTPA ester of mintaimycin A_2 (**3**) in DMSO-d_6.

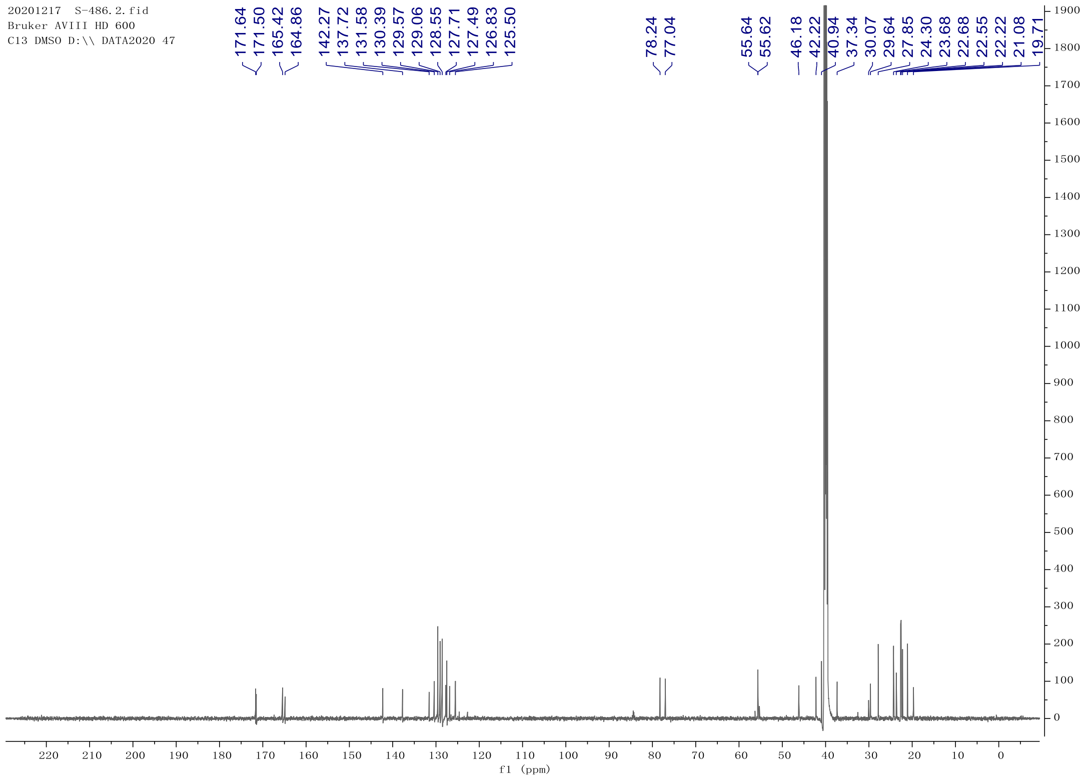

Figure S90. ^{13}C NMR spectrum (150 MHz) of (*S*)-MTPA ester of mintaimycin A_2 (**3**) in DMSO-d_6.

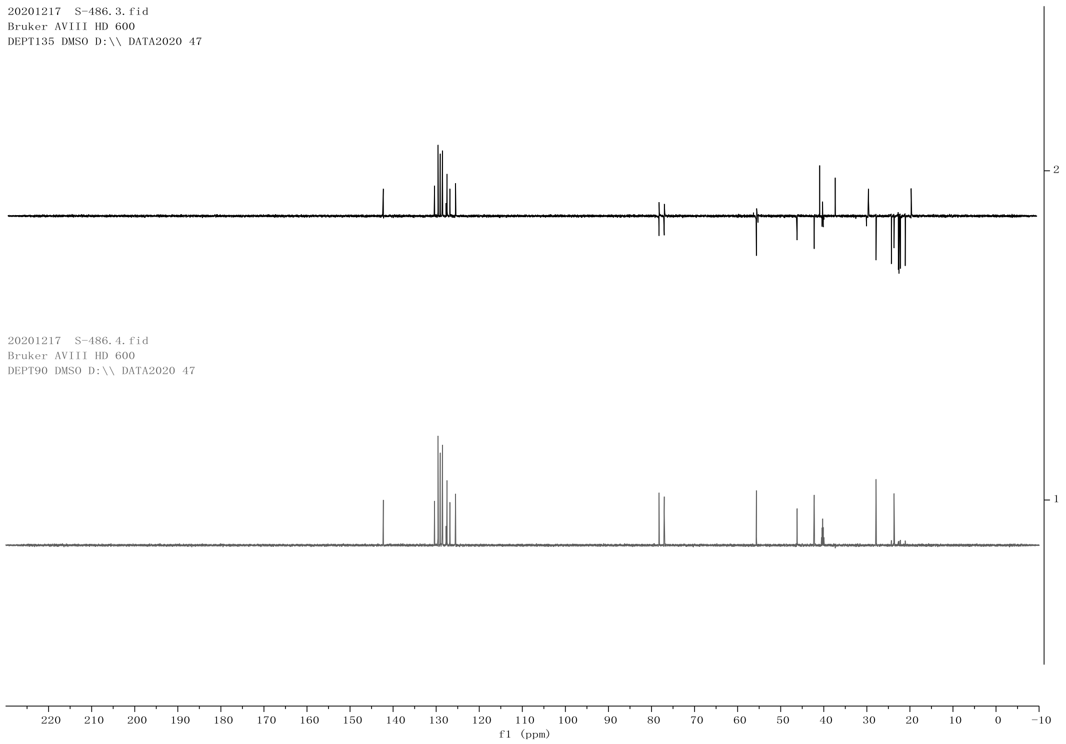

Figure S91. DEPT spectrum (150 MHz) of (*S*)-MTPA ester of mintaimycin A_2 (**3**) in DMSO-d_6.

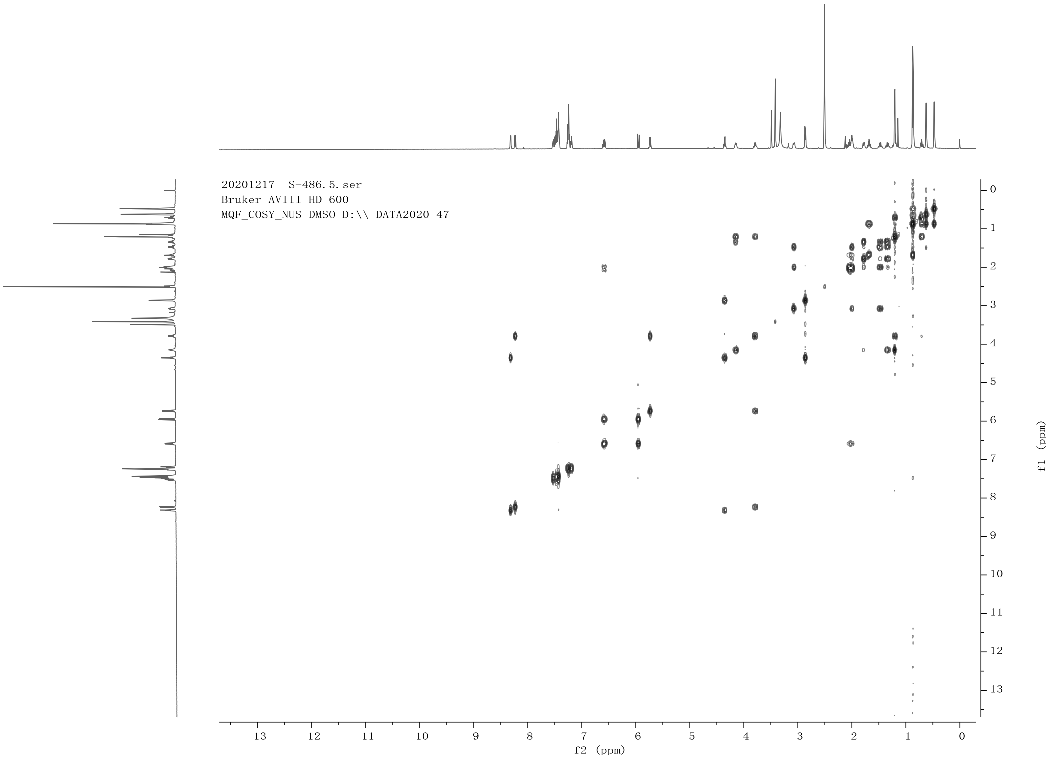

Figure S92. ^{1}H-^{1}H COSY spectrum (600 MHz) of (*S*)-MTPA ester of mintaimycin A_2 (**3**) in DMSO-d_6.

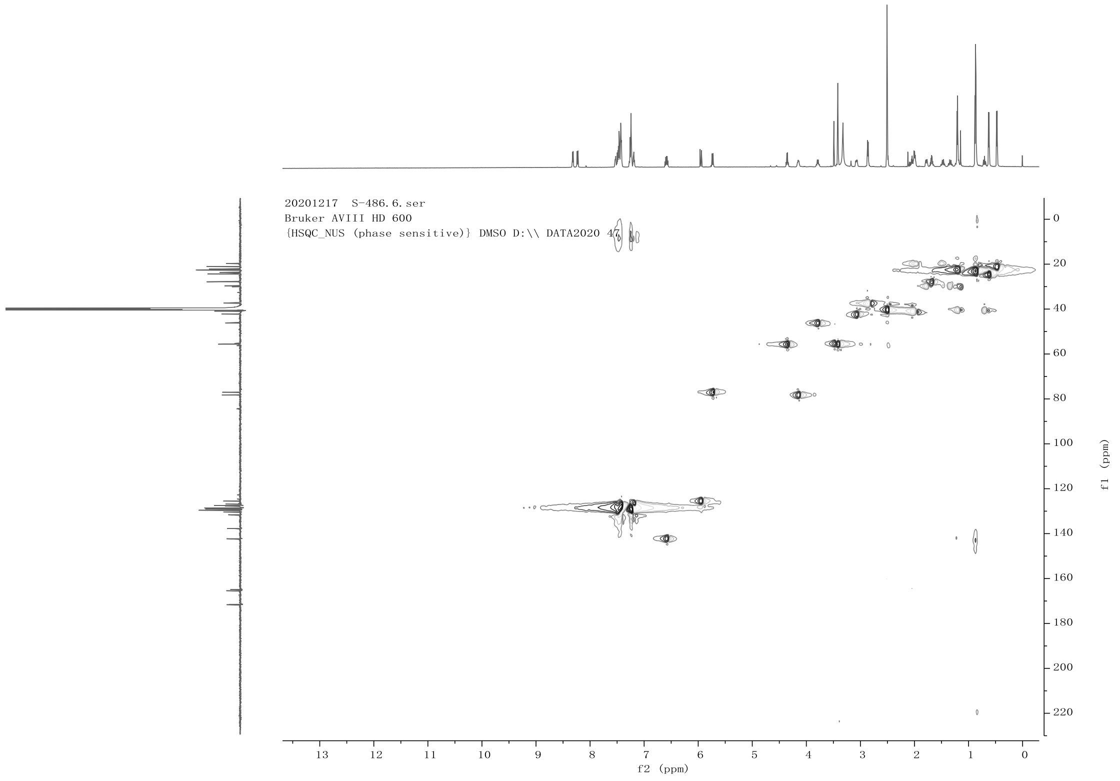

Figure S93. HSQC spectrum (150 MHz) of (*S*)-MTPA ester of mintaimycin A_2 (**3**) in DMSO-d_6.

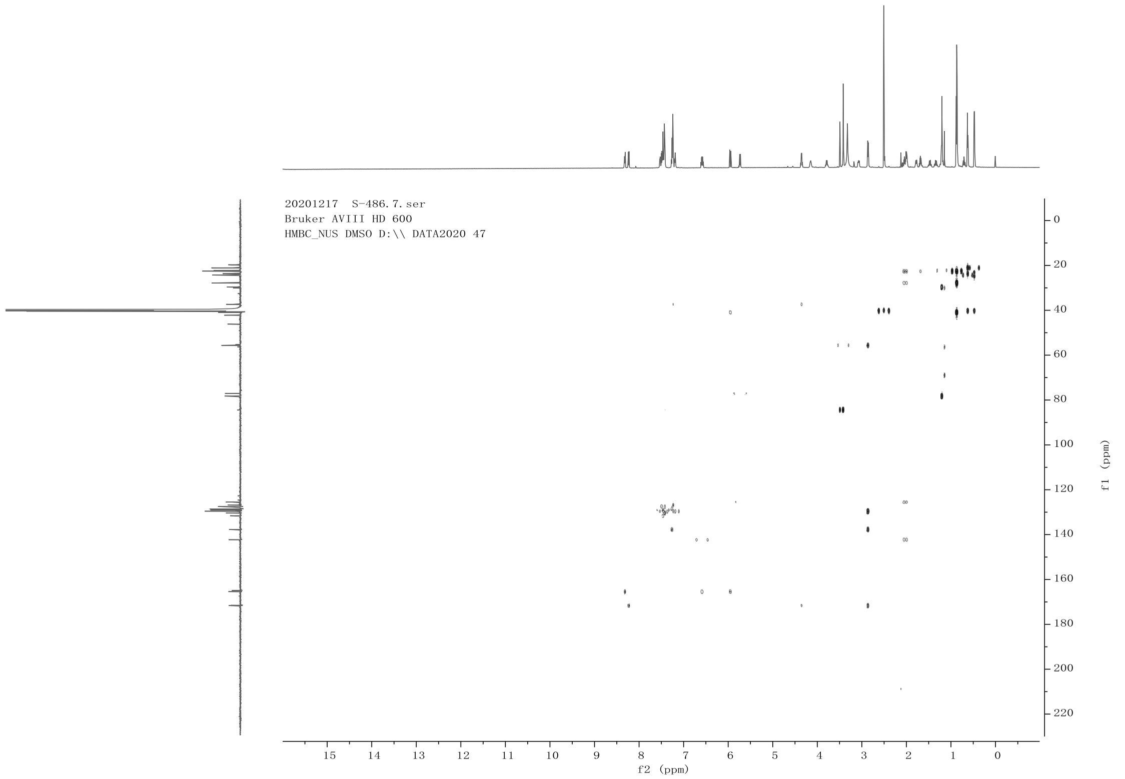

Figure S94. HMBC spectrum (150 MHz) of (*S*)-MTPA ester of mintaimycin A_2 (**3**) in DMSO-d_6.

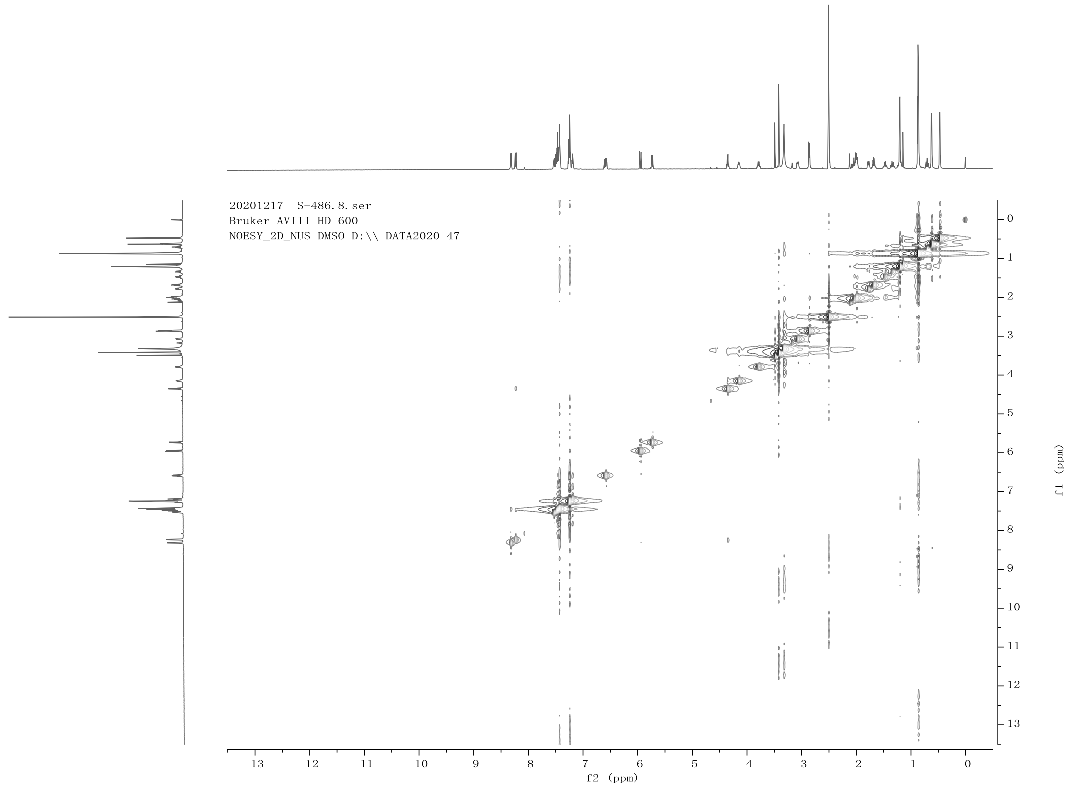

Figure S95. NOESY spectrum (600 MHz) of (*S*)-MTPA ester of mintaimycin A_2 (**3**) in DMSO-d_6.

Table S2. ^{1}H NMR data in Acetone-d_6 for the key protons of mintaimycin A$_1$ (**1**) and its (*R*)-MTPA and (*S*)-MTPA esters.

	1*R*	mintaimycin A$_1$ (**1**)	1*S*	
Position	δ_H (*J* in Hz)	δ_H (*J* in Hz)	δ_H (*J* in Hz)	$\Delta\delta^{SR}$
1"	1.19, d (6.0)	1.22, d (6.6)	1.28, d (6.4)	0.09
2"	3.93, m	4.28, m	4.18, m	0.25
3"	1.77, m; 1.39, m	1.90, m; 1.38, m	1.81, m; 1.41, m	0.04, 0.02
4"	2.15, m; 1.59, m	1.98, m; 1.89, m	2.16, m; 1.63, m	0.01, 0.04
5"	3.14, m	2.77, ddd (9.0, 7.2, 2.4)	3.16, m	0.02
6"	5.92, dd (10.4, 1.2)	4.16, ddd (8.4, 6.0, 1.8)	5.87, d (8.0)	-0.05
7"	4.34, m	4.03, m	4.15, m	-0.19
8"	1.62, m; 1.25, m	1.79, m; 1.38, m	1.50, m; 1.04, m	-0.12, -0.21
9"	1.77, m	1.79, m	1.66, m	-0.11
10"	0.91, d (6.6)	0.95, d (6.6)	0.84, d (8.0)	-0.07
11"	0.95, d (6.6)	0.93, d (6.6)	0.87, d (8.0)	-0.08

Table S3. ^{1}H NMR data in DMSO-d_6 for the key protons of mintaimycin A$_2$ (**3**) and its (*R*)-MTPA and (*S*)-MTPA esters.

	3*R*	mintaimycin A$_2$ (**3**)	3*S*	
Position	δ_H (*J* in Hz)	δ_H (*J* in Hz)	δ_H (*J* in Hz)	$\Delta\delta^{SR}$
1"	1.14, m	1.89, d (6.0)	1.21, m	0.07
2"	3.89, m	4.27, m	4.14, m	0.25
3"	1.70, m; 1.24, m	1.76, m; 1.26, m	1.78, m; 1.34, m	0.08, 0.10
4"	1.98, m; 1.26, m	1.83, m; 1.68, m	2.01, m; 1.50, m	0.03, 0.24
5"	3.04, m	2.65, m	3.07, m	0.03
6"	5.74, dd (10.4, 0.9)	3.94, ddd (9.0, 6.6, 1.8)	5.73, dd (10.8, 1.2)	-0.01
7"	3.93, m	3.64, m	3.79, m	-0.14
8"	2.02, m; 1.28, m	1.46, m; 1.13, m	1.20, m; 0.71, m	-0.82, -0.57
9"	1.67, m	1.14, m	0.87, m	-0.80
10"	0.85, d (6.0)	0.74, d (6.6)	0.48, d (6.0)	-0.37
11"	0.87, d (6.0)	0.74, d (6.6)	0.63, d (6.0)	-0.24

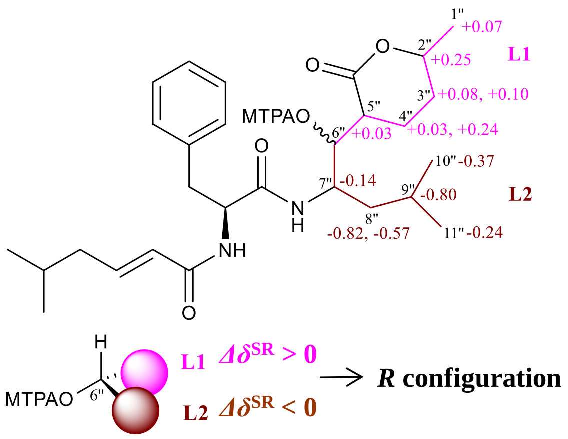

Figure S96. $\Delta\delta^{SR}$ values measured for the MTPA esters of mintaimycin A$_2$ (**3**).

Table S4. ^{1}H NMR data in $CDCl_3$ for the key protons of mintaimycin B (**2**) and its (*R*)-MTPA and (*S*)-MTPA esters.

	2*R*	mintaimycin B (**2**)	2*S*	
Position	δ_H (*J* in Hz)	δ_H (*J* in Hz)	δ_H (*J* in Hz)	$\Delta\delta^{SR}$
7"	3.88, m	4.10, overlap	3.90, m	0.02
8"	1.37, m;1.29, m	1.55, m; 1.26, m	1.39, m;1.32, m	0.02, 0.03
9"	1.57, m;1.45, m	1.89, m; 1.42, m	1.65, m;1.49, m	0.08, 0.04
10"	5.11, m	4.39, m	5.10, m	-0.01
11"	1.30, d (5.6)	1.31, d (7.2)	1.21, d (6.4)	-0.09

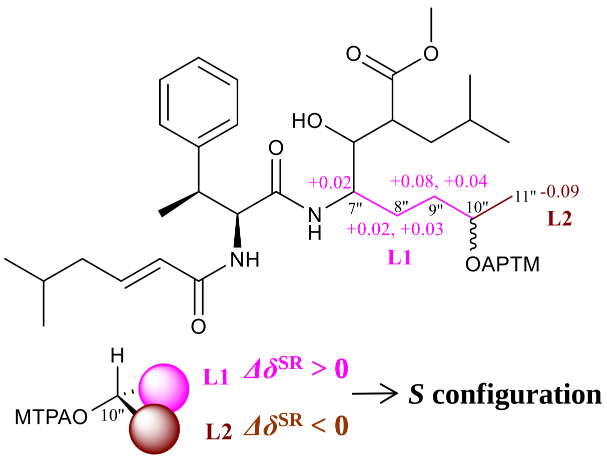

Figure S97. $\Delta\delta^{SR}$ values measured for the MTPA esters of mintaimycin B (**2**).

Table S5. MIC of mintaimycin A_1 (**1**), mintaimycin B (**2**) and mintaimycin A_2 (**3**) against Gram-positive and negative bacterial strains.

	Strain	MIC/μg · ml^{-1}			
		**1**	**2**	**3**	Levofloxacin
Gram-positive bacteria	*Staphylococcus epidermidis* ATCC 12228	>64	>64	>32	0.25
	Staphylococcus aureus ATCC 29213	>64	>64	>32	0.25
	Staphylococcus aureus ATCC 33591	>64	>64	>32	0.25
	Staphylococcus aureus ATCC 43300	>64	>64	>32	8
	Staphylococcus aureus ATCC 700698	>64	>64	>32	8
	Enterococcus faecalis ATCC 29212	>64	>64	>32	1
	Enterococcus faecalis ATCC 51299	>64	>64	>32	1
	Enterococcus faecalis ATCC 51575	>64	>64	>32	1
	Excrement enterococcus ATCC 700221	>64	>64	>32	64
Gram-negative bacteria	*Escherichia coli* ATCC 25922	>64	>64	>32	≤0.03
	Escherichia coli ATCC 35218	>64	>64	>32	0.06
	Klebsiella pneumonia ATCC 700603	>64	>64	>32	0.5
	Klebsiella pneumonia ATCC BAA-2146	>64	>64	>32	>64
	Pseudomonas aeruginosa ATCC 27853	>64	>64	>32	1
	Acinetobacter baumannii ATCC 19606	>64	>64	>32	0.25
	Enterobacter cloacae ATCC 43560	>64	>64	>32	≤0.03
	Enterobacter aerogenes ATCC 13048	>64	>64	>32	0.12
	Serratia marcescens ATCC 21074	>64	>64	>32	0.12
	Proteus singular ATCC 49565	>64	>64	>32	0.06
	Pseudomonas maltophilia ATCC 13636	>64	>64	>32	1
	Shigella flexneri ATCC 12022	>64	>64	>32	≤0.03

A brief description of antibacterial activity assay. Minimum inhibitory concentrations (MICs) of **1-3** were determined by the agar dilution method from Clinical Laboratory Standards Institute. The test microorganisms include ATCC bacterial strains and clinical isolates. The test medium was Mueller-Hinton broth, with an inoculum of 10^3 colony forming units for each well. The final concentrations of **1-2** and **3** ranged from 0.03 to 64 μg/mL and 0.03 to 32 μg/mL, respectively. The culture plates were incubated at 35 ℃ for 18 h. The MIC was defined as the lowest concentration that prevented visible growth of bacteria. Levofloxacin (LEV) was used as positive control in the assay.

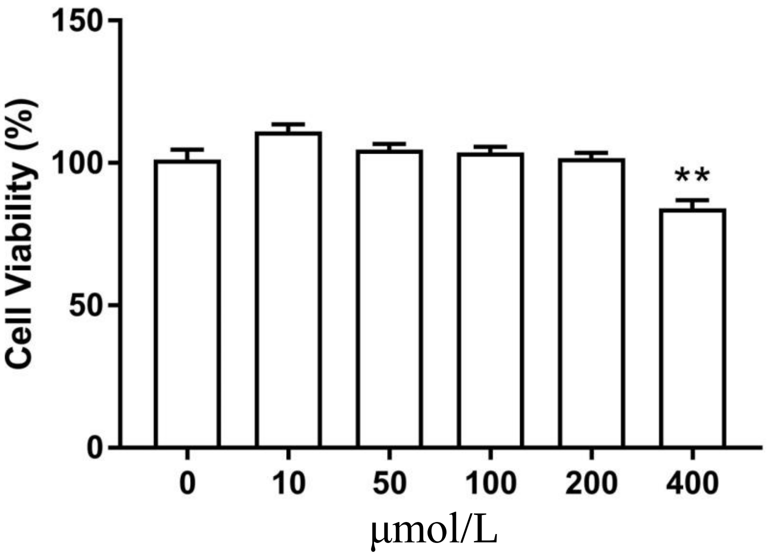

Figure S98. Viability of 3T3-L1 fibroblast cells treated with various concentrations of mintaimycin A_1 (**1**). A concentration up to 200 μmmol/L mintaimycin A_1 (**1**) displayed no inhibitive effect on the viability of 3T3-L1 fibroblast cells.

Table S6. IC_{50} of mintaimycin A_1 (**1**) against human cell lines.

	IC_{50} (μmol/L)		
compound	Hela	HGC-27	PANC-1
mintaimycin A_1 (**1**)	245.6 ±11.04	191.32 ±18.34	445.74 ±33.16

A brief description of cytotoxicity assay. Stock solution of **1**, dissolved in DMSO at 10.0 mg/mL (20 mmol/L), was used to evaluate its cytotoxicity against human cervical cancer cell line (Hela), gastric carcinoma cell line (HGC-27), and pancreatic cancer cell line (PANC-1) by MTT assay. The assays were conducted at 12.5, 25, 50, 100, 200 μg/mL (25, 50, 100, 200, 400 μmol/L) for **1**. IC_{50} values were calculated for **1**.

Reference: Sha MQ, Zhao XL, Li L, Li LH, Li Y, Dong TG, Niu WX, Jia LJ, Shao RG, Zhen YS, Wang Z. *Cell Death Dis.* **2016**, 24;7(11): e2486.
